# Precise determination of the mass of the Higgs boson and tests of compatibility of its couplings with the standard model predictions using proton collisions at 7 and 8$\,\text {TeV}$

**DOI:** 10.1140/epjc/s10052-015-3351-7

**Published:** 2015-05-14

**Authors:** V. Khachatryan, A. M. Sirunyan, A. Tumasyan, W. Adam, T. Bergauer, M. Dragicevic, J. Erö, M. Friedl, R. Frühwirth, V. M. Ghete, C. Hartl, N. Hörmann, J. Hrubec, M. Jeitler, W. Kiesenhofer, V. Knünz, M. Krammer, I. Krätschmer, D. Liko, I. Mikulec, D. Rabady, B. Rahbaran, H. Rohringer, R. Schöfbeck, J. Strauss, W. Treberer-Treberspurg, W. Waltenberger, C.-E. Wulz, V. Mossolov, N. Shumeiko, J. Suarez Gonzalez, S. Alderweireldt, S. Bansal, T. Cornelis, E. A. De Wolf, X. Janssen, A. Knutsson, J. Lauwers, S. Luyckx, S. Ochesanu, R. Rougny, M. Van De Klundert, H. Van Haevermaet, P. Van Mechelen, N. Van Remortel, A. Van Spilbeeck, F. Blekman, S. Blyweert, J. D’Hondt, N. Daci, N. Heracleous, J. Keaveney, S. Lowette, M. Maes, A. Olbrechts, Q. Python, D. Strom, S. Tavernier, W. Van Doninck, P. Van Mulders, G. P. Van Onsem, I. Villella, C. Caillol, B. Clerbaux, G. De Lentdecker, D. Dobur, L. Favart, A. P. R. Gay, A. Grebenyuk, A. Léonard, A. Mohammadi, L. Perniè, A. Randle-conde, T. Reis, T. Seva, L. Thomas, C. Vander Velde, P. Vanlaer, J. Wang, F. Zenoni, V. Adler, K. Beernaert, L. Benucci, A. Cimmino, S. Costantini, S. Crucy, A. Fagot, G. Garcia, J. Mccartin, A. A. Ocampo Rios, D. Poyraz, D. Ryckbosch, S. Salva Diblen, M. Sigamani, N. Strobbe, F. Thyssen, M. Tytgat, E. Yazgan, N. Zaganidis, S. Basegmez, C. Beluffi, G. Bruno, R. Castello, A. Caudron, L. Ceard, G. G. Da Silveira, C. Delaere, T. du Pree, D. Favart, L. Forthomme, A. Giammanco, J. Hollar, A. Jafari, P. Jez, M. Komm, V. Lemaitre, C. Nuttens, D. Pagano, L. Perrini, A. Pin, K. Piotrzkowski, A. Popov, L. Quertenmont, M. Selvaggi, M. Vidal Marono, J. M. Vizan Garcia, N. Beliy, T. Caebergs, E. Daubie, G. H. Hammad, W. L. Aldá Júnior, G. A. Alves, L. Brito, M. Correa Martins Junior, T. Dos Reis Martins, J. Molina, C. Mora Herrera, M. E. Pol, P. Rebello Teles, W. Carvalho, J. Chinellato, A. Custódio, E. M. Da Costa, D. De Jesus Damiao, C. De Oliveira Martins, S. Fonseca De Souza, H. Malbouisson, D. Matos Figueiredo, L. Mundim, H. Nogima, W. L. Prado Da Silva, J. Santaolalla, A. Santoro, A. Sznajder, E. J. Tonelli Manganote, A. Vilela Pereira, C. A. Bernardes, S. Dogra, T. R. Fernandez Perez Tomei, E. M. Gregores, P. G. Mercadante, S. F. Novaes, Sandra S. Padula, A. Aleksandrov, V. Genchev, R. Hadjiiska, P. Iaydjiev, A. Marinov, S. Piperov, M. Rodozov, S. Stoykova, G. Sultanov, M. Vutova, A. Dimitrov, I. Glushkov, L. Litov, B. Pavlov, P. Petkov, J. G. Bian, G. M. Chen, H. S. Chen, M. Chen, T. Cheng, R. Du, C. H. Jiang, R. Plestina, F. Romeo, J. Tao, Z. Wang, C. Asawatangtrakuldee, Y. Ban, S. Liu, Y. Mao, S. J. Qian, D. Wang, Z. Xu, F. Zhang, L. Zhang, W. Zou, C. Avila, A. Cabrera, L. F. Chaparro Sierra, C. Florez, J. P. Gomez, B. Gomez Moreno, J. C. Sanabria, N. Godinovic, D. Lelas, D. Polic, I. Puljak, Z. Antunovic, M. Kovac, V. Brigljevic, K. Kadija, J. Luetic, D. Mekterovic, L. Sudic, A. Attikis, G. Mavromanolakis, J. Mousa, C. Nicolaou, F. Ptochos, P. A. Razis, H. Rykaczewski, M. Bodlak, M. Finger, M. Finger, Y. Assran, A. Ellithi Kame, M. A. Mahmoud, A. Radi, M. Kadastik, M. Murumaa, M. Raidal, A. Tiko, P. Eerola, M. Voutilainen, J. Härkönen, J. K. Heikkilä, V. Karimäki, R. Kinnunen, M. J. Kortelainen, T. Lampén, K. Lassila-Perini, S. Lehti, T. Lindén, P. Luukka, T. Mäenpää, T. Peltola, E. Tuominen, J. Tuominiemi, E. Tuovinen, L. Wendland, J. Talvitie, T. Tuuva, M. Besancon, F. Couderc, M. Dejardin, D. Denegri, B. Fabbro, J. L. Faure, C. Favaro, F. Ferri, S. Ganjour, A. Givernaud, P. Gras, G. Hamel de Monchenault, P. Jarry, E. Locci, J. Malcles, J. Rander, A. Rosowsky, M. Titov, S. Baffioni, F. Beaudette, P. Busson, E. Chapon, C. Charlot, T. Dahms, L. Dobrzynski, N. Filipovic, A. Florent, R. Granier de Cassagnac, L. Mastrolorenzo, P. Miné, I. N. Naranjo, M. Nguyen, C. Ochando, G. Ortona, P. Paganini, S. Regnard, R. Salerno, J. B. Sauvan, Y. Sirois, C. Veelken, Y. Yilmaz, A. Zabi, J.-L. Agram, J. Andrea, A. Aubin, D. Bloch, J.-M. Brom, E. C. Chabert, C. Collard, E. Conte, J.-C. Fontaine, D. Gelé, U. Goerlach, C. Goetzmann, A.-C. Le Bihan, K. Skovpen, P. Van Hove, S. Gadrat, S. Beauceron, N. Beaupere, C. Bernet, G. Boudoul, E. Bouvier, S. Brochet, C. A. Carrillo Montoya, J. Chasserat, R. Chierici, D. Contardo, B. Courbon, P. Depasse, H. El Mamouni, J. Fan, J. Fay, S. Gascon, M. Gouzevitch, B. Ille, T. Kurca, M. Lethuillier, L. Mirabito, A. L. Pequegnot, S. Perries, J. D. Ruiz Alvarez, D. Sabes, L. Sgandurra, V. Sordini, M. Vander Donckt, P. Verdier, S. Viret, H. Xiao, Z. Tsamalaidze, C. Autermann, S. Beranek, M. Bontenackels, M. Edelhoff, L. Feld, A. Heister, K. Klein, M. Lipinski, A. Ostapchuk, M. Preuten, F. Raupach, J. Sammet, S. Schael, J. F. Schulte, H. Weber, B. Wittmer, V. Zhukov, M. Ata, M. Brodski, E. Dietz-Laursonn, D. Duchardt, M. Erdmann, R. Fischer, A. Güth, T. Hebbeker, C. Heidemann, K. Hoepfner, D. Klingebiel, S. Knutzen, P. Kreuzer, M. Merschmeyer, A. Meyer, P. Millet, M. Olschewski, K. Padeken, P. Papacz, H. Reithler, S. A. Schmitz, L. Sonnenschein, D. Teyssier, S. Thüer, V. Cherepanov, Y. Erdogan, G. Flügge, H. Geenen, M. Geisler, W. Haj Ahmad, F. Hoehle, B. Kargoll, T. Kress, Y. Kuessel, A. Künsken, J. Lingemann, A. Nowack, I. M. Nugent, C. Pistone, O. Pooth, A. Stahl, M. Aldaya Martin, I. Asin, N. Bartosik, J. Behr, U. Behrens, A. J. Bell, A. Bethani, K. Borras, A. Burgmeier, A. Cakir, L. Calligaris, A. Campbell, S. Choudhury, F. Costanza, C. Diez Pardos, G. Dolinska, S. Dooling, T. Dorland, G. Eckerlin, D. Eckstein, T. Eichhorn, G. Flucke, J. Garay Garcia, A. Geiser, A. Gizhko, P. Gunnellini, J. Hauk, M. Hempel, H. Jung, A. Kalogeropoulos, O. Karacheban, M. Kasemann, P. Katsas, J. Kieseler, C. Kleinwort, I. Korol, D. Krücker, W. Lange, J. Leonard, K. Lipka, A. Lobanov, W. Lohmann, B. Lutz, R. Mankel, I. Marfin, I.-A. Melzer-Pellmann, A. B. Meyer, G. Mittag, J. Mnich, A. Mussgiller, S. Naumann-Emme, A. Nayak, E. Ntomari, H. Perrey, D. Pitzl, R. Placakyte, A. Raspereza, P. M. Ribeiro Cipriano, B. Roland, E. Ron, M. Ö. Sahin, J. Salfeld-Nebgen, P. Saxena, T. Schoerner-Sadenius, M. Schröder, C. Seitz, S. Spannagel, A. D. R. Vargas Trevino, R. Walsh, C. Wissing, V. Blobel, M. Centis Vignali, A. R. Draeger, J. Erfle, E. Garutti, K. Goebel, M. Görner, J. Haller, M. Hoffmann, R. S. Höing, A. Junkes, H. Kirschenmann, R. Klanner, R. Kogler, T. Lapsien, T. Lenz, I. Marchesini, D. Marconi, J. Ott, T. Peiffer, A. Perieanu, N. Pietsch, J. Poehlsen, T. Poehlsen, D. Rathjens, C. Sander, H. Schettler, P. Schleper, E. Schlieckau, A. Schmidt, M. Seidel, V. Sola, H. Stadie, G. Steinbrück, D. Troendle, E. Usai, L. Vanelderen, A. Vanhoefer, C. Barth, C. Baus, J. Berger, C. Böser, E. Butz, T. Chwalek, W. De Boer, A. Descroix, A. Dierlamm, M. Feindt, F. Frensch, M. Giffels, A. Gilbert, F. Hartmann, T. Hauth, U. Husemann, I. Katkov, A. Kornmayer, P. Lobelle Pardo, M. U. Mozer, T. Müller, Th. Müller, A. Nürnberg, G. Quast, K. Rabbertz, S. Röcker, H. J. Simonis, F. M. Stober, R. Ulrich, J. Wagner-Kuhr, S. Wayand, T. Weiler, R. Wolf, G. Anagnostou, G. Daskalakis, T. Geralis, V. A. Giakoumopoulou, A. Kyriakis, D. Loukas, A. Markou, C. Markou, A. Psallidas, I. Topsis-Giotis, A. Agapitos, S. Kesisoglou, A. Panagiotou, N. Saoulidou, E. Stiliaris, E. Tziaferi, X. Aslanoglou, I. Evangelou, G. Flouris, C. Foudas, P. Kokkas, N. Manthos, I. Papadopoulos, E. Paradas, J. Strologas, G. Bencze, C. Hajdu, P. Hidas, D. Horvath, F. Sikler, V. Veszpremi, G. Vesztergombi, A. J. Zsigmond, N. Beni, S. Czellar, J. Karancsi, J. Molnar, J. Palinkas, Z. Szillasi, A. Makovec, P. Raics, Z. L. Trocsanyi, B. Ujvari, S. K. Swain, S. B. Beri, V. Bhatnagar, R. Gupta, U. Bhawandeep, A. K. Kalsi, M. Kaur, R. Kumar, M. Mittal, N. Nishu, J. B. Singh, Ashok Kumar, Arun Kumar, S. Ahuja, A. Bhardwaj, B. C. Choudhary, A. Kumar, S. Malhotra, M. Naimuddin, K. Ranjan, V. Sharma, S. Banerjee, S. Bhattacharya, K. Chatterjee, S. Dutta, B. Gomber, Sa. Jain, Sh. Jain, R. Khurana, A. Modak, S. Mukherjee, D. Roy, S. Sarkar, M. Sharan, A. Abdulsalam, D. Dutta, V. Kumar, A. K. Mohanty, L. M. Pant, P. Shukla, A. Topkar, T. Aziz, S. Banerjee, S. Bhowmik, R. M. Chatterjee, R. K. Dewanjee, S. Dugad, S. Ganguly, S. Ghosh, M. Guchait, A. Gurtu, G. Kole, S. Kumar, M. Maity, G. Majumder, K. Mazumdar, G. B. Mohanty, B. Parida, K. Sudhakar, N. Wickramage, S. Sharma, H. Bakhshiansohi, H. Behnamian, S. M. Etesami, A. Fahim, R. Goldouzian, M. Khakzad, M. Mohammadi Najafabadi, M. Naseri, S. Paktinat Mehdiabadi, F. Rezaei Hosseinabadi, B. Safarzadeh, M. Zeinali, M. Felcini, M. Grunewald, M. Abbrescia, C. Calabria, S. S. Chhibra, A. Colaleo, D. Creanza, L. Cristella, N. De Filippis, M. De Palma, L. Fiore, G. Iaselli, G. Maggi, M. Maggi, S. My, S. Nuzzo, A. Pompili, G. Pugliese, R. Radogna, G. Selvaggi, A. Sharma, L. Silvestris, R. Venditti, P. Verwilligen, G. Abbiendi, A. C. Benvenuti, D. Bonacorsi, S. Braibant-Giacomelli, L. Brigliadori, R. Campanini, P. Capiluppi, A. Castro, F. R. Cavallo, G. Codispoti, M. Cuffiani, G. M. Dallavalle, F. Fabbri, A. Fanfani, D. Fasanella, P. Giacomelli, C. Grandi, L. Guiducci, S. Marcellini, G. Masetti, A. Montanari, F. L. Navarria, A. Perrotta, A. M. Rossi, T. Rovelli, G. P. Siroli, N. Tosi, R. Travaglini, S. Albergo, G. Cappello, M. Chiorboli, S. Costa, F. Giordano, R. Potenza, A. Tricomi, C. Tuve, G. Barbagli, V. Ciulli, C. Civinini, R. D’Alessandro, E. Focardi, E. Gallo, S. Gonzi, V. Gori, P. Lenzi, M. Meschini, S. Paoletti, G. Sguazzoni, A. Tropiano, L. Benussi, S. Bianco, F. Fabbri, D. Piccolo, R. Ferretti, F. Ferro, M. Lo Vetere, E. Robutti, S. Tosi, M. E. Dinardo, S. Fiorendi, S. Gennai, R. Gerosa, A. Ghezzi, P. Govoni, M. T. Lucchini, S. Malvezzi, R. A. Manzoni, A. Martelli, B. Marzocchi, D. Menasce, L. Moroni, M. Paganoni, D. Pedrini, S. Ragazzi, N. Redaelli, T. Tabarelli de Fatis, S. Buontempo, N. Cavallo, S. Di Guida, F. Fabozzi, A. O. M. Iorio, L. Lista, S. Meola, M. Merola, P. Paolucci, P. Azzi, N. Bacchetta, D. Biselloa, A. Branca, R. Carlin, P. Checchia, M. Dall’Osso, T. Dorigo, U. Dosselli, F. Gasparini, U. Gasparini, A. Gozzelino, K. Kanishchev, S. Lacaprara, M. Margoni, A. T. Meneguzzo, J. Pazzini, N. Pozzobon, P. Ronchese, F. Simonetto, E. Torassa, M. Tosi, P. Zotto, A. Zucchetta, G. Zumerle, M. Gabusi, S. P. Ratti, V. Re, C. Riccardi, P. Salvini, P. Vitulo, M. Biasini, G. M. Bilei, D. Ciangottini, L. Fanò, P. Lariccia, G. Mantovani, M. Menichelli, A. Saha, A. Santocchia, A. Spiezia, K. Androsov, P. Azzurri, G. Bagliesi, J. Bernardini, T. Boccali, G. Broccolo, R. Castaldi, M. A. Ciocci, R. Dell’Orso, S. Donato, G. Fedi, F. Fiori, L. Foà, A. Giassi, M. T. Grippo, F. Ligabue, T. Lomtadze, L. Martini, A. Messineo, C. S. Moon, F. Palla, A. Rizzi, A. Savoy-Navarro, A. T. Serban, P. Spagnolo, P. Squillacioti, R. Tenchini, G. Tonelli, A. Venturi, P. G. Verdini, C. Vernieri, L. Barone, F. Cavallari, G. D’imperio, D. Del Re, M. Diemoz, C. Jorda, E. Longo, F. Margaroli, P. Meridiani, F. Micheli, G. Organtini, R. Paramatti, S. Rahatlou, C. Rovelli, F. Santanastasio, L. Soffi, P. Traczyk, N. Amapane, R. Arcidiacono, S. Argiro, M. Arneodo, R. Bellan, C. Biino, N. Cartiglia, S. Casasso, M. Costa, R. Covarelli, A. Degano, N. Demaria, L. Finco, C. Mariotti, S. Maselli, E. Migliore, V. Monaco, M. Musich, M. M. Obertino, L. Pacher, N. Pastrone, M. Pelliccioni, G. L. Pinna Angioni, A. Potenza, A. Romero, M. Ruspa, R. Sacchi, A. Solano, A. Staiano, U. Tamponi, S. Belforte, V. Candelise, M. Casarsa, F. Cossutti, G. Della Ricca, B. Gobbo, C. La Licata, M. Marone, A. Schizzi, T. Umer, A. Zanetti, S. Chang, T. A. Kropivnitskaya, S. K. Nam, D. H. Kim, G. N. Kim, M. S. Kim, M. S. Kim, D. J. Kong, S. Lee, Y. D. Oh, H. Park, A. Sakharov, D. C. Son, T. J. Kim, M. S. Ryu, J. Y. Kim, D. H. Moon, S. Song, S. Choi, D. Gyun, B. Hong, M. Jo, H. Kim, Y. Kim, B. Lee, K. S. Lee, S. K. Park, Y. Roh, H. D. Yoo, M. Choi, J. H. Kim, I. C. Park, G. Ryu, Y. Choi, Y. K. Choi, J. Goh, D. Kim, E. Kwon, J. Lee, I. Yu, A. Juodagalvis, J. R. Komaragiri, M. A. B. Md Ali, W. A. T. Wan Abdullah, E. Casimiro Linares, H. Castilla-Valdez, E. De La Cruz-Burelo, I. Heredia-de La Cruz, A. Hernandez-Almada, R. Lopez-Fernandez, A. Sanchez-Hernandez, S. Carrillo Moreno, F. Vazquez Valencia, I. Pedraza, H. A. Salazar Ibarguen, A. Morelos Pineda, D. Krofcheck, P. H. Butler, S. Reucroft, A. Ahmad, M. Ahmad, Q. Hassan, H. R. Hoorani, W. A. Khan, T. Khurshid, M. Shoaib, H. Bialkowska, M. Bluj, B. Boimska, T. Frueboes, M. Górski, M. Kazana, K. Nawrocki, K. Romanowska-Rybinska, M. Szleper, P. Zalewski, G. Brona, K. Bunkowski, M. Cwiok, W. Dominik, K. Doroba, A. Kalinowski, M. Konecki, J. Krolikowski, M. Misiura, M. Olszewski, P. Bargassa, C. Beirão Da Cruz E Silva, P. Faccioli, P. G. Ferreira Parracho, M. Gallinaro, L. Lloret Iglesias, F. Nguyen, J. Rodrigues Antunes, J. Seixas, J. Varela, P. Vischia, S. Afanasiev, P. Bunin, M. Gavrilenko, I. Golutvin, I. Gorbunov, A. Kamenev, V. Karjavin, V. Konoplyanikov, A. Lanev, A. Malakhov, V. Matveev, P. Moisenz, V. Palichik, V. Perelygin, S. Shmatov, N. Skatchkov, V. Smirnov, A. Zarubin, V. Golovtsov, Y. Ivanov, V. Kim, E. Kuznetsova, P. Levchenko, V. Murzin, V. Oreshkin, I. Smirnov, V. Sulimov, L. Uvarov, S. Vavilov, A. Vorobyev, An. Vorobyev, Yu. Andreev, A. Dermenev, S. Gninenko, N. Golubev, M. Kirsanov, N. Krasnikov, A. Pashenkov, D. Tlisov, A. Toropin, V. Epshteyn, V. Gavrilov, N. Lychkovskaya, V. Popov, I. Pozdnyakov, G. Safronov, S. Semenov, A. Spiridonov, V. Stolin, E. Vlasov, A. Zhokin, V. Andreev, M. Azarkin, I. Dremin, M. Kirakosyan, A. Leonidov, G. Mesyats, S. V. Rusakov, A. Vinogradov, A. Belyaev, E. Boos, V. Bunichev, M. Dubinin, L. Dudko, A. Ershov, A. Gribushin, V. Klyukhin, O. Kodolova, I. Lokhtin, S. Obraztsov, S. Petrushanko, V. Savrin, I. Azhgirey, I. Bayshev, S. Bitioukov, V. Kachanov, A. Kalinin, D. Konstantinov, V. Krychkine, V. Petrov, R. Ryutin, A. Sobol, L. Tourtchanovitch, S. Troshin, N. Tyurin, A. Uzunian, A. Volkov, P. Adzic, M. Ekmedzic, J. Milosevic, V. Rekovic, J. Alcaraz Maestre, C. Battilana, E. Calvo, M. Cerrada, M. Chamizo Llatas, N. Colino, B. De La Cruz, A. Delgado Peris, D. Domínguez Vázquez, A. Escalante Del Valle, C. Fernandez Bedoya, J. P. Fernández Ramos, J. Flix, M. C. Fouz, P. Garcia-Abia, O. Gonzalez Lopez, S. Goy Lopez, J. M. Hernandez, M. I. Josa, E. Navarro De Martino, A. Pérez-Calero Yzquierdo, J. Puerta Pelayo, A. Quintario Olmeda, I. Redondo, L. Romero, M. S. Soares, C. Albajar, J. F. de Trocóniz, M. Missiroli, D. Moran, H. Brun, J. Cuevas, J. Fernandez Menendez, S. Folgueras, I. Gonzalez Caballero, J. A. Brochero Cifuentes, I. J. Cabrillo, A. Calderon, J. Duarte Campderros, M. Fernandez, G. Gomez, A. Graziano, A. Lopez Virto, J. Marco, R. Marco, C. Martinez Rivero, F. Matorras, F. J. Munoz Sanchez, J. Piedra Gomez, T. Rodrigo, A. Y. Rodríguez-Marrero, A. Ruiz-Jimeno, L. Scodellaro, I. Vila, R. Vilar Cortabitarte, D. Abbaneo, E. Auffray, G. Auzinger, M. Bachtis, P. Baillon, A. H. Ball, D. Barney, A. Benaglia, J. Bendavid, L. Benhabib, J. F. Benitez, P. Bloch, A. Bocci, A. Bonato, O. Bondu, C. Botta, H. Breuker, T. Camporesi, G. Cerminara, S. Colafranceschi, M. D’Alfonso, D. d’Enterria, A. Dabrowski, A. David, F. De Guio, A. De Roeck, S. De Visscher, E. Di Marco, M. Dobson, M. Dordevic, B. Dorney, N. Dupont-Sagorin, A. Elliott-Peisert, G. Franzoni, W. Funk, D. Gigi, K. Gill, D. Giordano, M. Girone, F. Glege, R. Guida, S. Gundacker, M. Guthoff, J. Hammer, M. Hansen, P. Harris, J. Hegeman, V. Innocente, P. Janot, K. Kousouris, K. Krajczar, P. Lecoq, C. Lourenço, N. Magini, L. Malgeri, M. Mannelli, J. Marrouche, L. Masetti, F. Meijers, S. Mersi, E. Meschi, F. Moortgat, S. Morovic, M. Mulders, S. Orfanelli, L. Orsini, L. Pape, E. Perez, A. Petrilli, G. Petrucciani, A. Pfeiffer, M. Pimiä, D. Piparo, M. Plagge, A. Racz, G. Rolandi, M. Rovere, H. Sakulin, C. Schäfer, C. Schwick, A. Sharma, P. Siegrist, P. Silva, M. Simon, P. Sphicas, D. Spiga, J. Steggemann, B. Stieger, M. Stoye, Y. Takahashi, D. Treille, A. Tsirou, G. I. Veres, N. Wardle, H. K. Wöhri, H. Wollny, W. D. Zeuner, W. Bertl, K. Deiters, W. Erdmann, R. Horisberger, Q. Ingram, H. C. Kaestli, D. Kotlinski, U. Langenegger, D. Renker, T. Rohe, F. Bachmair, L. Bäni, L. Bianchini, M. A. Buchmann, B. Casal, N. Chanon, G. Dissertori, M. Dittmar, M. Donegà, M. Dünser, P. Eller, C. Grab, D. Hits, J. Hoss, G. Kasieczka, W. Lustermann, B. Mangano, A. C. Marini, M. Marionneau, P. Martinez Ruiz del Arbol, M. Masciovecchio, D. Meister, N. Mohr, P. Musella, C. Nägeli, F. Nessi-Tedaldi, F. Pandolfi, F. Pauss, L. Perrozzi, M. Peruzzi, M. Quittnat, L. Rebane, M. Rossini, A. Starodumov, M. Takahashi, K. Theofilatos, R. Wallny, H. A. Weber, C. Amsler, M. F. Canelli, V. Chiochia, A. De Cosa, A. Hinzmann, T. Hreus, B. Kilminster, C. Lange, J. Ngadiuba, D. Pinna, P. Robmann, F. J. Ronga, S. Taroni, Y. Yang, M. Cardaci, K. H. Chen, C. Ferro, C. M. Kuo, W. Lin, Y. J. Lu, R. Volpe, S. S. Yu, P. Chang, Y. H. Chang, Y. Chao, K. F. Chen, P. H. Chen, C. Dietz, U. Grundler, W.-S. Hou, Y. F. Liu, R.-S. Lu, M. Mi nano Moya, E. Petrakou, J. F. Tsai, Y. M. Tzeng, R. Wilken, B. Asavapibhop, G. Singh, N. Srimanobhas, N. Suwonjandee, A. Adiguzel, M. N. Bakirci, S. Cerci, C. Dozen, I. Dumanoglu, E. Eskut, S. Girgis, G. Gokbulut, Y. Guler, E. Gurpinar, I. Hos, E. E. Kangal, A. Kayis Topaksu, G. Onengut, K. Ozdemir, S. Ozturk, A. Polatoz, D. Sunar Cerci, B. Tali, H. Topakli, M. Vergili, C. Zorbilmez, I. V. Akin, B. Bilin, S. Bilmis, H. Gamsizkan, B. Isildak, G. Karapinar, K. Ocalan, S. Sekmen, U. E. Surat, M. Yalvac, M. Zeyrek, E. A. Albayrak, E. Gülmez, M. Kaya, O. Kaya, T. Yetkin, K. Cankocak, F. I. Vardarlı, L. Levchuk, P. Sorokin, J. J. Brooke, E. Clement, D. Cussans, H. Flacher, J. Goldstein, M. Grimes, G. P. Heath, H. F. Heath, J. Jacob, L. Kreczko, C. Lucas, Z. Meng, D. M. Newbold, S. Paramesvaran, A. Poll, T. Sakuma, S. Seif El Nasr-storey, S. Senkin, V. J. Smith, K. W. Bell, A. Belyaev, C. Brew, R. M. Brown, D. J. A. Cockerill, J. A. Coughlan, K. Harder, S. Harper, E. Olaiya, D. Petyt, C. H. Shepherd-Themistocleous, A. Thea, I. R. Tomalin, T. Williams, W. J. Womersley, S. D. Worm, M. Baber, R. Bainbridge, O. Buchmuller, D. Burton, D. Colling, N. Cripps, P. Dauncey, G. Davies, M. Della Negra, P. Dunne, A. Elwood, W. Ferguson, J. Fulcher, D. Futyan, G. Hall, G. Iles, M. Jarvis, G. Karapostoli, M. Kenzie, R. Lane, R. Lucas, L. Lyons, A.-M. Magnan, S. Malik, B. Mathias, J. Nash, A. Nikitenko, J. Pela, M. Pesaresi, K. Petridis, D. M. Raymond, S. Rogerson, A. Rose, C. Seez, P. Sharp, A. Tapper, M. Vazquez Acosta, T. Virdee, S. C. Zenz, J. E. Cole, P. R. Hobson, A. Khan, P. Kyberd, D. Leggat, D. Leslie, I. D. Reid, P. Symonds, L. Teodorescu, M. Turner, J. Dittmann, K. Hatakeyama, A. Kasmi, H. Liu, N. Pastika, T. Scarborough, Z. Wu, O. Charaf, S. I. Cooper, C. Henderson, P. Rumerio, A. Avetisyan, T. Bose, C. Fantasia, P. Lawson, C. Richardson, J. Rohlf, J. St. John, L. Sulak, J. Alimena, E. Berry, S. Bhattacharya, G. Christopher, D. Cutts, Z. Demiragli, N. Dhingra, A. Ferapontov, A. Garabedian, U. Heintz, E. Laird, G. Landsberg, Z. Mao, M. Narain, S. Sagir, T. Sinthuprasith, T. Speer, J. Swanson, R. Breedon, G. Breto, M. Calderon De La Barca Sanchez, S. Chauhan, M. Chertok, J. Conway, R. Conway, P. T. Cox, R. Erbacher, M. Gardner, W. Ko, R. Lander, M. Mulhearn, D. Pellett, J. Pilot, F. Ricci-Tam, S. Shalhout, J. Smith, M. Squires, D. Stolp, M. Tripathi, S. Wilbur, R. Yohay, R. Cousins, P. Everaerts, C. Farrell, J. Hauser, M. Ignatenko, G. Rakness, E. Takasugi, V. Valuev, M. Weber, K. Burt, R. Clare, J. Ellison, J. W. Gary, G. Hanson, J. Heilman, M. Ivova Rikova, P. Jandir, E. Kennedy, F. Lacroix, O. R. Long, A. Luthra, M. Malberti, M. Olmedo Negrete, A. Shrinivas, S. Sumowidagdo, S. Wimpenny, J. G. Branson, G. B. Cerati, S. Cittolin, R. T. D’Agnolo, A. Holzner, R. Kelley, D. Klein, J. Letts, I. Macneill, D. Olivito, S. Padhi, C. Palmer, M. Pieri, M. Sani, V. Sharma, S. Simon, M. Tadel, Y. Tu, A. Vartak, C. Welke, F. Würthwein, A. Yagil, G. Zevi Della Porta, D. Barge, J. Bradmiller-Feld, C. Campagnari, T. Danielson, A. Dishaw, V. Dutta, K. Flowers, M. Franco Sevilla, P. Geffert, C. George, F. Golf, L. Gouskos, J. Incandela, C. Justus, N. Mccoll, S. D. Mullin, J. Richman, D. Stuart, W. To, C. West, J. Yoo, A. Apresyan, A. Bornheim, J. Bunn, Y. Chen, J. Duarte, A. Mott, H. B. Newman, C. Pena, M. Pierini, M. Spiropulu, R. Vlimant, R. Wilkinson, S. Xie, R. Y. Zhu, V. Azzolini, A. Calamba, B. Carlson, T. Ferguson, Y. Iiyama, M. Paulini, J. Russ, H. Vogel, I. Vorobiev, J. P. Cumalat, W. T. Ford, A. Gaz, M. Krohn, E. Luiggi Lopez, U. Nauenberg, J. G. Smith, K. Stenson, S. R. Wagner, J. Alexander, A. Chatterjee, J. Chaves, J. Chu, S. Dittmer, N. Eggert, N. Mirman, G. Nicolas Kaufman, J. R. Patterson, A. Ryd, E. Salvati, L. Skinnari, W. Sun, W. D. Teo, J. Thom, J. Thompson, J. Tucker, Y. Weng, L. Winstrom, P. Wittich, D. Winn, S. Abdullin, M. Albrow, J. Anderson, G. Apollinari, L. A. T. Bauerdick, A. Beretvas, J. Berryhill, P. C. Bhat, G. Bolla, K. Burkett, J. N. Butler, H. W. K. Cheung, F. Chlebana, S. Cihangir, V. D. Elvira, I. Fisk, J. Freeman, E. Gottschalk, L. Gray, D. Green, S. Grünendahl, O. Gutsche, J. Hanlon, D. Hare, R. M. Harris, J. Hirschauer, B. Hooberman, S. Jindariani, M. Johnson, U. Joshi, B. Klima, B. Kreis, S. Kwan, J. Linacre, D. Lincoln, R. Lipton, T. Liu, R. Lopes De Sá, J. Lykken, K. Maeshima, J. M. Marraffino, V. I. Martinez Outschoorn, S. Maruyama, D. Mason, P. McBride, P. Merkel, K. Mishra, S. Mrenna, S. Nahn, C. Newman-Holmes, V. O’Dell, O. Prokofyev, E. Sexton-Kennedy, A. Soha, W. J. Spalding, L. Spiegel, L. Taylor, S. Tkaczyk, N. V. Tran, L. Uplegger, E. W. Vaandering, R. Vidal, A. Whitbeck, J. Whitmore, F. Yang, D. Acosta, P. Avery, P. Bortignon, D. Bourilkov, M. Carver, D. Curry, S. Das, M. De Gruttola, G. P. Di Giovanni, R. D. Field, M. Fisher, I. K. Furic, J. Hugon, J. Konigsberg, A. Korytov, T. Kypreos, J. F. Low, K. Matchev, H. Mei, P. Milenovic, G. Mitselmakher, L. Muniz, A. Rinkevicius, L. Shchutska, M. Snowball, D. Sperka, J. Yelton, M. Zakaria, S. Hewamanage, S. Linn, P. Markowitz, G. Martinez, J. L. Rodriguez, J. R. Adams, T. Adams, A. Askew, J. Bochenek, B. Diamond, J. Haas, S. Hagopian, V. Hagopian, K. F. Johnson, H. Prosper, V. Veeraraghavan, M. Weinberg, M. M. Baarmand, M. Hohlmann, H. Kalakhety, F. Yumiceva, M. R. Adams, L. Apanasevich, D. Berry, R. R. Betts, I. Bucinskaite, R. Cavanaugh, O. Evdokimov, L. Gauthier, C. E. Gerber, D. J. Hofman, P. Kurt, C. O’Brien, I. D. Sandoval Gonzalez, C. Silkworth, P. Turner, N. Varelas, B. Bilki, W. Clarida, K. Dilsiz, M. Haytmyradov, V. Khristenko, J.-P. Merlo, H. Mermerkaya, A. Mestvirishvili, A. Moeller, J. Nachtman, H. Ogul, Y. Onel, F. Ozok, A. Penzo, R. Rahmat, S. Sen, P. Tan, E. Tiras, J. Wetzel, K. Yi, I. Anderson, B. A. Barnett, B. Blumenfeld, S. Bolognesi, D. Fehling, A. V. Gritsan, P. Maksimovic, C. Martin, M. Swartz, M. Xiao, P. Baringer, A. Bean, G. Benelli, C. Bruner, J. Gray, R. P. Kenny, D. Majumder, M. Malek, M. Murray, D. Noonan, S. Sanders, J. Sekaric, R. Stringer, Q. Wang, J. S. Wood, I. Chakaberia, A. Ivanov, K. Kaadze, S. Khalil, M. Makouski, Y. Maravin, L. K. Saini, N. Skhirtladze, I. Svintradze, J. Gronberg, D. Lange, F. Rebassoo, D. Wright, A. Baden, A. Belloni, B. Calvert, S. C. Eno, J. A. Gomez, N. J. Hadley, S. Jabeen, R. G. Kellogg, T. Kolberg, Y. Lu, A. C. Mignerey, K. Pedro, A. Skuja, M. B. Tonjes, S. C. Tonwar, A. Apyan, R. Barbieri, K. Bierwagen, W. Busza, I. A. Cali, L. Di Matteo, G. Gomez Ceballos, M. Goncharov, D. Gulhan, M. Klute, Y. S. Lai, Y.-J. Lee, A. Levin, P. D. Luckey, C. Paus, D. Ralph, C. Roland, G. Roland, G. S. F. Stephans, K. Sumorok, D. Velicanu, J. Veverka, B. Wyslouch, M. Yang, M. Zanetti, V. Zhukova, B. Dahmes, A. Gude, S. C. Kao, K. Klapoetke, Y. Kubota, J. Mans, S. Nourbakhsh, R. Rusack, A. Singovsky, N. Tambe, J. Turkewitz, J. G. Acosta, S. Oliveros, E. Avdeeva, K. Bloom, S. Bose, D. R. Claes, A. Dominguez, R. Gonzalez Suarez, J. Keller, D. Knowlton, I. Kravchenko, J. Lazo-Flores, F. Meier, F. Ratnikov, G. R. Snow, M. Zvada, J. Dolen, A. Godshalk, I. Iashvili, A. Kharchilava, A. Kumar, S. Rappoccio, G. Alverson, E. Barberis, D. Baumgartel, M. Chasco, A. Massironi, D. M. Morse, D. Nash, T. Orimoto, D. Trocino, R. J. Wang, D. Wood, J. Zhang, K. A. Hahn, A. Kubik, N. Mucia, N. Odell, B. Pollack, A. Pozdnyakov, M. Schmitt, S. Stoynev, K. Sung, M. Velasco, S. Won, A. Brinkerhoff, K. M. Chan, A. Drozdetskiy, M. Hildreth, C. Jessop, D. J. Karmgard, N. Kellams, K. Lannon, S. Lynch, N. Marinelli, Y. Musienko, T. Pearson, M. Planer, R. Ruchti, G. Smith, N. Valls, M. Wayne, M. Wolf, A. Woodard, L. Antonelli, J. Brinson, B. Bylsma, L. S. Durkin, S. Flowers, A. Hart, C. Hill, R. Hughes, K. Kotov, T. Y. Ling, W. Luo, D. Puigh, M. Rodenburg, B. L. Winer, H. Wolfe, H. W. Wulsin, O. Driga, P. Elmer, J. Hardenbrook, P. Hebda, S. A. Koay, P. Lujan, D. Marlow, T. Medvedeva, M. Mooney, J. Olsen, P. Piroué, X. Quan, H. Saka, D. Stickland, C. Tully, J. S. Werner, A. Zuranski, E. Brownson, S. Malik, H. Mendez, J. E. Ramirez Vargas, V. E. Barnes, D. Benedetti, D. Bortoletto, L. Gutay, Z. Hu, M. K. Jha, M. Jones, K. Jung, M. Kress, N. Leonardo, D. H. Miller, N. Neumeister, F. Primavera, B. C. Radburn-Smith, X. Shi, I. Shipsey, D. Silvers, A. Svyatkovskiy, F. Wang, W. Xie, L. Xu, J. Zablocki, N. Parashar, J. Stupak, A. Adair, B. Akgun, K. M. Ecklund, F. J. M. Geurts, W. Li, B. Michlin, B. P. Padley, R. Redjimi, J. Roberts, J. Zabel, B. Betchart, A. Bodek, P. de Barbaro, R. Demina, Y. Eshaq, T. Ferbel, M. Galanti, A. Garcia-Bellido, P. Goldenzweig, J. Han, A. Harel, O. Hindrichs, A. Khukhunaishvili, S. Korjenevski, G. Petrillo, M. Verzetti, D. Vishnevskiy, R. Ciesielski, L. Demortier, K. Goulianos, C. Mesropian, S. Arora, A. Barker, J. P. Chou, C. Contreras-Campana, E. Contreras-Campana, D. Duggan, D. Ferencek, Y. Gershtein, R. Gray, E. Halkiadakis, D. Hidas, S. Kaplan, A. Lath, S. Panwalkar, M. Park, S. Salur, S. Schnetzer, D. Sheffield, S. Somalwar, R. Stone, S. Thomas, P. Thomassen, M. Walker, K. Rose, S. Spanier, A. York, O. Bouhali, A. Castaneda Hernandez, M. Dalchenko, M. De Mattia, S. Dildick, R. Eusebi, W. Flanagan, J. Gilmore, T. Kamon, V. Khotilovich, V. Krutelyov, R. Montalvo, I. Osipenkov, Y. Pakhotin, R. Patel, A. Perloff, J. Roe, A. Rose, A. Safonov, I. Suarez, A. Tatarinov, K. A. Ulmer, N. Akchurin, C. Cowden, J. Damgov, C. Dragoiu, P. R. Dudero, J. Faulkner, K. Kovitanggoon, S. Kunori, S. W. Lee, T. Libeiro, I. Volobouev, E. Appelt, A. G. Delannoy, S. Greene, A. Gurrola, W. Johns, C. Maguire, Y. Mao, A. Melo, M. Sharma, P. Sheldon, B. Snook, S. Tuo, J. Velkovska, M. W. Arenton, S. Boutle, B. Cox, B. Francis, J. Goodell, R. Hirosky, A. Ledovskoy, H. Li, C. Lin, C. Neu, E. Wolfe, J. Wood, C. Clarke, R. Harr, P. E. Karchin, C. Kottachchi Kankanamge Don, P. Lamichhane, J. Sturdy, D. A. Belknap, D. Carlsmith, M. Cepeda, S. Dasu, L. Dodd, S. Duric, E. Friis, R. Hall-Wilton, M. Herndon, A. Hervé, P. Klabbers, A. Lanaro, C. Lazaridis, A. Levine, R. Loveless, A. Mohapatra, I. Ojalvo, T. Perry, G. A. Pierro, G. Polese, I. Ross, T. Sarangi, A. Savin, W. H. Smith, D. Taylor, C. Vuosalo, N. Woods, V. Roinishvili, The CMS Collaboration

**Affiliations:** Yerevan Physics Institute, Yerevan, Armenia; Institut für Hochenergiephysik der OeAW, Wien, Austria; National Centre for Particle and High Energy Physics, Minsk, Belarus; Universiteit Antwerpen, Antwerpen, Belgium; Vrije Universiteit Brussel, Brussel, Belgium; Université Libre de Bruxelles, Brussels, Belgium; Ghent University, Ghent, Belgium; Université Catholique de Louvain, Louvain-la-Neuve, Belgium; Université de Mons, Mons, Belgium; Centro Brasileiro de Pesquisas Fisicas, Rio de Janeiro, Brazil; Universidade do Estado do Rio de Janeiro, Rio de Janeiro, Brazil; Universidade Estadual Paulista, Universidade Federal do ABC, São Paulo, Brazil; Institute for Nuclear Research and Nuclear Energy, Sofia, Bulgaria; University of Sofia, Sofia, Bulgaria; Institute of High Energy Physics, Beijing, China; State Key Laboratory of Nuclear Physics and Technology, Peking University, Beijing, China; Universidad de Los Andes, Bogotá, Colombia; Faculty of Electrical Engineering, Mechanical Engineering and Naval Architecture, University of Split, Split, Croatia; Faculty of Science, University of Split, Split, Croatia; Institute Rudjer Boskovic, Zagreb, Croatia; University of Cyprus, Nicosia, Cyprus; Charles University, Prague, Czech Republic; Academy of Scientific Research and Technology of the Arab Republic of Egypt, Egyptian Network of High Energy Physics, Cairo, Egypt; National Institute of Chemical Physics and Biophysics, Tallinn, Estonia; Department of Physics, University of Helsinki, Helsinki, Finland; Helsinki Institute of Physics, Helsinki, Finland; Lappeenranta University of Technology, Lappeenranta, Finland; DSM/IRFU, CEA/Saclay, Gif-sur-Yvette, France; Laboratoire Leprince-Ringuet, Ecole Polytechnique, IN2P3-CNRS, Palaiseau, France; Institut Pluridisciplinaire Hubert Curien, Université de Strasbourg, Université de Haute Alsace Mulhouse, CNRS/IN2P3, Strasbourg, France; Centre de Calcul de l’Institut National de Physique Nucleaire et de Physique des Particules, CNRS/IN2P3, Villeurbanne, France; Institut de Physique Nucléaire de Lyon, Université de Lyon, Université Claude Bernard Lyon 1, CNRS-IN2P3, Villeurbanne, France; Institute of High Energy Physics and Informatization, Tbilisi State University, Tbilisi, Georgia; I. Physikalisches Institut, RWTH Aachen University, Aachen, Germany; III. Physikalisches Institut A, RWTH Aachen University, Aachen, Germany; III. Physikalisches Institut B, RWTH Aachen University, Aachen, Germany; Deutsches Elektronen-Synchrotron, Hamburg, Germany; University of Hamburg, Hamburg, Germany; Institut für Experimentelle Kernphysik, Karlsruhe, Germany; Institute of Nuclear and Particle Physics (INPP), NCSR Demokritos, Aghia Paraskevi, Greece; University of Athens, Athens, Greece; University of Ioánnina, Ioannina, Greece; Wigner Research Centre for Physics, Budapest, Hungary; Institute of Nuclear Research ATOMKI, Debrecen, Hungary; University of Debrecen, Debrecen, Hungary; National Institute of Science Education and Research, Bhubaneswar, India; Panjab University, Chandigarh, India; University of Delhi, Delhi, India; Saha Institute of Nuclear Physics, Kolkata, India; Bhabha Atomic Research Centre, Mumbai, India; Tata Institute of Fundamental Research, Mumbai, India; Indian Institute of Science Education and Research (IISER), Pune, India; Institute for Research in Fundamental Sciences (IPM), Tehran, Iran; University College Dublin, Dublin, Ireland; INFN Sezione di Bari, Università di Bari, Politecnico di Bari, Bari, Italy; INFN Sezione di Bologna, Università di Bologna, Bologna, Italy; INFN Sezione di Catania, Università di Catania, CSFNSM, Catania, Italy; INFN Sezione di Firenze, Università di Firenze, Florence, Italy; INFN Laboratori Nazionali di Frascati, Frascati, Italy; INFN Sezione di Genova, Università di Genova, Genoa, Italy; INFN Sezione di Milano-Bicocca, Università di Milano-Bicocca, Milan, Italy; INFN Sezione di Napoli, Università di Napoli ’Federico II’, Università della Basilicata (Potenza), Università G. Marconi (Roma), Naples, Italy; INFN Sezione di Padova, Università di Padova, Università di Trento (Trento), Padua, Italy; INFN Sezione di Pavia, Università di Pavia, Pavia, Italy; INFN Sezione di Perugia, Università di Perugia, Perugia, Italy; INFN Sezione di Pisa, Università di Pisa, Scuola Normale Superiore di Pisa, Pisa, Italy; INFN Sezione di Roma, Università di Roma, Rome, Italy; INFN Sezione di Torino, Università di Torino, Università del Piemonte Orientale (Novara), Torin, Italy; INFN Sezione di Trieste, Università di Trieste, Trieste, Italy; Kangwon National University, Chunchon, Korea; Kyungpook National University, Taegu, Korea; Chonbuk National University, Chonju, Korea; Chonnam National University, Institute for Universe and Elementary Particles, Kwangju, Korea; Korea University, Seoul, Korea; Seoul National University, Seoul, Korea; University of Seoul, Seoul, Korea; Sungkyunkwan University, Suwon, Korea; Vilnius University, Vilnius, Lithuania; National Centre for Particle Physics, Universiti Malaya, Kuala Lumpur, Malaysia; Centro de Investigacion y de Estudios Avanzados del IPN, Mexico City, Mexico; Universidad Iberoamericana, Mexico City, Mexico; Benemerita Universidad Autonoma de Puebla, Puebla, Mexico; Universidad Autónoma de San Luis Potosí, San Luis Potosí, Mexico; University of Auckland, Auckland, New Zealand; University of Canterbury, Christchurch, New Zealand; National Centre for Physics, Quaid-I-Azam University, Islamabad, Pakistan; National Centre for Nuclear Research, Swierk, Poland; Institute of Experimental Physics, Faculty of Physics, University of Warsaw, Warsaw, Poland; Laboratório de Instrumentação e Física Experimental de Partículas, Lisbon, Portugal; Joint Institute for Nuclear Research, Dubna, Russia; Petersburg Nuclear Physics Institute, Gatchina (St. Petersburg), Russia; Institute for Nuclear Research, Moscow, Russia; Institute for Theoretical and Experimental Physics, Moscow, Russia; P. N. Lebedev Physical Institute, Moscow, Russia; Skobeltsyn Institute of Nuclear Physics, Lomonosov Moscow State University, Moscow, Russia; State Research Center of Russian Federation, Institute for High Energy Physics, Protvino, Russia; Faculty of Physics and Vinca Institute of Nuclear Sciences, University of Belgrade, Belgrade, Serbia; Centro de Investigaciones Energéticas Medioambientales y Tecnológicas (CIEMAT), Madrid, Spain; Universidad Autónoma de Madrid, Madrid, Spain; Universidad de Oviedo, Oviedo, Spain; Instituto de Física de Cantabria (IFCA), CSIC-Universidad de Cantabria, Santander, Spain; CERN, European Organization for Nuclear Research, Geneva, Switzerland; Paul Scherrer Institut, Villigen, Switzerland; Institute for Particle Physics, ETH Zurich, Zurich, Switzerland; Universität Zürich, Zurich, Switzerland; National Central University, Chung-Li, Taiwan; National Taiwan University (NTU), Taipei, Taiwan; Department of Physics, Faculty of Science, Chulalongkorn University, Bangkok, Thailand; Cukurova University, Adana, Turkey; Physics Department, Middle East Technical University, Ankara, Turkey; Bogazici University, Istanbul, Turkey; Istanbul Technical University, Istanbul, Turkey; National Scientific Center, Kharkov Institute of Physics and Technology, Kharkiv, Ukraine; University of Bristol, Bristol, UK; Rutherford Appleton Laboratory, Didcot, UK; Imperial College, London, UK; Brunel University, Uxbridge, UK; Baylor University, Waco, USA; The University of Alabama, Tuscaloosa, USA; Boston University, Boston, USA; Brown University, Providence, USA; University of California, Davis, USA; University of California, Los Angeles, USA; University of California, Riverside, Riverside, USA; University of California, San Diego, La Jolla, USA; University of California, Santa Barbara, Santa Barbara, USA; California Institute of Technology, Pasadena, USA; Carnegie Mellon University, Pittsburgh, USA; University of Colorado at Boulder, Boulder, USA; Cornell University, Ithaca, USA; Fairfield University, Fairfield, USA; Fermi National Accelerator Laboratory, Batavia, USA; University of Florida, Gainesville, USA; Florida International University, Miami, USA; Florida State University, Tallahassee, USA; Florida Institute of Technology, Melbourne, USA; University of Illinois at Chicago (UIC), Chicago, USA; The University of Iowa, Iowa City, USA; Johns Hopkins University, Baltimore, USA; The University of Kansas, Lawrence, USA; Kansas State University, Manhattan, USA; Lawrence Livermore National Laboratory, Livermore, USA; University of Maryland, College Park, USA; Massachusetts Institute of Technology, Cambridge, USA; University of Minnesota, Minneapolis, USA; University of Mississippi, Oxford, USA; University of Nebraska-Lincoln, Lincoln, USA; State University of New York at Buffalo, Buffalo, USA; Northeastern University, Boston, USA; Northwestern University, Evanston, USA; University of Notre Dame, Notre Dame, USA; The Ohio State University, Columbus, USA; Princeton University, Princeton, USA; University of Puerto Rico, Mayagüez, USA; Purdue University, West Lafayette, USA; Purdue University Calumet, Hammond, USA; Rice University, Houston, USA; University of Rochester, Rochester, USA; The Rockefeller University, New York, USA; Rutgers, The State University of New Jersey, Piscataway, USA; University of Tennessee, Knoxville, USA; Texas A&M University, College Station, USA; Texas Tech University, Lubbock, USA; Vanderbilt University, Nashville, USA; University of Virginia, Charlottesville, USA; Wayne State University, Detroit, USA; University of Wisconsin, Madison, USA; CERN, Geneva, Switzerland; E. Andronikashvili Institute of Physics, Academy of Science, Tbilisi, Georgia

## Abstract

Properties of the Higgs boson with mass near 125$\,\text {GeV}$ are measured in proton-proton collisions with the CMS experiment at the LHC. Comprehensive sets of production and decay measurements are combined. The decay channels include $\gamma \gamma$, $\mathrm{Z}\mathrm{Z}$, $\mathrm{W}\mathrm{W}$, $\tau \tau$, $\mathrm{b} \mathrm{b}$, and $\mu \mu$ pairs. The data samples were collected in 2011 and 2012 and correspond to integrated luminosities of up to 5.1$\,\text {fb}^\text {-1}$ at 7$\,\text {TeV}$ and up to 19.7$\,\text {fb}^\text {-1}$ at 8$\,\text {TeV}$. From the high-resolution $\gamma \gamma$ and $\mathrm{Z}\mathrm{Z}$ channels, the mass of the Higgs boson is determined to be $125.02\,^{+0.26}_{-0.27} \,\text {(stat)} \,^{+0.14}_{-0.15} \,\text {(syst)} \,\text {GeV}$. For this mass value, the event yields obtained in the different analyses tagging specific decay channels and production mechanisms are consistent with those expected for the standard model Higgs boson. The combined best-fit signal relative to the standard model expectation is $1.00\,\pm 0.09\,\text {(stat)} \,^{+0.08}_{-0.07}\,\text {(theo)} \,\pm 0.07\,\text {(syst)}$ at the measured mass. The couplings of the Higgs boson are probed for deviations in magnitude from the standard model predictions in multiple ways, including searches for invisible and undetected decays. No significant deviations are found.

## Introduction

One of the most important objectives of the physics programme at the CERN LHC is to understand the mechanism behind electroweak symmetry breaking (EWSB). In the standard model (SM) [1–3] EWSB is achieved by a complex scalar doublet field that leads to the prediction of one physical Higgs boson ($\mathrm{H}$) [4–9]. Through Yukawa interactions, the Higgs scalar field can also account for fermion masses [10–12].

In 2012 the ATLAS and CMS Collaborations at the LHC reported the observation of a new boson with mass near 125$\,\text {GeV}$  [13–15], a value confirmed in later measurements [16–18]. Subsequent studies of the production and decay rates [16, 18–38] and of the spin-parity quantum numbers [16, 22, 39–41] of the new boson show that its properties are compatible with those expected for the SM Higgs boson. The CDF and D0 experiments have also reported an excess of events consistent with the LHC observations [42, 43].

Standard model predictions have improved with time, and the results presented in this paper make use of a large number of theory tools and calculations [44–168], summarized in Refs. [169–171]. In proton-proton (pp) collisions at $\sqrt{s}=\text {7--8}\,\text {TeV}$, the gluon-gluon fusion Higgs boson production mode ($\mathrm{g} \mathrm{g} \mathrm{H}$) has the largest cross section. It is followed by vector boson fusion ($\mathrm{VBF}$), associated $\mathrm{W}\mathrm{H}$ and $\mathrm{Z}\mathrm{H}$ production ($\mathrm{V}\mathrm{H}$), and production in association with a top quark pair ($\mathrm{t}\mathrm{t}\mathrm{H}$). The cross section values for the Higgs boson production modes and the values for the decay branching fractions, together with their uncertainties, are tabulated in Ref. [171] and regular online updates. For a Higgs boson mass of 125$\,\text {GeV}$, the total production cross section is expected to be 17.5$\text {\,pb}$ at $\sqrt{s}=7\,\text {TeV}$ and 22.3$\text {\,pb}$ at 8$\,\text {TeV}$, and varies with the mass at a rate of about $-1.6\,\%$ per $\text {GeV}$.

This paper presents results from a comprehensive analysis combining the CMS measurements of the properties of the Higgs boson targeting its decay to $\mathrm{b} \mathrm{b}$  [21], $\mathrm{W}\mathrm{W}$  [22], $\mathrm{Z}\mathrm{Z}$  [16], $\tau \tau$  [23], $\gamma \gamma$  [18], and $\mu \mu$  [30] as well as measurements of the $\mathrm{t}\mathrm{t}\mathrm{H}$ production mode [29] and searches for invisible decays of the Higgs boson [28]. For simplicity, $\mathrm{b} \mathrm{b}$ is used to denote $\mathrm{b}\overline{\mathrm{b}}$, $\tau \tau$ to denote $\mathrm{\tau }^+\mathrm{\tau }^-$, etc. Similarly, $\mathrm{Z}\mathrm{Z}$ is used to denote $\mathrm{Z}\mathrm{Z} ^{(*)}$ and $\mathrm{W}\mathrm{W}$ to denote $\mathrm{W}\mathrm{W} ^{(*)}$. The broad complementarity of measurements targeting different production and decay modes enables a variety of studies of the couplings of the new boson to be performed.

The different analyses have different sensitivities to the presence of the SM Higgs boson. The $\mathrm{H} \rightarrow \gamma \gamma$ and $\mathrm{H} \rightarrow \mathrm{Z}\mathrm{Z} \rightarrow 4\ell$ (where $\ell = \mathrm{e},\mu$) channels play a special role because of their high sensitivity and excellent mass resolution of the reconstructed diphoton and four-lepton final states, respectively. The $\mathrm{H} \rightarrow \mathrm{W}\mathrm{W} \rightarrow \ell \nu \ell \nu$ measurement has a high sensitivity due to large expected yields but relatively poor mass resolution because of the presence of neutrinos in the final state. The $\mathrm{b} \mathrm{b}$ and $\tau \tau$ decay modes are beset by large background contributions and have relatively poor mass resolution, resulting in lower sensitivity compared to the other channels; combining the results from $\mathrm{b} \mathrm{b}$ and $\tau \tau$, the CMS Collaboration has published evidence for the decay of the Higgs boson to fermions [172]. In the SM the $\mathrm{g} \mathrm{g} \mathrm{H}$ process is dominated by a virtual top quark loop. However, the direct coupling of top quarks to the Higgs boson can be probed through the study of events tagged as having been produced via the $\mathrm{t}\mathrm{t}\mathrm{H}$ process.

The mass of the Higgs boson is determined by combining the measurements performed in the $\mathrm{H} \rightarrow \gamma \gamma$ and $\mathrm{H} \rightarrow \mathrm{Z}\mathrm{Z} \rightarrow 4\ell$ channels [16, 18]. The SM Higgs boson is predicted to have even parity, zero electric charge, and zero spin. All its other properties can be derived if the boson’s mass is specified. To investigate the couplings of the Higgs boson to SM particles, we perform a combined analysis of all measurements to extract ratios between the observed coupling strengths and those predicted by the SM.

The couplings of the Higgs boson are probed for deviations in magnitude using the formalism recommended by the LHC Higgs Cross Section Working Group in Ref. [171]. This formalism assumes, among other things, that the observed state has quantum numbers $J^{PC} =0^{++}$ and that the narrow-width approximation holds, leading to a factorization of the couplings in the production and decay of the boson.

The data sets were processed with updated alignment and calibrations of the CMS detector and correspond to integrated luminosities of up to 5.1$\,\text {fb}^\text {-1}$ at $\sqrt{s}=7\,\text {TeV}$ and 19.7$\,\text {fb}^\text {-1}$ at 8$\,\text {TeV}$ for pp collisions collected in 2011 and 2012. The central feature of the CMS detector is a 13$\text {\,m}$ long superconducting solenoid of 6$\text {\,m}$ internal diameter that generates a uniform 3.8$\text {\,T}$ magnetic field parallel to the direction of the LHC beams. Within the solenoid volume are a silicon pixel and strip tracker, a lead tungstate crystal electromagnetic calorimeter, and a brass and scintillator hadron calorimeter. Muons are identified and measured in gas-ionization detectors embedded in the steel magnetic flux-return yoke of the solenoid. The detector is subdivided into a cylindrical barrel and two endcap disks. Calorimeters on either side of the detector complement the coverage provided by the barrel and endcap detectors. A more detailed description of the CMS detector, together with a definition of the coordinate system used and the relevant kinematic variables, can be found in Ref. [173].

This paper is structured as follows: Sect. 2 summarizes the analyses contributing to the combined measurements. Section 3 describes the statistical method used to extract the properties of the boson; some expected differences between the results of the combined analysis and those of the individual analyses are also explained. The results of the combined analysis are reported in the following four sections. A precise determination of the mass of the boson and direct limits on its width are presented in Sect. 4. We then discuss the significance of the observed excesses of events in Sect. 5. Finally, Sects. 6 and 7 present multiple evaluations of the compatibility of the data with the SM expectations for the magnitude of the Higgs boson’s couplings.

## Inputs to the combined analysis

Table 1 provides an overview of all inputs used in this combined analysis, including the following information: the final states selected, the production and decay modes targeted in the analyses, the integrated luminosity used, the expected mass resolution, and the number of event categories in each channel.

Both Table 1 and the descriptions of the different inputs make use of the following notation. The expected relative mass resolution, $\sigma _{m_\mathrm{{H}}}/m_\mathrm{{H}}$, is estimated using different $\sigma _{m_\mathrm{{H}}}$ calculations: the $\mathrm{H} \rightarrow \gamma \gamma$, $\mathrm{H} \rightarrow \mathrm{Z}\mathrm{Z} \rightarrow 4\ell$, $\mathrm{H} \rightarrow \mathrm{W}\mathrm{W} \rightarrow \ell \nu \ell \nu$, and $\mathrm{H} \rightarrow \mu \mu$ analyses quote $\sigma _{m_\mathrm{{H}}}$ as half of the width of the shortest interval containing $68.3\,\%$ of the signal events, the $\mathrm{H} \rightarrow \tau \tau$ analysis quotes the RMS of the signal distribution, and the analysis of $\mathrm{V}\mathrm{H}$ with $\mathrm{H} \rightarrow \mathrm{b} \mathrm{b}$ quotes the standard deviation of the Gaussian core of a function that also describes non-Gaussian tails. Regarding leptons, $\ell$ denotes an electron or a muon, $\mathrm{\tau }_{\mathrm{h}}$ denotes a $\mathrm{\tau }$ lepton identified via its decay into hadrons, and $L$ denotes any charged lepton. Regarding lepton pairs, SF (DF) denotes same-flavour (different-flavour) pairs and SS (OS) denotes same-sign (opposite-sign) pairs. Concerning reconstructed jets, CJV denotes a central jet veto, $p_{\mathrm{T}}$ is the magnitude of the transverse momentum vector, $E_{\mathrm{T}}^{\text {miss}}$ refers to the magnitude of the missing transverse momentum vector, ${\mathrm{j}}$ stands for a reconstructed jet, and $\mathrm{b}$ denotes a jet tagged as originating from the hadronization of a bottom quark.

**Table 1:** Summary of the channels in the analyses included in this combination. The first and second columns indicate which decay mode and production mechanism is targeted by an analysis. Notes on the expected composition of the signal are given in the third column. Where available, the fourth column specifies the expected relative mass resolution for the SM Higgs boson. Finally, the last columns provide the number of event categories and the integrated luminosity for the 7 and 8$\,\text {TeV}$ data sets. The notation is explained in the text

Decay tag and production tag		Expected signal composition	$\sigma _{m_\mathrm{{H}}}/m_\mathrm{{H}}$	*Luminosity *($\,\text {fb}^\text {-1}$ )	
				No. of categories	
				7$\,\text {TeV}$	8$\,\text {TeV}$
$\mathrm{H} \rightarrow \gamma \gamma$ [18], Sect. 2.1				*5.1*	*19.7*
$\gamma \gamma$	Untagged	76–93 % $\mathrm{g} \mathrm{g} \mathrm{H}$	0.8–2.1 %	4	5
	2-jet $\mathrm{VBF}$	50–80 % $\mathrm{VBF}$	1.0–1.3 %	2	3
	Leptonic $\mathrm{V}\mathrm{H}$	${\approx }95\,\%$ $\mathrm{V}\mathrm{H}$ ($\mathrm{W}\mathrm{H} /\mathrm{Z}\mathrm{H} \approx 5$)	1.3 %	2	2
	$E_{\mathrm{T}}^{\text {miss}}$ $\mathrm{V}\mathrm{H}$	70–80 % $\mathrm{V}\mathrm{H}$ ($\mathrm{W}\mathrm{H} /\mathrm{Z}\mathrm{H} \approx 1$)	1.3 %	1	1
	2-jet $\mathrm{V}\mathrm{H}$	${\approx }65\,\%$ $\mathrm{V}\mathrm{H}$ ($\mathrm{W}\mathrm{H} /\mathrm{Z}\mathrm{H} \approx 5$)	1.0–1.3 %	1	1
	Leptonic $\mathrm{t}\mathrm{t}\mathrm{H}$	${\approx }95\,\%$ $\mathrm{t}\mathrm{t}\mathrm{H}$	1.1 %	$1^{\dagger }$	1
	Multijet $\mathrm{t}\mathrm{t}\mathrm{H}$	${>}90\,\%$ $\mathrm{t}\mathrm{t}\mathrm{H}$	1.1 %		1
$\mathrm{H} \rightarrow \mathrm{Z}\mathrm{Z} \rightarrow 4\ell$ [16], Sect. 2.2				*5.1*	*19.7*
$4\mathrm{\mu }$, $2\mathrm{e}2\mathrm{\mu }/2\mathrm{\mu }2\mathrm{e}$, $4\mathrm{e}$	0/1-jet	${\approx }90\,\%$ $\mathrm{g} \mathrm{g} \mathrm{H}$		3	3
	2-jet	$42\,\%$ $(\mathrm{VBF} +\mathrm{V}\mathrm{H} )$	1.3, 1.8, 2.2 %$^{\ddagger }$	3	3
$\mathrm{H} \rightarrow \mathrm{W}\mathrm{W} \rightarrow \ell \nu \ell \nu$ [22], Sect. 2.3				*4.9*	*19.4*
$\mathrm{e}\mathrm{e} +\mu \mu$, $\mathrm{e}\mu$	0-jet	96–98 % $\mathrm{g} \mathrm{g} \mathrm{H}$	16 %$^{\ddagger }$	2	2
	1-jet	82–84 % $\mathrm{g} \mathrm{g} \mathrm{H}$	17 %$^{\ddagger }$	2	2
	2-jet $\mathrm{VBF}$	78–86 % $\mathrm{VBF}$		2	2
	2-jet $\mathrm{V}\mathrm{H}$	31–40 % $\mathrm{V}\mathrm{H}$		2	2
$3\ell 3\nu$ ($\mathrm{W}\mathrm{H}$)	SF-SS, SF-OS	${\approx }100\,\%$ $\mathrm{W}\mathrm{H}$, up to 20 % $\tau \tau$		2	2
$\ell \ell +\ell ^{\prime }\nu {\mathrm{jj}}$ ($\mathrm{Z}\mathrm{H}$)	$\mathrm{e}\mathrm{e} \mathrm{e}$, $\mathrm{e}\mathrm{e} \mu$, $\mu \mu \mu$, $\mu \mu \mathrm{e}$	${\approx }100\,\%$ $\mathrm{Z}\mathrm{H}$		4	4
$\mathrm{H} \rightarrow \tau \tau$ [23], Sect. 2.4				*4.9*	*19.7*
$\mathrm{e}\mathrm{\tau }_{\mathrm{h}}$, $\mathrm{\mu }\mathrm{\tau }_{\mathrm{h}}$	0-jet	${\approx }98\,\%$ $\mathrm{g} \mathrm{g} \mathrm{H}$	11–14 %	4	4
	1-jet	70–80 % $\mathrm{g} \mathrm{g} \mathrm{H}$	12–16 %	5	5
	2-jet $\mathrm{VBF}$	75–83 % $\mathrm{VBF}$	13–16 %	2	4
$\mathrm{\tau }_{\mathrm{h}} \mathrm{\tau }_{\mathrm{h}}$	1-jet	67–70 % $\mathrm{g} \mathrm{g} \mathrm{H}$	10–12 %	–	2
	2-jet $\mathrm{VBF}$	80 % $\mathrm{VBF}$	11 %	–	1
$\mathrm{e}\mathrm{\mu }$	0-jet	${\approx }$98 % $\mathrm{g} \mathrm{g} \mathrm{H}$, 23–30 % $\mathrm{W}\mathrm{W}$	16–20 %	2	2
	1-jet	75–80 % $\mathrm{g} \mathrm{g} \mathrm{H}$, 31–38 % $\mathrm{W}\mathrm{W}$	18–19 %	2	2
	2-jet $\mathrm{VBF}$	79–94 % $\mathrm{VBF}$, 37–45 % $\mathrm{W}\mathrm{W}$	14–19 %	1	2
$\mathrm{e}\mathrm{e}$, $\mathrm{\mu }\mathrm{\mu }$	0-jet	88–98 % $\mathrm{g} \mathrm{g} \mathrm{H}$		4	4
	1-jet	74–78 % $\mathrm{g} \mathrm{g} \mathrm{H}$, ${\approx }17\,\%$ $\mathrm{W}\mathrm{W}$ $^{\star }$		4	4
	2-jet CJV	${\approx }50\,\%$ $\mathrm{VBF}$, ${\approx }45\,\%$ $\mathrm{g} \mathrm{g} \mathrm{H}$, 17–24 % $\mathrm{W}\mathrm{W}$ $^{\star }$		2	2
$\ell \ell +LL^{\prime }$ ($\mathrm{Z}\mathrm{H}$)	$LL^{\prime }=\mathrm{\tau }_{\mathrm{h}} \mathrm{\tau }_{\mathrm{h}} ,\ell \mathrm{\tau }_{\mathrm{h}} ,\mathrm{e}\mathrm{\mu }$	${\approx }15\,\%$ (70 %) $\mathrm{W}\mathrm{W}$ for $LL^{\prime }=\ell \mathrm{\tau }_{\mathrm{h}}$ $(\mathrm{e}\mathrm{\mu })$		8	8
$\ell +\mathrm{\tau }_{\mathrm{h}} \mathrm{\tau }_{\mathrm{h}}$ ($\mathrm{W}\mathrm{H}$)		${\approx }96\,\%$ $\mathrm{V}\mathrm{H}$, $\mathrm{Z}\mathrm{H} /\mathrm{W}\mathrm{H} \approx 0.1$		2	2
$\ell +\ell ^{\prime }\mathrm{\tau }_{\mathrm{h}}$ ($\mathrm{W}\mathrm{H}$)		$\mathrm{Z}\mathrm{H} /\mathrm{W}\mathrm{H} \approx 5\,\%$, 9–11 % $\mathrm{W}\mathrm{W}$		2	4
$\mathrm{V}\mathrm{H}$ production with $\mathrm{H} \rightarrow \mathrm{b} \mathrm{b}$ [21], Sect. 2.5				*5.1*	*18.9*
$\mathrm{W}(\ell \nu )\mathrm{H} (\mathrm{b} \mathrm{b} )$	$p_{\mathrm{T}} ({\mathrm{V}})$ bins	${\approx }$100 % $\mathrm{V}\mathrm{H}$, 96–98 % $\mathrm{W}\mathrm{H}$		4	6
$\mathrm{W}(\mathrm{\tau }_{\mathrm{h}} \nu )\mathrm{H} (\mathrm{b} \mathrm{b} )$	–	93 % $\mathrm{W}\mathrm{H}$		–	1
$\mathrm{Z}(\ell \ell )\mathrm{H} (\mathrm{b} \mathrm{b} )$	$p_{\mathrm{T}} ({\mathrm{V}})$ bins	${\approx }$100 % $\mathrm{Z}\mathrm{H}$		4	4
$\mathrm{Z}(\nu \nu )\mathrm{H} (\mathrm{b} \mathrm{b} )$	$p_{\mathrm{T}} ({\mathrm{V}})$ bins	${\approx }$100 % $\mathrm{V}\mathrm{H}$, 62–76 % $\mathrm{Z}\mathrm{H}$	${\approx }10\,\%$	2	3
$\mathrm{t}\mathrm{t}\mathrm{H}$ production with $\mathrm{H} \rightarrow \text {hadrons}$ or $\mathrm{H} \rightarrow \text {leptons}$ [29], Sect. 2.6				*5.0*	$\le$ *19.6*
	$\mathrm{t}\overline{\mathrm{t}}$ lepton+jets	${\approx }90\,\%$ $\mathrm{b} \mathrm{b}$ but ${\approx }24\,\%$ $\mathrm{W}\mathrm{W}$ in ${\ge }6{\mathrm{j}}+2{\mathrm{b}}$		7	7
$\mathrm{H} \rightarrow \mathrm{b} \mathrm{b}$	$\mathrm{t}\overline{\mathrm{t}}$ dilepton	45–85 % $\mathrm{b} \mathrm{b}$, 8–35 % $\mathrm{W}\mathrm{W}$, 4–14 % $\tau \tau$		2	3
$\mathrm{H} \rightarrow \mathrm{\tau }_{\mathrm{h}} \mathrm{\tau }_{\mathrm{h}}$	$\mathrm{t}\overline{\mathrm{t}}$ lepton+jets	68–80 % $\tau \tau$, 13–22 % $\mathrm{W}\mathrm{W}$, 5–13 % $\mathrm{b} \mathrm{b}$		–	6
$2\ell$ SS		$\mathrm{W}\mathrm{W}/\tau \tau \approx 3$		–	6
$3\ell$		$\mathrm{W}\mathrm{W}/\tau \tau \approx 3$		–	2
$4\ell$	$\ge 2$ jets, $\ge 1$ b jet	$\mathrm{W}\mathrm{W}:\tau \tau :\mathrm{Z}\mathrm{Z} \approx 3:2:1$		–	1
$\mathrm{H} \rightarrow {\mathrm{invisible}}$ [28], Sect. 2.7				*4.9*	$\le$ *19.7*
$\mathrm{H} ({\mathrm{inv}})$	2-jet VBF	${\approx }94\,\%$ $\mathrm{VBF}$, ${\approx }6\,\%$ $\mathrm{g} \mathrm{g} \mathrm{H}$		–	1
	0-jet			2	2
$\mathrm{Z}\mathrm{H} \rightarrow \mathrm{Z}(\mathrm{e}\mathrm{e},\mu \mu )\mathrm{H} ({\mathrm{inv}})$	1-jet	${\approx }100\,\%$ $\mathrm{Z}\mathrm{H}$		2	2
$\mathrm{H} \rightarrow \mu \mu$ [30], Sect. 2.8				*5.0*	*19.7*
	Untagged	88–99 % $\mathrm{g} \mathrm{g} \mathrm{H}$	1.3–2.4 %	12	12
	2-jet $\mathrm{VBF}$	${\approx }80\,\%$ $\mathrm{VBF}$	1.9 %	1	1
	2-jet boosted	${\approx }50\,\%$ $\mathrm{g} \mathrm{g} \mathrm{H}$, ${\approx }50\,\%$ $\mathrm{VBF}$	1.8 %	1	1
$\mu \mu$	2-jet other	${\approx }68\,\%$ $\mathrm{g} \mathrm{g} \mathrm{H}$, ${\approx }17\,\%$ $\mathrm{V}\mathrm{H}$, ${\approx }15\,\%$ $\mathrm{VBF}$	1.9 %	1	1

$^{\dagger }$ Events fulfilling the requirements of either selection are combined into one category

$^{\ddagger }$ Values for analyses dedicated to the measurement of the mass that do not use the same categories and/or observables

$^{\star }$ Composition in the regions for which the ratio of signal and background $s/(s+b)>0.05$

### $\mathrm{H} \rightarrow \gamma \gamma$

The $\mathrm{H} \rightarrow \gamma \gamma$ analysis [18, 174] measures a narrow signal mass peak situated on a smoothly falling background due to events originating from prompt nonresonant diphoton production or due to events with at least one jet misidentified as an isolated photon.

The sample of selected events containing a photon pair is split into mutually exclusive event categories targeting the different Higgs boson production processes, as listed in Table 1. Requiring the presence of two jets with a large rapidity gap favours events produced by the $\mathrm{VBF}$ mechanism, while event categories designed to preferentially select $\mathrm{V}\mathrm{H}$ or $\mathrm{t}\mathrm{t}\mathrm{H}$ production require the presence of muons, electrons, $E_{\mathrm{T}}^{\text {miss}}$, a pair of jets compatible with the decay of a vector boson, or jets arising from the hadronization of bottom quarks. For 7$\,\text {TeV}$ data, only one $\mathrm{t}\mathrm{t}\mathrm{H}$-tagged event category is used, combining the events selected by the leptonic $\mathrm{t}\mathrm{t}\mathrm{H}$ and multijet $\mathrm{t}\mathrm{t}\mathrm{H}$ selections. The 2-jet $\mathrm{VBF}$-tagged categories are further split according to a multivariate (MVA) classifier that is trained to discriminate $\mathrm{VBF}$ events from both background and $\mathrm{g} \mathrm{g} \mathrm{H}$ events.

Fewer than 1 % of the selected events are tagged according to production mode. The remaining “untagged” events are subdivided into different categories based on the output of an MVA classifier that assigns a high score to signal-like events and to events with a good mass resolution, based on a combination of (i) an event-by-event estimate of the diphoton mass resolution, (ii) a photon identification score for each photon, and (iii) kinematic information about the photons and the diphoton system. The photon identification score is obtained from a separate MVA classifier that uses shower shape information and variables characterizing how isolated the photon candidate is to discriminate prompt photons from those arising in jets.

The same event categories and observables are used for the mass measurement and to search for deviations in the magnitudes of the scalar couplings of the Higgs boson.

In each event category, the background in the signal region is estimated from a fit to the observed diphoton mass distribution in data. The uncertainty due to the choice of function used to describe the background is incorporated into the statistical procedure: the likelihood maximization is also performed for a discrete variable that selects which of the functional forms is evaluated. This procedure is found to have correct coverage probability and negligible bias in extensive tests using pseudo-data extracted from fits of multiple families of functional forms to the data. By construction, this “discrete profiling” of the background functional form leads to confidence intervals for any estimated parameter that are at least as large as those obtained when considering any single functional form. Uncertainty in the parameters of the background functional forms contributes to the statistical uncertainty of the measurements.

### $\mathrm{H} \rightarrow \mathrm{Z}\mathrm{Z}$

In the $\mathrm{H} \rightarrow \mathrm{Z}\mathrm{Z} \rightarrow 4\ell$ analysis [16, 175], we measure a four-lepton mass peak over a small continuum background. To further separate signal and background, we build a discriminant, $\mathcal {D}^{\text {kin}}_\text {bkg}$, using the leading-order matrix elements for signal and background. The value of $\mathcal {D}^{\text {kin}}_\text {bkg}$ is calculated from the observed kinematic variables, namely the masses of the two dilepton pairs and five angles, which uniquely define a four-lepton configuration in its centre-of-mass frame.

Given the different mass resolutions and different background rates arising from jets misidentified as leptons, the $4\mathrm{\mu }$, $2\mathrm{e}2\mathrm{\mu }/2\mathrm{\mu }2\mathrm{e}$, and $4\mathrm{e}$ event categories are analysed separately. A stricter dilepton mass selection is performed for the lepton pair with invariant mass closest to the nominal $\mathrm{Z}$ boson mass.

The dominant irreducible background in this channel is due to nonresonant $\mathrm{Z}\mathrm{Z}$ production with both $\mathrm{Z}$ bosons decaying to a pair of charged leptons and is estimated from simulation. The smaller reducible backgrounds with misidentified leptons, mainly from the production of $\mathrm{Z}+\text {jets}$, top quark pairs, and $\mathrm{W}\mathrm{Z}+\text {jets}$, are estimated from data.

For the mass measurement an event-by-event estimator of the mass resolution is built from the single-lepton momentum resolutions evaluated from the study of a large number of $\mathrm{J}/\psi \rightarrow \mu \mu$ and $\mathrm{Z}\rightarrow \ell \ell$ data events. The relative mass resolution, $\sigma _{m_{4\ell }}/m_{4\ell }$, is then used together with $m_{4\ell }$ and $\mathcal {D}^{\text {kin}}_\text {bkg}$ to measure the mass of the boson.

To increase the sensitivity to the different production mechanisms, the event sample is split into two categories based on jet multiplicity: (i) events with fewer than two jets and (ii) events with at least two jets. In the first category, the four-lepton transverse momentum is used to discriminate $\mathrm{VBF}$ and $\mathrm{V}\mathrm{H}$ production from $\mathrm{g} \mathrm{g} \mathrm{H}$ production. In the second category, a linear discriminant, built from the values of the invariant mass of the two leading jets and their pseudorapidity difference, is used to separate the $\mathrm{VBF}$ and $\mathrm{g} \mathrm{g} \mathrm{H}$ processes.

### $\mathrm{H} \rightarrow \mathrm{W}\mathrm{W}$

In the $\mathrm{H} \rightarrow \mathrm{W}\mathrm{W}$ analysis [22], we measure an excess of events with two OS leptons or three charged leptons with a total charge of $\pm 1$, moderate $E_{\mathrm{T}}^{\text {miss}}$, and up to two jets.

The two-lepton events are divided into eight categories, with different background compositions and signal-to-background ratios. The events are split into SF and DF dilepton event categories, since the background from Drell–Yan production ($\mathrm{q} \mathrm{q} \rightarrow \gamma ^{*}/\mathrm{Z}^{(*)} \rightarrow \ell \ell$) is much larger for SF dilepton events. For events with no jets, the main background is due to nonresonant $\mathrm{W}\mathrm{W}$ production. For events with one jet, the dominant backgrounds are nonresonant $\mathrm{W}\mathrm{W}$ production and top quark production. The 2-jet $\mathrm{VBF}$ tag is optimized to take advantage of the $\mathrm{VBF}$ production signature and the main background is due to top quark production. The 2-jet $\mathrm{V}\mathrm{H}$ tag targets the decay of the vector boson into two jets, ${\mathrm{V}}\rightarrow \text {jj}$. The selection requires two centrally-produced jets with invariant mass in the range $65<m_{\text {jj}}<105\,\text {GeV}$. To reduce the top quark, Drell–Yan, and $\mathrm{W}\mathrm{W}$ backgrounds in all previous categories, a selection is performed on the dilepton mass and on the angular separation between the leptons. All background rates, except for very small contributions from $\mathrm{W}\mathrm{Z}$, $\mathrm{Z}\mathrm{Z}$, and $\mathrm{W}\mathrm{\gamma }$ production, are evaluated from data. The two-dimensional distribution of events in the $(m_{\ell \ell },m_{\text {T}})$ plane is used for the measurements in the DF dilepton categories with zero and one jets; $m_{\ell \ell }$ is the invariant mass of the dilepton and $m_{\text {T}}$ is the transverse mass reconstructed from the dilepton transverse momentum and the $E_{\mathrm{T}}^{\text {miss}}$ vector. For the DF 2-jet $\mathrm{VBF}$ tag the binned distribution of $m_{\ell \ell }$ is used. For the SF dilepton categories and for the 2-jet $\mathrm{V}\mathrm{H}$ tag channel, only the total event counts are used.

In the $3\ell 3\nu$ channel targeting the $\mathrm{W}\mathrm{H} \rightarrow \mathrm{W}\mathrm{W}\mathrm{W}$ process, we search for an excess of events with three leptons, electrons or muons, large $E_{\mathrm{T}}^{\text {miss}}$, and low hadronic activity. The dominant background is due to $\mathrm{W}\mathrm{Z} \rightarrow 3\ell \nu$ production, which is largely reduced by requiring that all SF and OS lepton pairs have invariant masses away from the $\mathrm{Z}$ boson mass. The smallest angular distance between OS reconstructed lepton tracks is the observable chosen to perform the measurement. The background processes with jets misidentified as leptons, e.g. $\mathrm{Z}+\text {jets}$ and top quark production, as well as the $\mathrm{W}\mathrm{Z} \rightarrow 3\ell \nu$ background, are estimated from data. The small contribution from the $\mathrm{Z}\mathrm{Z} \rightarrow 4\ell$ process with one of the leptons escaping detection is estimated using simulated samples. In the $3\ell 3\nu$ channel, up to 20 % of the signal events are expected to be due to $\mathrm{H} \rightarrow \tau \tau$ decays.

In the $3\ell \nu \text {jj}$ channel, targeting the $\mathrm{Z}\mathrm{H} \rightarrow \mathrm{Z}+\mathrm{W}\mathrm{W}\rightarrow \ell \ell +\ell ^{\prime }\nu \text {jj}$ process, we first identify the leptonic decay of the $\mathrm{Z}$ boson and then require the dijet system to satisfy $|m_{\text {jj}}-m_{\mathrm{W}}|\le 60\,\text {GeV}$. The transverse mass of the $\ell \nu \text {jj}$ system is the observable chosen to perform the measurement. The main backgrounds are due to the production of $\mathrm{W}\mathrm{Z}$, $\mathrm{Z}\mathrm{Z}$, and tribosons, as well as processes involving nonprompt leptons. The first three are estimated from simulated samples, while the last one is evaluated from data.

Finally, a dedicated analysis for the measurement of the boson mass is performed in the 0-jet and 1-jet categories in the $\mathrm{e}\mu$ channel, employing observables that are extensively used in searches for supersymmetric particles. A resolution of 16–17 % for $m_\mathrm{{H}} =125\,\text {GeV}$ has been achieved.

### $\mathrm{H} \rightarrow \tau \tau$

The $\mathrm{H} \rightarrow \tau \tau$ analysis [23] measures an excess of events over the SM background expectation using multiple final-state signatures. For the $\mathrm{e}\mathrm{\mu }$, $\mathrm{e}\mathrm{\tau }_{\mathrm{h}}$, $\mathrm{\mu }\mathrm{\tau }_{\mathrm{h}}$, and $\mathrm{\tau }_{\mathrm{h}} \mathrm{\tau }_{\mathrm{h}}$ final states, where electrons and muons arise from leptonic $\mathrm{\tau }$ decays, the event samples are further divided into categories based on the number of reconstructed jets in the event: 0 jets, 1 jet, or 2 jets. The 0-jet and 1-jet categories are further subdivided according to the reconstructed $p_{\mathrm{T}}$ of the leptons. The 2-jet categories require a $\mathrm{VBF}$-like topology and are subdivided according to selection criteria applied to the dijet kinematic properties. In each of these categories, we search for a broad excess in the reconstructed $\tau \tau$ mass distribution. The 0-jet category is used to constrain background normalizations, identification efficiencies, and energy scales. Various control samples in data are used to evaluate the main irreducible background from $\mathrm{Z}\rightarrow \tau \tau$ production and the largest reducible backgrounds from $\mathrm{W}+\text {jets}$ and multijet production. The $\mathrm{e}\mathrm{e}$ and $\mathrm{\mu }\mathrm{\mu }$ final states are similarly subdivided into jet categories as above, but the search is performed on the combination of two MVA discriminants. The first is trained to distinguish $\mathrm{Z}\rightarrow \ell \ell$ events from $\mathrm{Z}\rightarrow \tau \tau$ events while the second is trained to separate $\mathrm{Z}\rightarrow \tau \tau$ events from $\mathrm{H} \rightarrow \tau \tau$ events. The expected SM Higgs boson signal in the $\mathrm{e}\mathrm{\mu }$, $\mathrm{e}\mathrm{e}$, and $\mathrm{\mu }\mathrm{\mu }$ categories has a sizeable contribution from $\mathrm{H} \rightarrow \mathrm{W}\mathrm{W}$ decays: 17–24 % in the $\mathrm{e}\mathrm{e}$ and $\mathrm{\mu }\mathrm{\mu }$ event categories, and 23–45 % in the $\mathrm{e}\mathrm{\mu }$ categories, as shown in Table 1.

The search for $\tau \tau$ decays of Higgs bosons produced in association with a $\mathrm{W}$ or $\mathrm{Z}$ boson is conducted in events where the vector bosons are identified through the $\mathrm{W}\rightarrow \ell \nu$ or $\mathrm{Z}\rightarrow \ell \ell$ decay modes. The analysis targeting $\mathrm{W}\mathrm{H}$ production selects events that have electrons or muons and one or two hadronically decaying tau leptons: $\mathrm{\mu }+\mathrm{\mu }\mathrm{\tau }_{\mathrm{h}}$, $\mathrm{e}+\mathrm{\mu }\mathrm{\tau }_{\mathrm{h}}$ or $\mathrm{\mu }+\mathrm{e}\mathrm{\tau }_{\mathrm{h}}$, $\mathrm{\mu }+\mathrm{\tau }_{\mathrm{h}} \mathrm{\tau }_{\mathrm{h}}$, and $\mathrm{e}+\mathrm{\tau }_{\mathrm{h}} \mathrm{\tau }_{\mathrm{h}}$. The analysis targeting $\mathrm{Z}\mathrm{H}$ production selects events with an identified $\mathrm{Z}\rightarrow \ell \ell$ decay and a Higgs boson candidate decaying to $\mathrm{e}\mathrm{\mu }$, $\mathrm{e}\mathrm{\tau }_{\mathrm{h}}$, $\mathrm{\mu }\mathrm{\tau }_{\mathrm{h}}$, or $\mathrm{\tau }_{\mathrm{h}} \mathrm{\tau }_{\mathrm{h}}$. The main irreducible backgrounds to the $\mathrm{W}\mathrm{H}$ and $\mathrm{Z}\mathrm{H}$ searches are $\mathrm{W}\mathrm{Z}$ and $\mathrm{Z}\mathrm{Z}$ diboson events, respectively. The irreducible backgrounds are estimated using simulated event samples corrected by measurements from control samples in data. The reducible backgrounds in both analyses are due to the production of $\mathrm{W}$ bosons, $\mathrm{Z}$ bosons, or top quark pairs with at least one jet misidentified as an isolated $\mathrm{e}$, $\mathrm{\mu }$, or $\mathrm{\tau }_{\mathrm{h}}$. These backgrounds are estimated exclusively from data by measuring the probability for jets to be misidentified as isolated leptons in background-enriched control regions, and weighting the selected events that fail the lepton requirements with the misidentification probability. For the SM Higgs boson, the expected fraction of $\mathrm{H} \rightarrow \mathrm{W}\mathrm{W}$ events in the $\mathrm{Z}\mathrm{H}$ analysis is 10–15 % for the $\mathrm{Z}\mathrm{H} \rightarrow \mathrm{Z}+\ell \mathrm{\tau }_{\mathrm{h}}$ channel and 70 % for the $\mathrm{Z}\mathrm{H} \rightarrow \mathrm{Z}+\mathrm{e}\mathrm{\mu }$ channel, as shown in Table 1.

### $\mathrm{V}\mathrm{H}$ with $\mathrm{H} \rightarrow \mathrm{b} \mathrm{b}$

Exploiting the large expected $\mathrm{H} \rightarrow \mathrm{b} \mathrm{b}$ branching fraction, the analysis of $\mathrm{V}\mathrm{H}$ production and $\mathrm{H} \rightarrow \mathrm{b} \mathrm{b}$ decay examines the $\mathrm{W}(\ell \nu )\mathrm{H} (\mathrm{b} \mathrm{b} )$, $\mathrm{W}(\mathrm{\tau }_{\mathrm{h}} \nu )\mathrm{H} (\mathrm{b} \mathrm{b} )$, $\mathrm{Z}(\ell \ell )\mathrm{H} (\mathrm{b} \mathrm{b} )$, and $\mathrm{Z}(\nu \nu )\mathrm{H} (\mathrm{b} \mathrm{b} )$ topologies [21].

The Higgs boson candidate is reconstructed by requiring two b-tagged jets. The event sample is divided into categories defined by the transverse momentum of the vector boson, $p_{\mathrm{T}} ({\mathrm{V}})$. An MVA regression is used to estimate the true energy of the bottom quark after being trained on reconstructed b jets in simulated $\mathrm{H} \rightarrow \mathrm{b} \mathrm{b}$ events. This regression algorithm achieves a dijet mass resolution of about 10 % for $m_\mathrm{{H}} = 125\,\text {GeV}$. The performance of the regression algorithm is checked with data, where it is observed to improve the top quark mass scale and resolution in top quark pair events and to improve the $p_{\mathrm{T}}$ balance between a $\mathrm{Z}$ boson and b jets in $\mathrm{Z}(\rightarrow \ell \ell )+\mathrm{b} \mathrm{b}$ events. Events with higher $p_{\mathrm{T}} ({\mathrm{V}})$ have smaller backgrounds and better dijet mass resolution. A cascade of MVA classifiers, trained to distinguish the signal from top quark pairs, $\text {V}+\text {jets}$, and diboson events, is used to improve the sensitivity in the $\mathrm{W}(\ell \nu )\mathrm{H} (\mathrm{b} \mathrm{b} )$, $\mathrm{W}(\mathrm{\tau }_{\mathrm{h}} \nu )\mathrm{H} (\mathrm{b} \mathrm{b} )$, and $\mathrm{Z}(\nu \nu )\mathrm{H} (\mathrm{b} \mathrm{b} )$ channels. The rates of the main backgrounds, consisting of $\text {V}+\text {jets}$ and top quark pair events, are derived from signal-depleted data control samples. The $\mathrm{W}\mathrm{Z}$ and $\mathrm{Z}\mathrm{Z}$ backgrounds where $\mathrm{Z}\rightarrow \mathrm{b} \mathrm{b}$, as well as the single top quark background, are estimated from simulated samples. The MVA classifier output distribution is used as the final discriminant in performing measurements.

At the time of publication of Ref. [21], the simulation of the $\mathrm{Z}\mathrm{H}$ signal process included only $\mathrm{q}\mathrm{\overline{q}}$-initiated diagrams. Since then, a more accurate prediction of the $p_{\mathrm{T}} (\mathrm{Z})$ distribution has become available, taking into account the contribution of the gluon-gluon initiated associated production process $\mathrm{g} \mathrm{g} \rightarrow \mathrm{Z}\mathrm{H}$, which is included in the results presented in this paper. The calculation of the $\mathrm{g} \mathrm{g} \rightarrow \mathrm{Z}\mathrm{H}$ contribution includes next-to-leading order (NLO) effects [176–179] and is particularly important given that the $\mathrm{g} \mathrm{g} \rightarrow \mathrm{Z}\mathrm{H}$ process contributes to the most sensitive categories of the analysis. This treatment represents a significant improvement with respect to Ref. [21], as discussed in Sect. 3.4.

### $\mathrm{t}\mathrm{t}\mathrm{H}$ production

Given its distinctive signature, the $\mathrm{t}\mathrm{t}\mathrm{H}$ production process can be tagged using the decay products of the top quark pair. The search for $\mathrm{t}\mathrm{t}\mathrm{H}$ production is performed in four main channels: $\mathrm{H} \rightarrow \gamma \gamma$, $\mathrm{H} \rightarrow \mathrm{b} \mathrm{b}$, $\mathrm{H} \rightarrow \mathrm{\tau }_{\mathrm{h}} \mathrm{\tau }_{\mathrm{h}}$, and $\mathrm{H} \rightarrow \text {leptons}$  [19, 29]. The $\mathrm{t}\mathrm{t}\mathrm{H}$ search in $\mathrm{H} \rightarrow \gamma \gamma$ events is described in Sect. 2.1; the following focuses on the other three topologies.

In the analysis of $\mathrm{t}\mathrm{t}\mathrm{H}$ production with $\mathrm{H} \rightarrow \mathrm{b} \mathrm{b}$, two signatures for the top quark pair decay are considered: lepton+jets ($\mathrm{t}\overline{\mathrm{t}} \rightarrow \ell \nu \text {jj}\text {bb}$) and dilepton ($\mathrm{t}\overline{\mathrm{t}} \rightarrow \ell \nu \ell \nu \text {bb}$). In the analysis of $\mathrm{t}\mathrm{t}\mathrm{H}$ production with $\mathrm{H} \rightarrow \mathrm{\tau }_{\mathrm{h}} \mathrm{\tau }_{\mathrm{h}}$, the $\mathrm{t}\overline{\mathrm{t}}$ lepton+jets decay signature is required. In both channels, the events are further classified according to the numbers of identified jets and b-tagged jets. The major background is from top-quark pair production accompanied by extra jets. An MVA is trained to discriminate between background and signal events using information related to reconstructed object kinematic properties, event shape, and the discriminant output from the b-tagging algorithm. The rates of background processes are estimated from simulated samples and are constrained through a simultaneous fit to background-enriched control samples.

The analysis of $\mathrm{t}\mathrm{t}\mathrm{H}$ production with $\mathrm{H} \rightarrow \text {leptons}$ is mainly sensitive to Higgs boson decays to $\mathrm{W}\mathrm{W}$, $\tau \tau$, and $\mathrm{Z}\mathrm{Z}$, with subsequent decay to electrons and/or muons. The selection starts by requiring the presence of at least two central jets and at least one b jet. It then proceeds to categorize the events according to the number, charge, and flavour of the reconstructed leptons: $2\ell$ SS, $3\ell$ with a total charge of $\pm 1$, and $4\ell$. A dedicated MVA lepton selection is used to suppress the reducible background from nonprompt leptons, usually from the decay of b hadrons. After the final selection, the two main sources of background are nonprompt leptons, which is evaluated from data, and associated production of top quark pairs and vector bosons, which is estimated from simulated samples. Measurements in the $4\ell$ event category are performed using the number of reconstructed jets, $N_\text {j}$. In the $2\ell$ SS and $3\ell$ categories, an MVA classifier is employed, which makes use of $N_\text {j}$ as well as other kinematic and event shape variables to discriminate between signal and background.

### Searches for Higgs boson decays into invisible particles

The search for a Higgs boson decaying into particles that escape direct detection, denoted as $\mathrm{H} ({\mathrm{inv}})$ in what follows, is performed using $\mathrm{VBF}$-tagged events and $\mathrm{Z}\mathrm{H}$-tagged events [28]. The $\mathrm{Z}\mathrm{H}$ production mode is tagged via the $\mathrm{Z}\rightarrow \ell \ell$ or $\mathrm{Z}\rightarrow \mathrm{b} \mathrm{b}$ decays. For this combined analysis, only the $\mathrm{VBF}$-tagged and $\mathrm{Z}\rightarrow \ell \ell$ channels are used; the event sample of the less sensitive $\mathrm{Z}\rightarrow \mathrm{b} \mathrm{b}$ analysis overlaps with that used in the analysis of $\mathrm{V}\mathrm{H}$ with $\mathrm{H} \rightarrow \mathrm{b} \mathrm{b}$ decay described in Sect. 2.5 and is not used in this combined analysis.

The $\mathrm{VBF}$-tagged event selection is performed only on the 8$\,\text {TeV}$ data and requires a dijet mass above 1100$\,\text {GeV}$ as well as a large separation of the jets in pseudorapidity, $\eta$. The $E_{\mathrm{T}}^{\text {miss}}$ is required to be above 130$\,\text {GeV}$ and events with additional jets with $p_{\mathrm{T}} >30\,\text {GeV}$ and a value of $\eta$ between those of the tagging jets are rejected. The single largest background is due to the production of $\mathrm{Z}(\nu \nu )+\text {jets}$ and is estimated from data using a sample of events with visible $\mathrm{Z}\rightarrow \mathrm{\mu }\mathrm{\mu }$ decays that also satisfy the dijet selection requirements above. To extract the results, a one bin counting experiment is performed in a region where the expected signal-to-background ratio is 0.7, calculated assuming the Higgs boson is produced with the SM cross section but decays only into invisible particles.

The event selection for $\mathrm{Z}\mathrm{H}$ with $\mathrm{Z}\rightarrow \ell \ell$ rejects events with two or more jets with $p_{\mathrm{T}} >30\,\text {GeV}$. The remaining events are categorized according to the $\mathrm{Z}$ boson decay into $\mathrm{e}\mathrm{e}$ or $\mu \mu$ and the number of identified jets, zero or one. For the 8$\,\text {TeV}$ data, the results are extracted from a two-dimensional fit to the azimuthal angular difference between the leptons and the transverse mass of the system composed of the dilepton and the missing transverse energy in the event. Because of the smaller amount of data in the control samples used for modelling the backgrounds in the signal region, the results for the 7$\,\text {TeV}$ data set are based on a fit to the aforementioned transverse mass variable only. For the 0-jet categories the signal-to-background ratio varies between 0.24 and 0.28, while for the 1-jet categories it varies between 0.15 and 0.18, depending on the $\mathrm{Z}$ boson decay channel and the data set (7 or 8$\,\text {TeV}$). The signal-to-background ratio increases as a function of the transverse mass variable.

The data from these searches are used for results in Sects. 7.5 and 7.8, where the partial widths for invisible and/or undetected decays of the Higgs boson are probed.

### $\mathrm{H} \rightarrow \mu \mu$

The $\mathrm{H} \rightarrow \mu \mu$ analysis [30] is a search in the distribution of the dimuon invariant mass, $m_{\mu \mu }$, for a narrow signal peak over a smoothly falling background dominated by Drell–Yan and top quark pair production. A sample of events with a pair of OS muons is split into mutually exclusive categories of differing expected signal-to-background ratios, based on the event topology and kinematic properties. Events with two or more jets are assigned to 2-jet categories, while the remaining events are assigned to untagged categories. The 2-jet events are divided into three categories using selection criteria based on the properties of the dimuon and the dijet systems: a VBF-tagged category, a boosted dimuon category, and a category with the remaining 2-jet events. The untagged events are distributed among twelve categories based on the dimuon $p_{\mathrm{T}}$ and the pseudorapidity of the two muons, which are directly related to the $m_{\mu \mu }$ experimental resolution.

The $m_{\mu \mu }$ spectrum in each event category is fitted with parameterized signal and background shapes to estimate the number of signal events, in a procedure similar to that of the $\mathrm{H} \rightarrow \gamma \gamma$ analysis, described in Sect. 2.1. The uncertainty due to the choice of the functional form used to model the background is incorporated in a different manner than in the $\mathrm{H} \rightarrow \gamma \gamma$ analysis, namely by introducing an additive systematic uncertainty in the number of expected signal events. This uncertainty is estimated by evaluating the bias of the signal function plus nominal background function when fitted to pseudo-data generated from alternative background functions. The largest absolute value of this difference for all the alternative background functions considered and Higgs boson mass hypotheses between 120 and 150$\,\text {GeV}$ is taken as the systematic uncertainty and applied uniformly for all Higgs boson mass hypotheses. The effect of these systematic uncertainties on the final result is sizeable, about 75 % of the overall statistical uncertainty.

The data from this analysis are used for the results in Sect. 7.4, where the scaling of the couplings with the mass of the involved particles is explored.

## Combination methodology

The combination of Higgs boson measurements requires the simultaneous analysis of the data selected by all individual analyses, accounting for all statistical uncertainties, systematic uncertainties, and their correlations.

The overall statistical methodology used in this combination was developed by the ATLAS and CMS Collaborations in the context of the LHC Higgs Combination Group and is described in Refs. [15, 180, 181]. The chosen test statistic, $q$, is based on the profile likelihood ratio and is used to determine how signal-like or background-like the data are. Systematic uncertainties are incorporated in the analysis via nuisance parameters that are treated according to the frequentist paradigm. Below we give concise definitions of statistical quantities that we use for characterizing the outcome of the measurements. Results presented herein are obtained using asymptotic formulae [182], including routines available in the RooStats package [183].

### Characterizing an excess of events: $p\text {-value}$ and significance

To quantify the presence of an excess of events over the expected background we use the test statistic where the likelihood appearing in the numerator corresponds to the background-only hypothesis:

1 $$\begin{aligned} q_{0} = - 2 \ln \frac{\mathcal {L}(\text {data} \, | \, b, \, \hat{\theta }_{0} ) }{\mathcal {L}(\text {data} \, | \, \hat{\mu }\, s + b, \, \hat{\theta }) },\quad \text {with }\hat{\mu }>0, \end{aligned}$$

where $s$ stands for the signal expected for the SM Higgs boson, $\mu$ is a signal strength modifier introduced to accommodate deviations from the SM Higgs boson predictions, $b$ stands for backgrounds, and $\theta$ represents nuisance parameters describing systematic uncertainties. The value $\hat{\theta }_{0}$ maximizes the likelihood in the numerator under the background-only hypothesis, $\mu =0$, while $\hat{\mu }$ and $\hat{\theta }$ define the point at which the likelihood reaches its global maximum.

The quantity $p_0$, henceforth referred to as the local $p\text {-value}$, is defined as the probability, under the background-only hypothesis, to obtain a value of $q_0$ at least as large as that observed in data, $q_0^\text {data}$:

2 $$\begin{aligned} p_0 = {\mathrm{P}}\left( q_0 \ge q_0^\text {data} \, \Big | \, b\right) . \end{aligned}$$

The local significance $z$ of a signal-like excess is then computed according to the one-sided Gaussian tail convention:

3 $$\begin{aligned} p_0 = \int _{z}^{+\infty } \frac{1}{\sqrt{2\pi }} \exp (-x^2/2) \, \mathrm{d}x. \end{aligned}$$

It is important to note that very small $p\text {-values}$ should be interpreted with caution, since systematic biases and uncertainties in the underlying model are only known to a given precision.

### Extracting signal model parameters

Signal model parameters $a$, such as the signal strength modifier $\mu$, are evaluated from scans of the profile likelihood ratio $q(a)$:

4 $$\begin{aligned} q(a) = -2 \Delta \ln \mathcal {L} = - 2 \ln \frac{\mathcal {L}(\text {data} \, | \, s(a) + b, \, \hat{\theta }_{a} ) }{\mathcal {L}(\text {data} \, | \, s(\hat{a}) + b, \, \hat{\theta }) } . \end{aligned}$$

The parameter values $\hat{a}$ and $\hat{\theta }$ correspond to the global maximum likelihood and are called the best-fit set. The post-fit model, obtained using the best-fit set, is used when deriving expected quantities. The post-fit model corresponds to the parametric bootstrap described in the statistics literature and includes information gained in the fit regarding the values of all parameters [184, 185].

The 68 and 95 % confidence level (CL) confidence intervals for a given parameter of interest, $a_i$, are evaluated from $q(a_i)=1.00$ and $q(a_i)=3.84$, respectively, with all other unconstrained model parameters treated in the same way as the nuisance parameters. The two-dimensional (2D) 68 and 95 % CL confidence regions for pairs of parameters are derived from $q(a_i, a_j) = 2.30$ and $q(a_i, a_j) = 5.99$, respectively. This implies that boundaries of 2D confidence regions projected on either parameter axis are not identical to the one-dimensional (1D) confidence interval for that parameter. All results are given using the chosen test statistic, leading to approximate CL confidence intervals when there are no large non-Gaussian uncertainties [186–188], as is the case here. If the best-fit value is on a physical boundary, the theoretical basis for computing intervals in this manner is lacking. However, we have found that for the results in this paper, the intervals in those conditions are numerically similar to those obtained by the method of Ref. [189].

### Grouping of channels by decay and production tags

The event samples selected by each of the different analyses are mutually exclusive. The selection criteria can, in many cases, define high-purity selections of the targeted decay or production modes, as shown in Table 1. For example, the $\mathrm{t}\mathrm{t}\mathrm{H}$-tagged event categories of the $\mathrm{H} \rightarrow \gamma \gamma$ analysis are pure in terms of $\gamma \gamma$ decays and are expected to contain less than 10 % of non-$\mathrm{t}\mathrm{t}\mathrm{H}$ events. However, in some cases such purities cannot be achieved for both production and decay modes.

Mixed production mode composition is common in $\mathrm{VBF}$-tagged event categories where the $\mathrm{g} \mathrm{g} \mathrm{H}$ contribution can be as high as 50 %, and in $\mathrm{V}\mathrm{H}$ tags where $\mathrm{W}\mathrm{H}$ and $\mathrm{Z}\mathrm{H}$ mixtures are common.

For decay modes, mixed composition is more marked for signatures involving light leptons and $E_{\mathrm{T}}^{\text {miss}}$, where both the $\mathrm{H} \rightarrow \mathrm{W}\mathrm{W}$ and $\mathrm{H} \rightarrow \tau \tau$ decays may contribute. This can be seen in Table 1, where some $\mathrm{V}\mathrm{H}$-tag analyses targeting $\mathrm{H} \rightarrow \mathrm{W}\mathrm{W}$ decays have a significant contribution from $\mathrm{H} \rightarrow \tau \tau$ decays and vice versa. This is also the case in the $\mathrm{e}\mathrm{\mu }$ channel in the $\mathrm{H} \rightarrow \tau \tau$ analysis, in particular in the 2-jet $\mathrm{VBF}$ tag categories, where the contribution from $\mathrm{H} \rightarrow \mathrm{W}\mathrm{W}$ decays is sizeable and concentrated at low values of $m_{\tau \tau }$, entailing a genuine sensitivity of these categories to $\mathrm{H} \rightarrow \mathrm{W}\mathrm{W}$ decays. On the other hand, in the $\mathrm{e}\mathrm{e}$ and $\mathrm{\mu }\mathrm{\mu }$ channels of the $\mathrm{H} \rightarrow \tau \tau$ analysis, the contribution from $\mathrm{H} \rightarrow \mathrm{W}\mathrm{W}$ is large when integrated over the full range of the MVA observable used, but given that the analysis is optimized for $\tau \tau$ decays the contribution from $\mathrm{H} \rightarrow \mathrm{W}\mathrm{W}$ is not concentrated in the regions with largest signal-to-background ratio, and provides little added sensitivity.

Another case of mixed decay mode composition is present in the analyses targeting $\mathrm{t}\mathrm{t}\mathrm{H}$ production, where the $\mathrm{H} \rightarrow \text {leptons}$ decay selection includes sizeable contributions from $\mathrm{H} \rightarrow \mathrm{W}\mathrm{W}$ and $\mathrm{H} \rightarrow \tau \tau$ decays, and to a lesser extent also from $\mathrm{H} \rightarrow \mathrm{Z}\mathrm{Z}$ decays. The mixed composition is a consequence of designing the analysis to have the highest possible sensitivity to the $\mathrm{t}\mathrm{t}\mathrm{H}$ production mode. The analysis of $\mathrm{t}\mathrm{t}\mathrm{H}$ with $\mathrm{H} \rightarrow \mathrm{\tau }_{\mathrm{h}} \mathrm{\tau }_{\mathrm{h}}$ decay has an expected signal composition that is dominated by $\mathrm{H} \rightarrow \tau \tau$ decays, followed by $\mathrm{H} \rightarrow \mathrm{W}\mathrm{W}$ decays, and a smaller contribution of $\mathrm{H} \rightarrow \mathrm{b} \mathrm{b}$ decays. Finally, in the analysis of $\mathrm{t}\mathrm{t}\mathrm{H}$ with $\mathrm{H} \rightarrow \mathrm{b} \mathrm{b}$, there is an event category of the $\text {lepton}+\text {jets}$ channel that requires six or more jets and two b-tagged jets where the signal composition is expected to be 58 % from $\mathrm{H} \rightarrow \mathrm{b} \mathrm{b}$ decays, 24 % from $\mathrm{H} \rightarrow \mathrm{W}\mathrm{W}$ decays, and the remaining 18 % from other SM decay modes; in the dilepton channel, the signal composition in the event category requiring four or more jets and two b-tagged jets is expected to be 45 % from $\mathrm{H} \rightarrow \mathrm{b} \mathrm{b}$ decays, 35 % from $\mathrm{H} \rightarrow \mathrm{W}\mathrm{W}$ decays, and 14 % from $\mathrm{H} \rightarrow \tau \tau$ decays.

When results are grouped according to the decay tag, each individual category is assigned to the decay mode group that, in the SM, is expected to dominate the sensitivity in that channel. In particular,

- $\mathrm{H} \rightarrow \gamma \gamma$ tagged includes only categories from the $\mathrm{H} \rightarrow \gamma \gamma$ analysis of Ref. [18].
- $\mathrm{H} \rightarrow \mathrm{Z}\mathrm{Z}$ tagged includes only categories from the $\mathrm{H} \rightarrow \mathrm{Z}\mathrm{Z}$ analysis of Ref. [16].
- $\mathrm{H} \rightarrow \mathrm{W}\mathrm{W}$ tagged includes all the channels from the $\mathrm{H} \rightarrow \mathrm{W}\mathrm{W}$ analysis of Ref. [22] and the channels from the analysis of $\mathrm{t}\mathrm{t}\mathrm{H}$ with $\mathrm{H} \rightarrow \text {leptons}$ of Ref. [29].
- $\mathrm{H} \rightarrow \tau \tau$ tagged includes all the channels from the $\mathrm{H} \rightarrow \tau \tau$ analysis of Ref. [23] and the channels from the analysis of $\mathrm{t}\mathrm{t}\mathrm{H}$ targeting $\mathrm{H} \rightarrow \mathrm{\tau }_{\mathrm{h}} \mathrm{\tau }_{\mathrm{h}}$ of Ref. [29].
- $\mathrm{H} \rightarrow \mathrm{b} \mathrm{b}$ tagged includes all the channels of the analysis of $\mathrm{V}\mathrm{H}$ with $\mathrm{H} \rightarrow \mathrm{b} \mathrm{b}$ of Ref. [21] and the channels from the analysis of $\mathrm{t}\mathrm{t}\mathrm{H}$ targeting $\mathrm{H} \rightarrow \mathrm{b} \mathrm{b}$ of Ref. [29].
- $\mathrm{H} \rightarrow \mu \mu$ tagged includes only categories from the $\mathrm{H} \rightarrow \mu \mu$ analysis of Ref. [30].

When results are grouped by the production tag, the same reasoning of assignment by preponderance of composition is followed, using the information in Table 1.

In the combined analyses, all contributions in a given production tag or decay mode group are considered as signal and scaled accordingly.

### Expected differences with respect to the results of input analyses

The grouping of channels described in Sect. 3.3 is among the reasons why the results of the combination may seem to differ from those of the individual published analyses. In addition, the combined analysis takes into account correlations among several sources of systematic uncertainty. Care is taken to understand the post-fit behaviour of the parameters that are correlated between analyses, both in terms of the post-fit parameter values and uncertainties. Finally, the combination is evaluated at a value of $m_\mathrm{{H}}$ that is not the value that was used in some of the individual published analyses, entailing changes to the expected production cross sections and branching fractions of the SM Higgs boson. Changes are sizeable in some cases:

- In Refs. [16, 22] the results for $\mathrm{H} \rightarrow \mathrm{Z}\mathrm{Z} \rightarrow 4\ell$ and $\mathrm{H} \rightarrow \mathrm{W}\mathrm{W} \rightarrow \ell \nu \ell \nu$ are evaluated for $m_\mathrm{{H}} =125.6\,\text {GeV}$, the mass measured in the $\mathrm{H} \rightarrow \mathrm{Z}\mathrm{Z} \rightarrow 4\ell$ analysis. In the present combination, the results are evaluated for $m_\mathrm{{H}} =125.0\,\text {GeV}$, the mass measured from the combined analysis of the $\mathrm{H} \rightarrow \gamma \gamma$ and $\mathrm{H} \rightarrow \mathrm{Z}\mathrm{Z} \rightarrow 4\ell$ measurements, presented in Sect. 4.1. For values of $m_\mathrm{{H}}$ in this region, the branching fractions for $\mathrm{H} \rightarrow \mathrm{Z}\mathrm{Z}$ and $\mathrm{H} \rightarrow \mathrm{W}\mathrm{W}$ vary rapidly with $m_\mathrm{{H}}$. For the change of $m_\mathrm{{H}}$ in question, $\mathcal {B}(\mathrm{H} \rightarrow \mathrm{Z}\mathrm{Z} ,m_\mathrm{{H}} =125.0\,\text {GeV} )/\mathcal {B}(\mathrm{H} \rightarrow \mathrm{Z}\mathrm{Z} ,m_\mathrm{{H}} =125.6\,\text {GeV})=0.95$ and $\mathcal {B}(\mathrm{H} \rightarrow \mathrm{W}\mathrm{W} ,m_\mathrm{{H}} =125.0\,\text {GeV} )/\mathcal {B}(\mathrm{H} \rightarrow \mathrm{W}\mathrm{W} ,m_\mathrm{{H}} =125.6\,\text {GeV})=0.96$ [171].
- The expected production cross sections for the SM Higgs boson depend on $m_\mathrm{{H}}$. For the change in $m_\mathrm{{H}}$ discussed above, the total production cross sections for 7 and 8$\,\text {TeV}$ collisions vary similarly: $\sigma _\text {tot}(m_\mathrm{{H}} =125.0\,\text {GeV} )/\sigma _\text {tot}(m_\mathrm{{H}} =125.6\,\text {GeV})\sim 1.01$. While the variation of the total production cross section is dominated by the $\mathrm{g} \mathrm{g} \mathrm{H}$ production process, the variation is about 1.005 for $\mathrm{VBF}$, around 1.016 for $\mathrm{V}\mathrm{H}$, and around 1.014 for $\mathrm{t}\mathrm{t}\mathrm{H}$  [171].
- The $\mathrm{H} \rightarrow \tau \tau$ analysis of Ref. [23] focused on exploring the coupling of the Higgs boson to the tau lepton. For this reason nearly all results in Ref. [23] were obtained by treating the $\mathrm{H} \rightarrow \mathrm{W}\mathrm{W}$ contribution as a background, set to the SM expectation. In the present combined analysis, both the $\mathrm{H} \rightarrow \tau \tau$ and $\mathrm{H} \rightarrow \mathrm{W}\mathrm{W}$ contributions are considered as signal in the $\tau \tau$ decay tag analysis. This treatment leads to an increased sensitivity to the presence of a Higgs boson that decays into both $\tau \tau$ and $\mathrm{W}\mathrm{W}$.
- The search for invisible Higgs decays of Ref. [28] includes a modest contribution to the sensitivity from the analysis targeting $\mathrm{Z}\mathrm{H}$ production with $\mathrm{Z}\rightarrow \mathrm{b} \mathrm{b}$ decays. The events selected by that analysis overlap with those of the analysis of $\mathrm{V}\mathrm{H}$ production with $\mathrm{H} \rightarrow \mathrm{b} \mathrm{b}$ decays, and are therefore not considered in this combination. Given the limited sensitivity of that search, the overall sensitivity to invisible decays is not significantly impacted.
- The contribution from the $\mathrm{g} \mathrm{g} \rightarrow \mathrm{Z}\mathrm{H}$ process was not included in Ref. [21] as calculations for the cross section as a function of $p_{\mathrm{T}} (\mathrm{Z})$ were not available. Since then, the search for $\mathrm{V}\mathrm{H}$ production with $\mathrm{H} \rightarrow \mathrm{b} \mathrm{b}$ has been augmented by the use of recent NLO calculations for the $\mathrm{g} \mathrm{g} \rightarrow \mathrm{Z}\mathrm{H}$ contribution [176–179]. In the $\mathrm{Z}(\nu \nu )\mathrm{H} (\mathrm{b} \mathrm{b} )$ and $\mathrm{Z}(\ell \ell )\mathrm{H} (\mathrm{b} \mathrm{b} )$ channels, the addition of this process leads to an increase of the expected signal yields by 10 % to 30 % for $p_{\mathrm{T}} (\mathrm{Z})$ around and above 150$\,\text {GeV}$. When combined with the unchanged $\mathrm{W}\mathrm{H}$ channels, the overall expected sensitivity for $\mathrm{V}\mathrm{H}$ production with $\mathrm{H} \rightarrow \mathrm{b} \mathrm{b}$ increases by about 10 %.

In all analyses used, the contribution from associated production of a Higgs boson with a bottom quark pair, $\mathrm{b}\mathrm{b}\mathrm{H}$, is neglected; in inclusive selections this contribution is much smaller than the uncertainties in the gluon fusion production process, whereas in exclusive categories it has been found that the jets associated with the bottom quarks are so soft that the efficiency to select such events is low enough and no sensitivity is lost. In the future, with more data, it may be possible to devise experimental selections that permit the study of the $\mathrm{b}\mathrm{b}\mathrm{H}$ production mode as predicted by the SM.

## Mass measurement and direct limits on the natural width

In this section we first present a measurement of the mass of the new boson from the combined analysis of the high-resolution $\mathrm{H} \rightarrow \gamma \gamma$ and $\mathrm{H} \rightarrow \mathrm{Z}\mathrm{Z} \rightarrow 4\ell$ channels. We then proceed to set direct limits on its natural width.

### Mass of the observed state

**Fig. 1:**
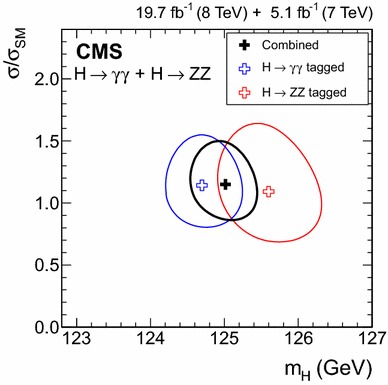
The 68 % CL confidence regions for the signal strength $\sigma / \sigma _{\text {SM}}$ versus the mass of the boson ${m}_\mathrm{{H}}$ for the $\mathrm{H} \rightarrow \gamma \gamma$ and $\mathrm{H} \rightarrow \mathrm{Z}\mathrm{Z} \rightarrow 4\ell$ final states, and their combination. The symbol $\sigma / \sigma _{\text {SM}}$ denotes the production cross section times the relevant branching fractions, relative to the SM expectation. In this combination, the relative signal strength for the two decay modes is set to the expectation for the SM Higgs boson

Figure 1 shows the 68 % CL confidence regions for two parameters of interest, the signal strength relative to the SM expectation, $\mu =\sigma /\sigma _\text {SM}$, and the mass, $m_{\mathrm{{H}}}$, obtained from the $\mathrm{H} \rightarrow \mathrm{Z}\mathrm{Z} \rightarrow 4\ell$ and $\gamma \gamma$ channels, which have excellent mass resolution. The combined 68 % CL confidence region, bounded by a black curve in Fig. 1, is calculated assuming the relative event yield between the two channels as predicted by the SM, while the overall signal strength is left as a free parameter.

**Fig. 2:**
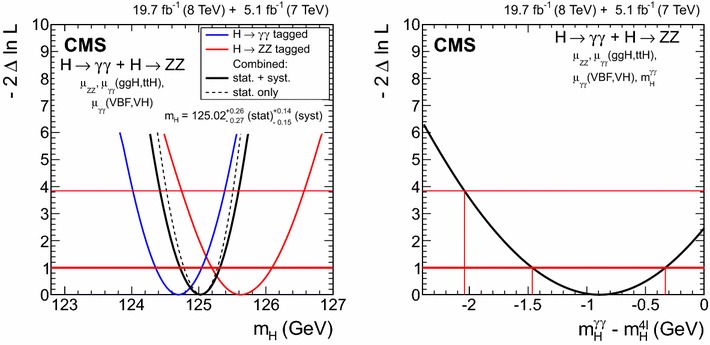
(*Left*) Scan of the test statistic $q(m_{\mathrm{{H}}})=-2 \Delta \ln \mathcal {L}$ versus the mass of the boson $m_{\mathrm{{H}}}$ for the $\mathrm{H} \rightarrow \gamma \gamma$ and $\mathrm{H} \rightarrow \mathrm{Z}\mathrm{Z} \rightarrow 4\ell$ final states separately and for their combination. Three independent signal strengths, $(\mathrm{g} \mathrm{g} \mathrm{H} ,\mathrm{t}\mathrm{t}\mathrm{H} )\rightarrow \gamma \gamma$, $(\mathrm{VBF},\mathrm{V}\mathrm{H} )\rightarrow \gamma \gamma$, and $\text {pp}\rightarrow \mathrm{H} \rightarrow \mathrm{Z}\mathrm{Z} \rightarrow 4\ell$, are profiled together with all other nuisance parameters. (*Right*) Scan of the test statistic $q(m_\mathrm{{H}}^{\gamma \gamma } - m_\mathrm{{H}}^{4\ell })$ versus the difference between two individual mass measurements for the same model of signal strengths used in the *left panel*

To extract the value of $m_{\mathrm{{H}}}$ in a way that is not completely dependent on the SM prediction for the production and decay ratios, the signal strength modifiers for the $(\mathrm{g} \mathrm{g} \mathrm{H} ,\mathrm{t}\mathrm{t}\mathrm{H} )\rightarrow \gamma \gamma$, $(\mathrm{VBF},\mathrm{V}\mathrm{H} )\rightarrow \gamma \gamma$, and $\text {pp}\rightarrow \mathrm{H} \rightarrow \mathrm{Z}\mathrm{Z} \rightarrow 4\ell$ processes are taken as independent, unconstrained, parameters. The signal in all channels is assumed to be due to a single state with mass $m_{\mathrm{{H}}}$. The best-fit value of $m_{\mathrm{{H}}}$ and its uncertainty are extracted from a scan of the combined test statistic $q(m_{\mathrm{{H}}})$ with the three signal strength modifiers profiled together with all other nuisance parameters; i.e. the signal strength modifiers float freely in the fits performed to scan $q(m_{\mathrm{{H}}})$. Figure 2 (left) shows the scan of the test statistic as a function of the mass $m_{\mathrm{{H}}}$ separately for the $\mathrm{H} \rightarrow \gamma \gamma$ and $\mathrm{H} \rightarrow \mathrm{Z}\mathrm{Z} \rightarrow 4\ell$ channels, and for their combination. The intersections of the $q(m_{\mathrm{{H}}})$ curves with the thick horizontal line at 1.00 and thin line at 3.84 define the 68 % and 95 % CL confidence intervals for the mass of the observed particle, respectively. These intervals include both the statistical and systematic uncertainties. The mass is measured to be $m_{\mathrm{{H}}} = 125.02 ^{+0.29}_{-0.31} \,\text {GeV}$. The less precise evaluations from the $\mathrm{H} \rightarrow \mathrm{W}\mathrm{W}$ analysis [22], $m_{\mathrm{{H}}} =128^{+7}_{-5}\,\text {GeV}$, and from the $\mathrm{H} \rightarrow \tau \tau$ analysis [23], $m_{\mathrm{{H}}} =122\pm 7\,\text {GeV}$, are compatible with this result.

To evaluate the statistical component of the overall uncertainty, we also perform a scan of $$q(m_{\mathrm{{H}}})$$ fixing all nuisance parameters to their best-fit values, except those related to the $$\mathrm{H} \rightarrow \gamma \gamma $$ background models; given that the $$\mathrm{H} \rightarrow \gamma \gamma $$ background distributions are modelled from fits to data, their degrees of freedom encode fluctuations which are statistical in nature. The result is shown by the dashed curve in Fig. 2 (left). The crossings of the dashed curve with the thick horizontal line define the 68 % CL confidence interval for the statistical uncertainty in the mass measurement: $$^{+0.26}_{-0.27}$$$$\,\text {GeV}$$. We derive the systematic uncertainty assuming that the total uncertainty is the sum in quadrature of the statistical and systematic components; the full result is $$m_{\mathrm{{H}}} = 125.02\,^{+0.26}_{-0.27} \,\text {(stat)} \,^{+0.14}_{-0.15} \,\text {(syst)} \,\text {GeV} $$. The median expected uncertainty is evaluated using an Asimov pseudo-data sample [182] constructed from the best-fit values obtained when testing for the compatibility of the mass measurement in the $$\mathrm{H} \rightarrow \gamma \gamma $$ and $$\mathrm{H} \rightarrow \mathrm{Z}\mathrm{Z} \rightarrow 4\ell $$ channels. The expected uncertainty thus derived is $$\,^{+0.26}_{-0.25}\,\text {(stat)} \,\pm 0.14\,\text {(syst)} \,\text {GeV} $$, in good agreement with the observation in data. As a comparison, the median expected uncertainty is also derived by constructing an Asimov pseudo-data sample as above except that the signal strength modifiers are set to unity (as expected in the SM) and $$m_\mathrm{{H}}^{\gamma \gamma }=m_\mathrm{{H}}^{4\ell }=125\,\text {GeV} $$, leading to an expected uncertainty of $$\,\pm 0.28\,\text {(stat)} \,\pm 0.13\,\text {(syst)} \,\text {GeV} $$. As could be anticipated, the statistical uncertainty is slightly larger given that the observed signal strength in the $$\mathrm{H} \rightarrow \gamma \gamma $$ channel is larger than unity, and the systematic uncertainty is slightly smaller given the small mass difference between the two channels that is observed in data.

To quantify the compatibility of the $\mathrm{H} \rightarrow \gamma \gamma$ and $\mathrm{H} \rightarrow \mathrm{Z}\mathrm{Z}$ mass measurements with each other, we perform a scan of the test statistic $q(m_\mathrm{{H}}^{\gamma \gamma } - m_\mathrm{{H}}^{4\ell })$, as a function of the difference between the two mass measurements. Besides the three signal strength modifiers, there are two additional parameters in this test: the mass difference and $m_\mathrm{{H}}^{\gamma \gamma }$. In the scan, the three signal strengths and $m_\mathrm{{H}}^{\gamma \gamma }$ are profiled together with all nuisance parameters. The result from the scan shown in Fig. 2 (right) is $m_\mathrm{{H}}^{\gamma \gamma } - m_\mathrm{{H}}^{4\ell }=-0.89^{+0.56}_{-0.57}\,\text {GeV}$. From evaluating $q(m_\mathrm{{H}}^{\gamma \gamma } - m_\mathrm{{H}}^{4\ell }=0)$ it can be concluded that the mass measurements in $\mathrm{H} \rightarrow \gamma \gamma$ and $\mathrm{H} \rightarrow \mathrm{Z}\mathrm{Z} \rightarrow 4\ell$ agree at the $1.6\sigma$ level.

To assess the dependency of the result on the SM Higgs boson hypothesis, the measurement of the mass is repeated using the same channels, but with the following two sets of assumptions: (i) allowing a common signal strength modifier to float, which corresponds to the result in Fig. 1, and (ii) constraining the relative production cross sections and branching fractions to the SM predictions, i.e. $\mu =1$. The results from these two alternative measurements differ by less than 0.1$\,\text {GeV}$ from the main result, both in terms of the best-fit value and the uncertainties.

### Direct limits on the width of the observed state

For $$m_\mathrm{{H}} \sim 125\,\text {GeV} $$ the SM Higgs boson is predicted to be narrow, with a total width $$\varGamma _{\mathrm{SM}} \sim 4\,\text {MeV} $$. From the study of off-shell Higgs boson production, CMS has previously set an indirect limit on the total width, $$\varGamma _{\text {tot}}/\varGamma _{\mathrm{SM}} < 5.4$$$$(8.0)$$ observed (expected) at the 95 % CL [27]. While that result is about two orders of magnitude better than the experimental mass resolution, it relies on assumptions on the underlying theory, such as the absence of contributions to Higgs boson off-shell production from particles beyond the standard model. In contrast, a direct limit does not rely on such assumptions and is only limited by the experimental resolution.

The best experimental mass resolution, achieved in the $\mathrm{H} \rightarrow \gamma \gamma$ and $\mathrm{H} \rightarrow \mathrm{Z}\mathrm{Z} \rightarrow 4\ell$ analyses, is typically between 1$\,\text {GeV}$ and 3$\,\text {GeV}$, as shown in Table 1. The resolution depends on the energy, rapidity, and azimuthal angle of the decay products, and on the flavour of the leptons in the case of the $\mathrm{H} \rightarrow \mathrm{Z}\mathrm{Z} \rightarrow 4\ell$ decay. If found inconsistent with the expected detector resolution, the total width measured in data could suggest the production of a resonance with a greater intrinsic width or the production of two quasi-degenerate states.

**Fig. 3:**
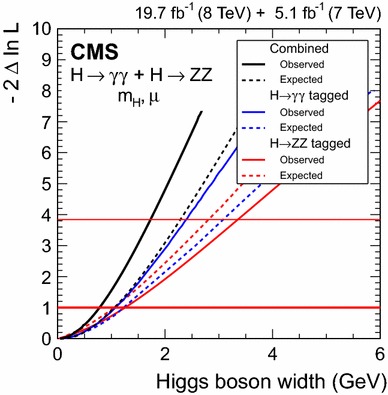
Likelihood scan as a function of the width of the boson. The *continuous (dashed) lines* show the observed (expected) results for the $\mathrm{H} \rightarrow \gamma \gamma$ analysis, the $\mathrm{H} \rightarrow \mathrm{Z}\mathrm{Z} \rightarrow 4\ell$ analysis, and their combination. The data are consistent with $\varGamma _{\mathrm{SM}} \sim 4\,\text {MeV}$ and for the combination of the two channels the observed (expected) upper limit on the width at the 95 % CL is 1.7 (2.3)$\,\text {GeV}$

To perform this measurement the signal models in the $\mathrm{H} \rightarrow \gamma \gamma$ and $\mathrm{H} \rightarrow \mathrm{Z}\mathrm{Z} \rightarrow 4\ell$ analyses allow for a natural width using the relativistic Breit–Wigner distribution, as described in Refs. [16, 18]. Figure 3 shows the likelihood scan as a function of the assumed natural width. The mass of the boson and a common signal strength are profiled along with all other nuisance parameters. The dashed lines show the expected results for the SM Higgs boson. For the $\mathrm{H} \rightarrow \gamma \gamma$ channel the observed (expected) upper limit at the 95 % CL is 2.4 (3.1)$\,\text {GeV}$. For the $\mathrm{H} \rightarrow \mathrm{Z}\mathrm{Z} \rightarrow 4\ell$ channel the observed (expected) upper limit at the 95 % CL is 3.4 (2.8)$\,\text {GeV}$. For the combination of the two analyses, the observed (expected) upper limit at the 95 % CL is 1.7 (2.3)$\,\text {GeV}$.

## Significance of the observations in data

This section provides an assessment of the significance of the observed excesses at the best-fit mass value, $m_\mathrm{{H}} =125.0\,\text {GeV}$.

Table 2 summarizes the median expected and observed local significance for a SM Higgs boson mass of $125.0\,\text {GeV}$ from the different decay mode tags, grouped as described in Sect. 3.3. The value of $m_{\mathrm{{H}}}$ is fixed to the best-fit combined measurement presented in Sect. 4.1. The values of the expected significance are evaluated using the post-fit expected background rates and the signal rates expected from the SM. In the three diboson decay mode tags, the significance is close to, or above, $5\sigma$. In the $\tau \tau$ decay mode tag the significance is above $3\sigma$.

**Table 2:** The observed and median expected significances of the excesses for each decay mode group, assuming $m_{\mathrm{{H}}} =125.0\,\text {GeV}$. The channels are grouped by decay mode tag as described in Sect. 3.3; when there is a difference in the channels included with respect to the published results for the individual channels, the result for the grouping used in those publications is also given

Channel grouping	Significance ($\sigma$)	
	Observed	Expected
$\mathrm{H} \rightarrow \mathrm{Z}\mathrm{Z}$ tagged	6.5	6.3
$\mathrm{H} \rightarrow \gamma \gamma$ tagged	5.6	5.3
$\mathrm{H} \rightarrow \mathrm{W}\mathrm{W}$ tagged	4.7	5.4
*Grouped as in Ref.* [22]	*4.3*	*5.4*
$\mathrm{H} \rightarrow \tau \tau$ tagged	3.8	3.9
*Grouped as in Ref.* [23]	*3.9*	*3.9*
$\mathrm{H} \rightarrow \mathrm{b} \mathrm{b}$ tagged	2.0	2.6
*Grouped as in Ref.* [21]	*2.1*	*2.5*
$\mathrm{H} \rightarrow \mu \mu$ tagged	$<$0.1	0.4

Differences between the results in Table 2 and the individual publications are understood in terms of the discussion in Sects. 3.3 and 3.4, namely the grouping of channels by decay mode tag, the change of the $m_\mathrm{{H}}$ value at which the significance of the $\mathrm{H} \rightarrow \mathrm{Z}\mathrm{Z} \rightarrow 4\ell$ and $\mathrm{H} \rightarrow \mathrm{W}\mathrm{W}$ analyses is evaluated, and the treatment of $\mathrm{H} \rightarrow \mathrm{W}\mathrm{W}$ as part of the signal, instead of background, in the $\mathrm{H} \rightarrow \tau \tau$ analysis.

Finally, the observation of the $\mathrm{H} \rightarrow \gamma \gamma$ and $\mathrm{H} \rightarrow \mathrm{Z}\mathrm{Z} \rightarrow 4\ell$ decay modes indicates that the new particle is a boson, and the diphoton decay implies that its spin is different from unity [190, 191]. Other observations, beyond the scope of this paper, disfavour spin-1 and spin-2 hypotheses and, assuming that the boson has zero spin, are consistent with the pure scalar hypothesis, while disfavouring the pure pseudoscalar hypothesis [16, 22, 41].

## Compatibility of the observed yields with the SM Higgs boson hypothesis

The results presented in this section focus on the Higgs boson production and decay modes, which can be factorized under the narrow-width approximation, leading to $N_{ij}\sim \sigma _{i}\;\mathcal {B}_{j}$, where $N_{ij}$ represents the event yield for the combination of production mode $i$ and decay mode $j$, $\sigma _{i}$ is the production cross section for production process $i$, and $\mathcal {B}_{j}$ is the branching fraction into decay mode $j$. Studies where the production and decay modes are interpreted in terms of underlying couplings of particles to the Higgs boson are presented in Sect. 7.

The size of the current data set permits many compatibility tests between the observed excesses and the expected SM Higgs boson signal. These compatibility tests do not constitute measurements of any physics parameters per se, but rather allow one to probe for deviations of the various observations from the SM expectations. The tests evaluate the compatibility of the data observed in the different channels with the expectations for the SM Higgs boson with a mass equal to the best-fit value found in Sect. 4.1, $m_{\mathrm{{H}}} =125.0\,\text {GeV}$.

This section is organized by increasing degree of complexity of the deviations being probed. In Sect. 6.1 we assess the compatibility of the overall signal strength for all channels combined with the SM Higgs hypothesis. In Sect. 6.2 the compatibility is assessed by production tag group, decay tag group, and production and decay tag group. We then turn to the study of production modes. Using the detailed information on the expected SM Higgs production contributions, Sect. 6.3 discusses, for each decay tag group, the results of considering two signal strengths, one scaling the $\mathrm{g} \mathrm{g} \mathrm{H}$ and $\mathrm{t}\mathrm{t}\mathrm{H}$ contributions, and the other scaling the $\mathrm{VBF}$ and $\mathrm{V}\mathrm{H}$ contributions. Then, assuming the expected relative SM Higgs branching fractions, Sect. 6.4 provides a combined analysis for signal strengths scaling the $\mathrm{g} \mathrm{g} \mathrm{H}$, $\mathrm{VBF}$, $\mathrm{V}\mathrm{H}$, and $\mathrm{t}\mathrm{t}\mathrm{H}$ contributions individually. Turning to the decay modes, Sect. 6.5 performs combined analyses of signal strength ratios between different decay modes, where some uncertainties from theory and some experimental uncertainties cancel out. Finally, using the structure of the matrix of production and decay mode signal strengths, Sect. 6.6 tests for the possibility that the observations are due to the presence of more than one state degenerate in mass.

### Overall signal strength

**Fig. 4:**
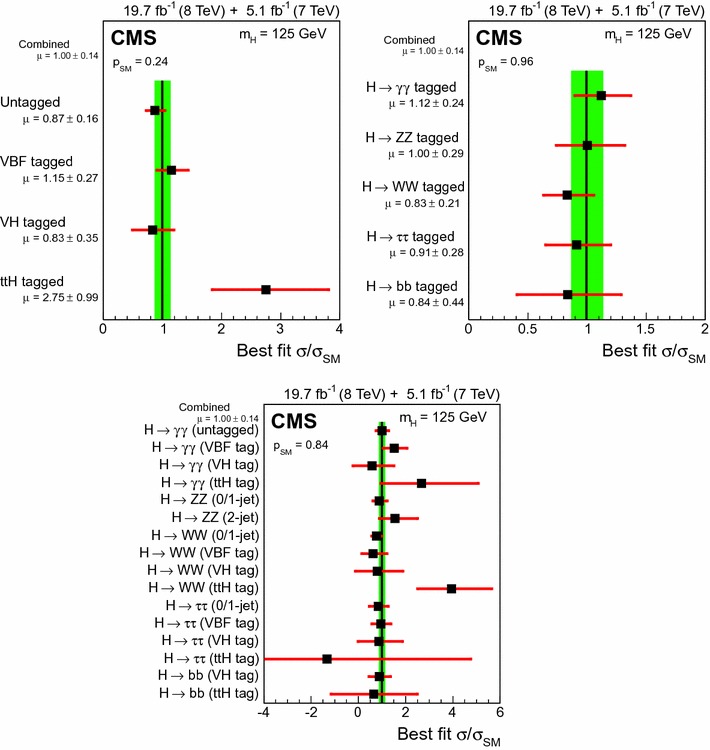
Values of the best-fit $\sigma / \sigma _\text {SM}$ for the overall combined analysis (*solid vertical line*) and separate combinations grouped by production mode tag, predominant decay mode, or both. The $\sigma / \sigma _{\text {SM}}$ ratio denotes the production cross section times the relevant branching fractions, relative to the SM expectation. The *vertical band* shows the overall $\sigma / \sigma _\text {SM}$ uncertainty. The *horizontal bars* indicate the $\pm 1$ standard deviation uncertainties in the best-fit $\sigma / \sigma _\text {SM}$ values for the individual combinations; these bars include both statistical and systematic uncertainties. (*Top left*) Combinations grouped by analysis tags targeting individual production mechanisms; the excess in the $\mathrm{t}\mathrm{t}\mathrm{H}$-tagged combination is largely driven by the $\mathrm{t}\mathrm{t}\mathrm{H}$-tagged $\mathrm{H} \rightarrow \gamma \gamma$ and $\mathrm{H} \rightarrow \mathrm{W}\mathrm{W}$ channels as can be seen in the bottom panel. (*Top right*) Combinations grouped by predominant decay mode. (*Bottom*) Combinations grouped by predominant decay mode and additional tags targeting a particular production mechanism

The best-fit value for the common signal strength modifier $\hat{\mu }= \hat{\sigma }/ \sigma _{\text {SM}}$, obtained from the combined analysis of all channels, provides the simplest compatibility test. In the formal fit, $\hat{\mu }$ is allowed to become negative if the observed number of events is smaller than the expected yield for the background-only hypothesis. The observed $\hat{\mu }$, assuming $m_\mathrm{{H}} =125.0\,\text {GeV}$, is $1.00 ^{+0.14}_{-0.13}$, consistent with unity, the expectation for the SM Higgs boson. This value is shown as the vertical bands in the three panels of Fig. 4.

The total uncertainty can be broken down into a statistical component (stat); a component associated with the uncertainties related to renormalization and factorization scale variations, parton distribution functions, branching fractions, and underlying event description (theo); and any other systematic uncertainties (syst). The result is $1.00\,\pm 0.09\,\text {(stat)} \,^{+0.08}_{-0.07}\,\text {(theo)} \,\pm 0.07\,\text {(syst)}$. Evolution of the SM predictions may not only reduce the associated uncertainties from theory, but also change the central value given above.

### Grouping by predominant decay mode and/or production tag

One step in going beyond a single signal strength modifier is to evaluate the signal strength in groups of channels from different analyses. The groups chosen reflect the different production tags, predominant decay modes, or both. Once the fits for each group are performed, a simultaneous fit to all groups is also performed to assess the compatibility of the results with the SM Higgs boson hypothesis.

Figure 4 shows the $\hat{\mu }$ values obtained in different independent combinations of channels for $m_\mathrm{{H}} = 125.0\,\text {GeV}$, grouped by additional tags targeting events from particular production mechanisms, by predominant decay mode, or both. As discussed in Sect. 3.3, the expected purities of the different tagged samples vary substantially. Therefore, these plots cannot be interpreted as compatibility tests for pure production mechanisms or decay modes, which are studied in Sect. 6.4.

For each type of grouping, the level of compatibility with the SM Higgs boson cross section can be quantified by the value of the test statistic function of the signal strength parameters simultaneously fitted for the $N$ channels considered in the group, $\mu _1, \mu _2,\ldots ,\mu _N$,

5 $$\begin{aligned} q_{\mu } = -2 \Delta \ln \mathcal {L} = -2 \ln \frac{ \mathcal {L}(\text {data} \, | \, \mu _{i}, \hat{\theta }_{\mu _{i}}) }{\mathcal {L}(\text {data} \, | \, \hat{\mu }_{i}, \hat{\theta })} \end{aligned}$$

evaluated for $\mu _1 = \mu _2 =\cdots =\mu _N = 1$. For each type of grouping, the corresponding $q_{\mu }(\mu _1=\mu _2=\cdots =\mu _N=1)$ from the simultaneous fit of $N$ signal strength parameters is expected to behave asymptotically as a $\chi ^{2}$ distribution with $N$ degrees of freedom (dof).

The results for the four independent combinations grouped by production mode tag are depicted in Fig. 4 (top left). An excess can be seen for the $\mathrm{t}\mathrm{t}\mathrm{H}$-tagged combination, due to the observations in the $\mathrm{t}\mathrm{t}\mathrm{H}$-tagged $\mathrm{H} \rightarrow \gamma \gamma$ and $\mathrm{H} \rightarrow \text {leptons}$ analyses that can be appreciated from the bottom panel. The simultaneous fit of the signal strengths for each group of production process tags results in $\chi ^{2}/\text {dof} = 5.5 / 4$ and an asymptotic $p\text {-value}$ of $0.24$, driven by the excess observed in the group of analyses tagging the $\mathrm{t}\mathrm{t}\mathrm{H}$ production process.

The results for the five independent combinations grouped by predominant decay mode are shown in Fig. 4 (top right). The simultaneous fit of the corresponding five signal strengths yields $\chi ^{2}/\text {dof} = 1.0 / 5$ and an asymptotic $p\text {-value}$ of $0.96$.

The results for sixteen individual combinations grouped by production tag and predominant decay mode are shown in Fig. 4 (bottom). The simultaneous fit of the corresponding signal strengths gives a $\chi ^{2}/\text {dof} = 10.5 / 16$, which corresponds to an asymptotic $p\text {-value}$ of $0.84$.

The $p\text {-values}$ above indicate that these different ways of splitting the overall signal strength into groups related to the production mode tag, decay mode tag, or both, all yield results compatible with the SM prediction for the Higgs boson, $\mu =\mu _i=1$. The result of the $\mathrm{t}\mathrm{t}\mathrm{H}$-tagged combination is compatible with the SM hypothesis at the $2.0\sigma$ level.

### Fermion- and boson-mediated production processes and their ratio

The four main Higgs boson production mechanisms can be associated with either couplings of the Higgs boson to fermions ($\mathrm{g} \mathrm{g} \mathrm{H}$ and $\mathrm{t}\mathrm{t}\mathrm{H}$) or vector bosons ($\mathrm{VBF}$ and $\mathrm{V}\mathrm{H}$). Therefore, a combination of channels associated with a particular decay mode tag, but explicitly targeting different production mechanisms, can be used to test the relative strengths of the couplings to the vector bosons and fermions, mainly the top quark, given its importance in $\mathrm{g} \mathrm{g} \mathrm{H}$ production. The categorization of the different channels into production mode tags is not pure. Contributions from the different signal processes, evaluated from Monte Carlo simulation and shown in Table 1, are taken into account in the fits, including theory and experimental uncertainties; the factors used to scale the expected contributions from the different production modes are shown in Table 3 and do not depend on the decay mode. For a given decay mode, identical deviations of $\mu _{\mathrm{VBF},\mathrm{V}\mathrm{H} }$ and $\mu _\mathrm{{g} \mathrm{g} \mathrm{H} ,\mathrm{t}\mathrm{t}\mathrm{H} }$ from unity may also be due to a departure of the decay partial width from the SM expectation.

**Table 3:** Parameterization used to scale the expected SM Higgs boson yields from the different production modes when obtaining the results presented in Table 5 and Fig. 5 (left). The signal strength modifiers $\mu _\mathrm{{g} \mathrm{g} \mathrm{H} ,\mathrm{t}\mathrm{t}\mathrm{H} }$ and $\mu _{\mathrm{VBF},\mathrm{V}\mathrm{H} }$, common to all decay modes, are associated with the $\mathrm{g} \mathrm{g} \mathrm{H}$ and $\mathrm{t}\mathrm{t}\mathrm{H}$ and with the $\mathrm{VBF}$ and $\mathrm{V}\mathrm{H}$ production mechanisms, respectively

Parameters of interest: $\mu _\mathrm{{g} \mathrm{g} \mathrm{H} ,\mathrm{t}\mathrm{t}\mathrm{H} }$ and $\mu _{\mathrm{VBF},\mathrm{V}\mathrm{H} }$					
Signal model	$\mathrm{H} \rightarrow \gamma \gamma$	$\mathrm{H} \rightarrow \mathrm{Z}\mathrm{Z}$	$\mathrm{H} \rightarrow \mathrm{W}\mathrm{W}$	$\mathrm{H} \rightarrow \tau \tau$	$\mathrm{H} \rightarrow \mathrm{b} \mathrm{b}$
$\mathrm{g} \mathrm{g} \mathrm{H}$	$\mu _\mathrm{{g} \mathrm{g} \mathrm{H} ,\mathrm{t}\mathrm{t}\mathrm{H} }$	$\mu _\mathrm{{g} \mathrm{g} \mathrm{H} ,\mathrm{t}\mathrm{t}\mathrm{H} }$	$\mu _\mathrm{{g} \mathrm{g} \mathrm{H} ,\mathrm{t}\mathrm{t}\mathrm{H} }$	$\mu _\mathrm{{g} \mathrm{g} \mathrm{H} ,\mathrm{t}\mathrm{t}\mathrm{H} }$	$\mu _\mathrm{{g} \mathrm{g} \mathrm{H} ,\mathrm{t}\mathrm{t}\mathrm{H} }$
$\mathrm{VBF}$	$\mu _{\mathrm{VBF},\mathrm{V}\mathrm{H} }$	$\mu _{\mathrm{VBF},\mathrm{V}\mathrm{H} }$	$\mu _{\mathrm{VBF},\mathrm{V}\mathrm{H} }$	$\mu _{\mathrm{VBF},\mathrm{V}\mathrm{H} }$	$\mu _{\mathrm{VBF},\mathrm{V}\mathrm{H} }$
$\mathrm{V}\mathrm{H}$	$\mu _{\mathrm{VBF},\mathrm{V}\mathrm{H} }$	$\mu _{\mathrm{VBF},\mathrm{V}\mathrm{H} }$	$\mu _{\mathrm{VBF},\mathrm{V}\mathrm{H} }$	$\mu _{\mathrm{VBF},\mathrm{V}\mathrm{H} }$	$\mu _{\mathrm{VBF},\mathrm{V}\mathrm{H} }$
$\mathrm{t}\mathrm{t}\mathrm{H}$	$\mu _\mathrm{{g} \mathrm{g} \mathrm{H} ,\mathrm{t}\mathrm{t}\mathrm{H} }$	$\mu _\mathrm{{g} \mathrm{g} \mathrm{H} ,\mathrm{t}\mathrm{t}\mathrm{H} }$	$\mu _\mathrm{{g} \mathrm{g} \mathrm{H} ,\mathrm{t}\mathrm{t}\mathrm{H} }$	$\mu _\mathrm{{g} \mathrm{g} \mathrm{H} ,\mathrm{t}\mathrm{t}\mathrm{H} }$	$\mu _\mathrm{{g} \mathrm{g} \mathrm{H} ,\mathrm{t}\mathrm{t}\mathrm{H} }$

**Fig. 5:**
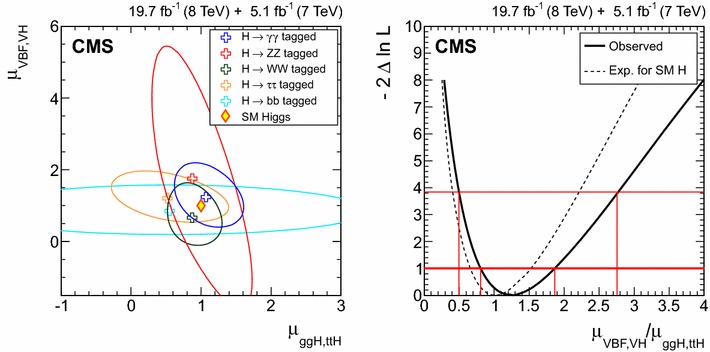
(*Left*) The 68 % CL confidence regions (bounded by the *solid curves*) for the signal strength of the $\mathrm{g} \mathrm{g} \mathrm{H}$ and $\mathrm{t}\mathrm{t}\mathrm{H}$ and of the $\mathrm{VBF}$ and $\mathrm{V}\mathrm{H}$ production mechanisms, $\mu _\mathrm{{g} \mathrm{g} \mathrm{H} ,\mathrm{t}\mathrm{t}\mathrm{H} }$ and $\mu _{\mathrm{VBF},\mathrm{V}\mathrm{H} }$, respectively. The *crosses* indicate the best-fit values obtained in each group of predominant decay modes: $\gamma \gamma$, $\mathrm{Z}\mathrm{Z}$, $\mathrm{W}\mathrm{W}$, $\tau \tau$, and $\mathrm{b} \mathrm{b}$. The *diamond* at $(1,1)$ indicates the expected values for the SM Higgs boson. (*Right*) Likelihood scan versus the ratio $\mu _{\mathrm{VBF},\mathrm{V}\mathrm{H} }/\mu _\mathrm{{g} \mathrm{g} \mathrm{H} ,\mathrm{t}\mathrm{t}\mathrm{H} }$, combined for all channels. The fit for $\mu _{\mathrm{VBF},\mathrm{V}\mathrm{H} }/\mu _\mathrm{{g} \mathrm{g} \mathrm{H} ,\mathrm{t}\mathrm{t}\mathrm{H} }$ is performed while profiling the five $\mu _\mathrm{{g} \mathrm{g} \mathrm{H} ,\mathrm{t}\mathrm{t}\mathrm{H} }$ parameters, one per visible decay mode, as shown in Table 4. The *solid curve* represents the observed result in data while the *dashed curve* indicates the expected median result in the presence of the SM Higgs boson. Crossings with the *horizontal thick* and *thin lines* denote the 68 % CL and 95 % CL confidence intervals, respectively

Figure 5 (left) shows the 68 % CL confidence regions for the signal strength modifiers associated with the $\mathrm{g} \mathrm{g} \mathrm{H}$ and $\mathrm{t}\mathrm{t}\mathrm{H}$ and with the $\mathrm{VBF}$ and $\mathrm{V}\mathrm{H}$ production mechanisms, $\mu _\mathrm{{g} \mathrm{g} \mathrm{H} ,\mathrm{t}\mathrm{t}\mathrm{H} }$ and $\mu _{\mathrm{VBF},\mathrm{V}\mathrm{H} }$, respectively. The five sets of contours correspond to the five predominant decay mode groups, introduced in Sect. 3.3. It can be seen in Fig. 5 (left) how the analyses in the $\mathrm{H} \rightarrow \mathrm{b} \mathrm{b}$ decay group constrain $\mu _{\mathrm{VBF},\mathrm{V}\mathrm{H} }$ more than $\mu _\mathrm{{g} \mathrm{g} \mathrm{H} ,\mathrm{t}\mathrm{t}\mathrm{H} }$, reflecting the larger sensitivity of the analysis of $\mathrm{V}\mathrm{H}$ production with $\mathrm{H} \rightarrow \mathrm{b} \mathrm{b}$ with respect to the analysis of $\mathrm{t}\mathrm{t}\mathrm{H}$ production with $\mathrm{H} \rightarrow \mathrm{b} \mathrm{b}$. An almost complementary situation can be found for the $\mathrm{H} \rightarrow \mathrm{Z}\mathrm{Z}$ analysis, where the data constrain $\mu _\mathrm{{g} \mathrm{g} \mathrm{H} ,\mathrm{t}\mathrm{t}\mathrm{H} }$ better than $\mu _{\mathrm{VBF},\mathrm{V}\mathrm{H} }$, reflecting the fact that the analysis is more sensitive to $\mathrm{g} \mathrm{g} \mathrm{H}$, the most abundant production mode. The SM Higgs boson expectation of $(1,1)$ is within the 68 % CL confidence regions for all predominant decay groups. The best-fit values for each decay tag group are given in Table 5.

The ratio of $\mu _{\mathrm{VBF},\mathrm{V}\mathrm{H} }$ and $\mu _\mathrm{{g} \mathrm{g} \mathrm{H} ,\mathrm{t}\mathrm{t}\mathrm{H} }$ provides a compatibility check with the SM Higgs boson expectation that can be combined across all decay modes. To perform the measurement of $\mu _{\mathrm{VBF},\mathrm{V}\mathrm{H} }/\mu _\mathrm{{g} \mathrm{g} \mathrm{H} ,\mathrm{t}\mathrm{t}\mathrm{H} }$, the SM Higgs boson signal yields in the different production processes and decay modes are parameterized according to the scaling factors presented in Table 4. The fit is performed simultaneously in all channels of all analyses and takes into account, within each channel, the full detail of the expected SM Higgs contributions from the different production processes and decay modes.

**Table 4:** Parameterization used to scale the expected SM Higgs boson yields for the different production processes and decay modes when obtaining the $\mu _{\mathrm{VBF},\mathrm{V}\mathrm{H} }/\mu _\mathrm{{g} \mathrm{g} \mathrm{H} ,\mathrm{t}\mathrm{t}\mathrm{H} }$ results presented in Table 5 and Fig. 5 (right)

Parameter of interest: $R=\mu _{\mathrm{VBF},\mathrm{V}\mathrm{H} }/\mu _\mathrm{{g} \mathrm{g} \mathrm{H} ,\mathrm{t}\mathrm{t}\mathrm{H} }$					
Other parameters: $\mu _\mathrm{{g} \mathrm{g} \mathrm{H} ,\mathrm{t}\mathrm{t}\mathrm{H} }^{\gamma \gamma }$, $\mu _\mathrm{{g} \mathrm{g} \mathrm{H} ,\mathrm{t}\mathrm{t}\mathrm{H} }^\mathrm{{Z}\mathrm{Z}}$, $\mu _\mathrm{{g} \mathrm{g} \mathrm{H} ,\mathrm{t}\mathrm{t}\mathrm{H} }^{\mathrm{W}\mathrm{W}}$, $\mu _\mathrm{{g} \mathrm{g} \mathrm{H} ,\mathrm{t}\mathrm{t}\mathrm{H} }^{\tau \tau }$, and $\mu _\mathrm{{g} \mathrm{g} \mathrm{H} ,\mathrm{t}\mathrm{t}\mathrm{H} }^\mathrm{{b} \mathrm{b} }$					
Signal model	$\mathrm{H} \rightarrow \gamma \gamma$	$\mathrm{H} \rightarrow \mathrm{Z}\mathrm{Z}$	$\mathrm{H} \rightarrow \mathrm{W}\mathrm{W}$	$\mathrm{H} \rightarrow \tau \tau$	$\mathrm{H} \rightarrow \mathrm{b} \mathrm{b}$
$\mathrm{g} \mathrm{g} \mathrm{H}$	$\mu _\mathrm{{g} \mathrm{g} \mathrm{H} ,\mathrm{t}\mathrm{t}\mathrm{H} }^{\gamma \gamma }$	$\mu _\mathrm{{g} \mathrm{g} \mathrm{H} ,\mathrm{t}\mathrm{t}\mathrm{H} }^\mathrm{{Z}\mathrm{Z}}$	$\mu _\mathrm{{g} \mathrm{g} \mathrm{H} ,\mathrm{t}\mathrm{t}\mathrm{H} }^{\mathrm{W}\mathrm{W}}$	$\mu _\mathrm{{g} \mathrm{g} \mathrm{H} ,\mathrm{t}\mathrm{t}\mathrm{H} }^{\tau \tau }$	$\mu _\mathrm{{g} \mathrm{g} \mathrm{H} ,\mathrm{t}\mathrm{t}\mathrm{H} }^\mathrm{{b} \mathrm{b} }$
$\mathrm{VBF}$	$R\,\mu _\mathrm{{g} \mathrm{g} \mathrm{H} ,\mathrm{t}\mathrm{t}\mathrm{H} }^{\gamma \gamma }$	$R\,\mu _\mathrm{{g} \mathrm{g} \mathrm{H} ,\mathrm{t}\mathrm{t}\mathrm{H} }^\mathrm{{Z}\mathrm{Z}}$	$R\,\mu _\mathrm{{g} \mathrm{g} \mathrm{H} ,\mathrm{t}\mathrm{t}\mathrm{H} }^{\mathrm{W}\mathrm{W}}$	$R\,\mu _\mathrm{{g} \mathrm{g} \mathrm{H} ,\mathrm{t}\mathrm{t}\mathrm{H} }^{\tau \tau }$	$R\,\mu _\mathrm{{g} \mathrm{g} \mathrm{H} ,\mathrm{t}\mathrm{t}\mathrm{H} }^\mathrm{{b} \mathrm{b} }$
$\mathrm{V}\mathrm{H}$	$R\,\mu _\mathrm{{g} \mathrm{g} \mathrm{H} ,\mathrm{t}\mathrm{t}\mathrm{H} }^{\gamma \gamma }$	$R\,\mu _\mathrm{{g} \mathrm{g} \mathrm{H} ,\mathrm{t}\mathrm{t}\mathrm{H} }^\mathrm{{Z}\mathrm{Z}}$	$R\,\mu _\mathrm{{g} \mathrm{g} \mathrm{H} ,\mathrm{t}\mathrm{t}\mathrm{H} }^{\mathrm{W}\mathrm{W}}$	$R\,\mu _\mathrm{{g} \mathrm{g} \mathrm{H} ,\mathrm{t}\mathrm{t}\mathrm{H} }^{\tau \tau }$	$R\,\mu _\mathrm{{g} \mathrm{g} \mathrm{H} ,\mathrm{t}\mathrm{t}\mathrm{H} }^\mathrm{{b} \mathrm{b} }$
$\mathrm{t}\mathrm{t}\mathrm{H}$	$\mu _\mathrm{{g} \mathrm{g} \mathrm{H} ,\mathrm{t}\mathrm{t}\mathrm{H} }^{\gamma \gamma }$	$\mu _\mathrm{{g} \mathrm{g} \mathrm{H} ,\mathrm{t}\mathrm{t}\mathrm{H} }^\mathrm{{Z}\mathrm{Z}}$	$\mu _\mathrm{{g} \mathrm{g} \mathrm{H} ,\mathrm{t}\mathrm{t}\mathrm{H} }^{\mathrm{W}\mathrm{W}}$	$\mu _\mathrm{{g} \mathrm{g} \mathrm{H} ,\mathrm{t}\mathrm{t}\mathrm{H} }^{\tau \tau }$	$\mu _\mathrm{{g} \mathrm{g} \mathrm{H} ,\mathrm{t}\mathrm{t}\mathrm{H} }^\mathrm{{b} \mathrm{b} }$

Figure 5 (right) shows the likelihood scan of the data for $\mu _{\mathrm{VBF},\mathrm{V}\mathrm{H} }/\mu _\mathrm{{g} \mathrm{g} \mathrm{H} ,\mathrm{t}\mathrm{t}\mathrm{H} }$, while the bottom part of Table 5 shows the corresponding values; the best-fit $\mu _{\mathrm{VBF},\mathrm{V}\mathrm{H} }/\mu _\mathrm{{g} \mathrm{g} \mathrm{H} ,\mathrm{t}\mathrm{t}\mathrm{H} }$ is observed to be $1.25 ^{+0.62}_{-0.44}$, compatible with the expectation for the SM Higgs boson, $\mu _{\mathrm{VBF},\mathrm{V}\mathrm{H} }/\mu _\mathrm{{g} \mathrm{g} \mathrm{H} ,\mathrm{t}\mathrm{t}\mathrm{H} } =1$.

**Table 5:** The best-fit values for the signal strength of the $\mathrm{VBF}$ and $\mathrm{V}\mathrm{H}$ and of the $\mathrm{g} \mathrm{g} \mathrm{H}$ and $\mathrm{t}\mathrm{t}\mathrm{H}$ production mechanisms, $\mu _{\mathrm{VBF},\mathrm{V}\mathrm{H} }$ and $\mu _\mathrm{{g} \mathrm{g} \mathrm{H} ,\mathrm{t}\mathrm{t}\mathrm{H} }$, respectively, for $m_{\mathrm{{H}}} =125.0\,\text {GeV}$. The channels are grouped by decay mode tag as described in Sect. 3.3. The observed and median expected results for the ratio of $\mu _{\mathrm{VBF},\mathrm{V}\mathrm{H} }$ to $\mu _\mathrm{{g} \mathrm{g} \mathrm{H} ,\mathrm{t}\mathrm{t}\mathrm{H} }$ together with their uncertainties are also given for the full combination. In the full combination, $\mu _{\mathrm{VBF},\mathrm{V}\mathrm{H} }/\mu _\mathrm{{g} \mathrm{g} \mathrm{H} ,\mathrm{t}\mathrm{t}\mathrm{H} }$ is determined while profiling the five $\mu _\mathrm{{g} \mathrm{g} \mathrm{H} ,\mathrm{t}\mathrm{t}\mathrm{H} }$ parameters, one per decay mode, as shown in Table 4

Channel grouping	Best fit $(\mu _\mathrm{{g} \mathrm{g} \mathrm{H} ,\mathrm{t}\mathrm{t}\mathrm{H} }, \mu _{\mathrm{VBF},\mathrm{V}\mathrm{H} })$
$\mathrm{H} \rightarrow \gamma \gamma$ tagged	$(1.07, 1.24)$
$\mathrm{H} \rightarrow \mathrm{Z}\mathrm{Z}$ tagged	$(0.88, 1.75)$
$\mathrm{H} \rightarrow \mathrm{W}\mathrm{W}$ tagged	$(0.87, 0.66)$
$\mathrm{H} \rightarrow \tau \tau$ tagged	$(0.52, 1.21)$
$\mathrm{H} \rightarrow \mathrm{b} \mathrm{b}$ tagged	$(0.55, 0.85)$
Combined best fit $\mu _{\mathrm{VBF},\mathrm{V}\mathrm{H} }$/$\mu _\mathrm{{g} \mathrm{g} \mathrm{H} ,\mathrm{t}\mathrm{t}\mathrm{H} }$	
	Observed (expected)
	$1.25 ^{+0.62}_{-0.44}$ $(1.00 ^{+0.49}_{-0.35})$

### Individual production modes

While the production modes can be grouped by the type of interaction involved in the production of the SM Higgs boson, as done in Sect. 6.3, the data set and analyses available allow us to explore signal strength modifiers for different production modes, $\mu _\mathrm{{g} \mathrm{g} \mathrm{H} }$, $\mu _{\mathrm{VBF}}$, $\mu _{\mathrm{V}\mathrm{H} }$, and $\mu _\mathrm{{t}\mathrm{t}\mathrm{H} }$. These scaling factors are applied to the expected signal contributions from the SM Higgs boson according to their production mode, as shown in Table 6. It is assumed that the relative values of the branching fractions are those expected for the SM Higgs boson. This assumption is relaxed, in different ways, in Sects. 6.5 and 6.6.

**Table 6:** Parameterization used to scale the expected SM Higgs boson yields of the different production and decay modes when obtaining the results presented in Fig. 6

Parameters of interest: $\mu _\mathrm{{g} \mathrm{g} \mathrm{H} }$, $\mu _{\mathrm{VBF}}$, $\mu _{\mathrm{V}\mathrm{H} }$, and $\mu _\mathrm{{t}\mathrm{t}\mathrm{H} }$					
Signal model	$\mathrm{H} \rightarrow \gamma \gamma$	$\mathrm{H} \rightarrow \mathrm{Z}\mathrm{Z}$	$\mathrm{H} \rightarrow \mathrm{W}\mathrm{W}$	$\mathrm{H} \rightarrow \tau \tau$	$\mathrm{H} \rightarrow \mathrm{b} \mathrm{b}$
$\mathrm{g} \mathrm{g} \mathrm{H}$	$\mu _\mathrm{{g} \mathrm{g} \mathrm{H} }$	$\mu _\mathrm{{g} \mathrm{g} \mathrm{H} }$	$\mu _\mathrm{{g} \mathrm{g} \mathrm{H} }$	$\mu _\mathrm{{g} \mathrm{g} \mathrm{H} }$	$\mu _\mathrm{{g} \mathrm{g} \mathrm{H} }$
$\mathrm{VBF}$	$\mu _{\mathrm{VBF}}$	$\mu _{\mathrm{VBF}}$	$\mu _{\mathrm{VBF}}$	$\mu _{\mathrm{VBF}}$	$\mu _{\mathrm{VBF}}$
$\mathrm{V}\mathrm{H}$	$\mu _{\mathrm{V}\mathrm{H} }$	$\mu _{\mathrm{V}\mathrm{H} }$	$\mu _{\mathrm{V}\mathrm{H} }$	$\mu _{\mathrm{V}\mathrm{H} }$	$\mu _{\mathrm{V}\mathrm{H} }$
$\mathrm{t}\mathrm{t}\mathrm{H}$	$\mu _\mathrm{{t}\mathrm{t}\mathrm{H} }$	$\mu _\mathrm{{t}\mathrm{t}\mathrm{H} }$	$\mu _\mathrm{{t}\mathrm{t}\mathrm{H} }$	$\mu _\mathrm{{t}\mathrm{t}\mathrm{H} }$	$\mu _\mathrm{{t}\mathrm{t}\mathrm{H} }$

**Fig. 6:**
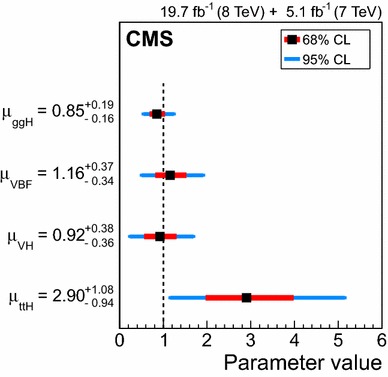
Likelihood scan results for $\mu _\mathrm{{g} \mathrm{g} \mathrm{H} }$, $\mu _{\mathrm{VBF}}$, $\mu _{\mathrm{V}\mathrm{H} }$, and $\mu _\mathrm{{t}\mathrm{t}\mathrm{H} }$. The *inner bars* represent the 68 % CL confidence intervals while the *outer bars* represent the 95 % CL confidence intervals. When scanning each individual parameter, the three other parameters are profiled. The SM values of the relative branching fractions are assumed for the different decay modes

Figure 6 summarizes the results of likelihood scans for the four parameters of interest described in Table 6 in terms of the 68 % CL (inner) and 95 % CL (outer) confidence intervals. When scanning the likelihood of the data as a function of one parameter, the other parameters are profiled.

Table 7 shows the best-fit results for the 7$\,\text {TeV}$ and 8$\,\text {TeV}$ data sets separately, as well as for the full combined analysis. Based on the combined likelihood ratio values for each parameter, Table 7 also shows the observed significance, the expected significance, and the pull of the results with respect to the SM hypothesis. The observed significance is derived from the observed likelihood ratio for the background-only hypothesis, $\mu _{i}=0$, in data. The expected significance is derived from the likelihood ratio for $\mu _{i}=0$ obtained using the median expected result for the SM Higgs boson. The pull with respect to the SM hypothesis is derived from the observed likelihood ratio for $\mu _{i}=1$; by definition, the expected pull with respect to the SM hypothesis is zero.

**Table 7:** The best-fit results for independent signal strengths scaling the $\mathrm{g} \mathrm{g} \mathrm{H}$, $\mathrm{VBF}$, $\mathrm{V}\mathrm{H}$, and $\mathrm{t}\mathrm{t}\mathrm{H}$ production processes; the expected and observed significances with respect to the background-only hypothesis, $\mu _{i}=0$; and the pull of the observation with respect to the SM hypothesis, $\mu _{i}=1$. The best-fit results are also provided separately for the 7$\,\text {TeV}$ and 8$\,\text {TeV}$ data sets, for which the predicted cross sections differ. These results assume that the relative values of the branching fractions are those predicted for the SM Higgs boson

Parameter	Best-fit result (68 % CL)			Significance ($\sigma$)		Pull to SM
	7$\,\text {TeV}$	8$\,\text {TeV}$	Combined	Observed	Expected	($\sigma$)
$\mu _\mathrm{{g} \mathrm{g} \mathrm{H} }$	$1.03^{+0.37}_{-0.33}$	$0.79^{+0.19}_{-0.17}$	$0.85 ^{+0.19}_{-0.16}$	$6.6$	$7.4$	$-0.8$
$\mu _{\mathrm{VBF}}$	$1.77^{+0.99}_{-0.90}$	$1.02^{+0.39}_{-0.36}$	$1.16 ^{+0.37}_{-0.34}$	$3.7$	$3.3$	$+0.4$
$\mu _{\mathrm{V}\mathrm{H} }$	${<}0.99$	$0.96^{+0.41}_{-0.39}$	$0.92 ^{+0.38}_{-0.36}$	$2.7$	$2.9$	$-0.2$
$\mu _\mathrm{{t}\mathrm{t}\mathrm{H} }$	${<}2.19$	$3.27^{+1.20}_{-1.04}$	$2.90 ^{+1.08}_{-0.94}$	$3.5$	$1.2$	$+2.2$

**Table 8:** Parameterization used to scale the expected SM Higgs boson yields of the different production and decay modes when obtaining the results presented in Table 9. The $\mu _\mathrm{{g} \mathrm{g} \mathrm{H} ,\mathrm{t}\mathrm{t}\mathrm{H} }$ and $\mu _{\mathrm{VBF},\mathrm{V}\mathrm{H} }$ parameters are introduced to reduce the dependency of the results on the SM expectation

Parameters of interest: $\lambda _{yy,xx}$, $\lambda _{ii,xx}$, $\lambda _{jj,xx}$, and $\lambda _{kk,xx}$					
Other parameters: $\mu _\mathrm{{g} \mathrm{g} \mathrm{H} ,\mathrm{t}\mathrm{t}\mathrm{H} }$ and $\mu _{\mathrm{VBF},\mathrm{V}\mathrm{H} }$					
Signal model	$\mathrm{H} \rightarrow xx$	$\mathrm{H} \rightarrow yy$	$\mathrm{H} \rightarrow ii$	$\mathrm{H} \rightarrow jj$	$\mathrm{H} \rightarrow kk$
$\mathrm{g} \mathrm{g} \mathrm{H}$	$\mu _\mathrm{{g} \mathrm{g} \mathrm{H} ,\mathrm{t}\mathrm{t}\mathrm{H} }$	$\mu _\mathrm{{g} \mathrm{g} \mathrm{H} ,\mathrm{t}\mathrm{t}\mathrm{H} } \,\lambda _{yy,xx}$	$\mu _\mathrm{{g} \mathrm{g} \mathrm{H} ,\mathrm{t}\mathrm{t}\mathrm{H} } \,\lambda _{ii,xx}$	$\mu _\mathrm{{g} \mathrm{g} \mathrm{H} ,\mathrm{t}\mathrm{t}\mathrm{H} } \,\lambda _{jj,xx}$	$\mu _\mathrm{{g} \mathrm{g} \mathrm{H} ,\mathrm{t}\mathrm{t}\mathrm{H} } \,\lambda _{kk,xx}$
$\mathrm{VBF}$	$\mu _{\mathrm{VBF},\mathrm{V}\mathrm{H} }$	$\mu _{\mathrm{VBF},\mathrm{V}\mathrm{H} } \,\lambda _{yy,xx}$	$\mu _{\mathrm{VBF},\mathrm{V}\mathrm{H} } \,\lambda _{ii,xx}$	$\mu _{\mathrm{VBF},\mathrm{V}\mathrm{H} } \,\lambda _{jj,xx}$	$\mu _{\mathrm{VBF},\mathrm{V}\mathrm{H} } \,\lambda _{kk,xx}$
$\mathrm{V}\mathrm{H}$	$\mu _{\mathrm{VBF},\mathrm{V}\mathrm{H} }$	$\mu _{\mathrm{VBF},\mathrm{V}\mathrm{H} } \,\lambda _{yy,xx}$	$\mu _{\mathrm{VBF},\mathrm{V}\mathrm{H} } \,\lambda _{ii,xx}$	$\mu _{\mathrm{VBF},\mathrm{V}\mathrm{H} } \,\lambda _{jj,xx}$	$\mu _{\mathrm{VBF},\mathrm{V}\mathrm{H} } \,\lambda _{kk,xx}$
$\mathrm{t}\mathrm{t}\mathrm{H}$	$\mu _\mathrm{{g} \mathrm{g} \mathrm{H} ,\mathrm{t}\mathrm{t}\mathrm{H} }$	$\mu _\mathrm{{g} \mathrm{g} \mathrm{H} ,\mathrm{t}\mathrm{t}\mathrm{H} } \,\lambda _{yy,xx}$	$\mu _\mathrm{{g} \mathrm{g} \mathrm{H} ,\mathrm{t}\mathrm{t}\mathrm{H} } \,\lambda _{ii,xx}$	$\mu _\mathrm{{g} \mathrm{g} \mathrm{H} ,\mathrm{t}\mathrm{t}\mathrm{H} } \,\lambda _{jj,xx}$	$\mu _\mathrm{{g} \mathrm{g} \mathrm{H} ,\mathrm{t}\mathrm{t}\mathrm{H} } \,\lambda _{kk,xx}$

The $\mu _\mathrm{{g} \mathrm{g} \mathrm{H} }$ best-fit value is found to be $0.85 ^{+0.19}_{-0.16}$. After calculating the component of the uncertainty that is statistical in nature (stat) and the component related to the theory inputs (theo), one can subtract them in quadrature from the total uncertainty and assign the remainder as the systematic uncertainty (syst), yielding $0.85\,^{+0.11}_{-0.09}\,\text {(stat)} \,^{+0.11}_{-0.08}\,\text {(theo)} \,^{+0.10}_{-0.09}\,\text {(syst)}$. Advances in the calculation of the $\mathrm{g} \mathrm{g} \mathrm{H}$ cross section, e.g. when considering higher-order effects, may not only reduce the uncertainty above, but also shift the central value. The signal strengths for the $\mathrm{VBF}$ and $\mathrm{V}\mathrm{H}$ production modes are assessed independently. Individual likelihood scans are performed as a function of $\mu _{\mathrm{VBF}}$ (or $\mu _{\mathrm{V}\mathrm{H} }$), allowing the modifiers associated with the other production processes to float in the fit together with the nuisance parameters. In data, the best-fit result for $\mu _{\mathrm{VBF}}$ is $1.16 ^{+0.37}_{-0.34}$, while for $\mu _{\mathrm{V}\mathrm{H} }$ it is $0.92 ^{+0.38}_{-0.36}$. For the $\mathrm{t}\mathrm{t}\mathrm{H}$ production mode, the best-fit value for $\mu _\mathrm{{t}\mathrm{t}\mathrm{H} }$ is found to be $2.90 ^{+1.08}_{-0.94}$. The results for $\mathrm{VBF}$, $\mathrm{V}\mathrm{H}$, and $\mathrm{t}\mathrm{t}\mathrm{H}$ are driven by the corresponding tagged categories, while the contribution from $\mathrm{g} \mathrm{g} \mathrm{H}$ is constrained by the 0-jet and untagged categories.

The results in Table 7 show a clear observation of Higgs bosons produced through gluon fusion, and evidence for the production of Higgs bosons through vector boson fusion, for which both the expected and observed significances are above the $3\sigma$ level. For $\mathrm{V}\mathrm{H}$ production, the expected significance is $2.9 \sigma$ and the observed significance is $2.7 \sigma$. The large best-fit value for $\mu _\mathrm{{t}\mathrm{t}\mathrm{H} }$ is compatible with the results presented and discussed in Sect. 6.2; the data are compatible with the $\mu _\mathrm{{t}\mathrm{t}\mathrm{H} } =1$ hypothesis at the $2.2\sigma$ level. Because of the different parameterizations used, this significance is not exactly the same as that found in Sect. 6.2 when considering the combination of $\mathrm{t}\mathrm{t}\mathrm{H}$-tagged categories.

**Table 9:** The best-fit results and 68 % CL confidence intervals for signal strength ratios of the decay mode in each column and the decay mode in each row, as modelled by the parameterization in Table 8. When the likelihood of the data is scanned as a function of each individual parameter, the three other parameters in the same row, as well the production cross sections modifiers $\mu _\mathrm{{g} \mathrm{g} \mathrm{H} ,\mathrm{t}\mathrm{t}\mathrm{H} }$ and $\mu _{\mathrm{VBF},\mathrm{V}\mathrm{H} }$, are profiled. Since each row corresponds to an independent fit to data, the relation $\lambda _{yy,xx}=1/\lambda _{xx,yy}$ is only approximately satisfied

Best-fit $\lambda _\text {col,row}$	$\mathrm{H} \rightarrow \gamma \gamma$	$\mathrm{H} \rightarrow \mathrm{Z}\mathrm{Z}$	$\mathrm{H} \rightarrow \mathrm{W}\mathrm{W}$	$\mathrm{H} \rightarrow \tau \tau$	$\mathrm{H} \rightarrow \mathrm{b} \mathrm{b}$
$\mathrm{H} \rightarrow \gamma \gamma$	1	$0.92^{+0.38}_{-0.27}$	$0.83^{+0.27}_{-0.22}$	$0.71^{+0.43}_{-0.25}$	$0.63^{+0.44}_{-0.35}$
$\mathrm{H} \rightarrow \mathrm{Z}\mathrm{Z}$	$1.06^{+0.44}_{-0.31}$	1	$0.88^{+0.38}_{-0.26}$	$0.76^{+0.43}_{-0.30}$	$0.65^{+0.59}_{-0.37}$
$\mathrm{H} \rightarrow \mathrm{W}\mathrm{W}$	$1.21^{+0.41}_{-0.31}$	$1.10^{+0.44}_{-0.33}$	1	$0.86^{+0.42}_{-0.32}$	$0.74^{+0.61}_{-0.41}$
$\mathrm{H} \rightarrow \tau \tau$	$1.41^{+0.75}_{-0.45}$	$1.31^{+0.81}_{-0.48}$	$1.15^{+0.68}_{-0.44}$	1	$0.87^{+0.69}_{-0.49}$
$\mathrm{H} \rightarrow \mathrm{b} \mathrm{b}$	$1.60^{+1.86}_{-0.70}$	$1.48^{+1.85}_{-0.70}$	$1.32^{+1.57}_{-0.59}$	$1.14^{+1.34}_{-0.52}$	1

**Table 10:** A completely general signal parameterization used to scale the expected yields of the $5\times 4$ different production and decay modes. The particular choice of parameters is such that the single-particle parameterization shown in Table 11 is a nested model, i.e. it can be obtained by assuming $\lambda _{i}^{j}=\lambda _{i}$, where $i$ runs through the production processes except $\mathrm{g} \mathrm{g} \mathrm{H}$ and $j$ runs through the decay modes. The expectation for the SM Higgs boson is $\lambda _{i}^{j}=\mu _{j}=1$. This parameterization is used in the denominator of the test statistic defined in Eq. (6)

All parameters constrained to be positive					
Signal model	$\mathrm{H} \rightarrow \gamma \gamma$	$\mathrm{H} \rightarrow \mathrm{Z}\mathrm{Z}$	$\mathrm{H} \rightarrow \mathrm{W}\mathrm{W}$	$\mathrm{H} \rightarrow \tau \tau$	$\mathrm{H} \rightarrow \mathrm{b} \mathrm{b}$
$\mathrm{g} \mathrm{g} \mathrm{H}$	$\mu _{\gamma \gamma }$	$\mu _\mathrm{{Z}\mathrm{Z}}$	$\mu _{\mathrm{W}\mathrm{W}}$	$\mu _{\tau \tau }$	$\mu _\mathrm{{b} \mathrm{b} }$
$\mathrm{VBF}$	$\lambda _{\mathrm{VBF}}^{\gamma \gamma } \,\mu _{\gamma \gamma }$	$\lambda _{\mathrm{VBF}}^\mathrm{{Z}\mathrm{Z}} \,\mu _\mathrm{{Z}\mathrm{Z}}$	$\lambda _{\mathrm{VBF}}^{\mathrm{W}\mathrm{W}} \,\mu _{\mathrm{W}\mathrm{W}}$	$\lambda _{\mathrm{VBF}}^{\tau \tau } \,\mu _{\tau \tau }$	$\lambda _{\mathrm{VBF}}^\mathrm{{b} \mathrm{b} } \,\mu _\mathrm{{b} \mathrm{b} }$
$\mathrm{V}\mathrm{H}$	$\lambda _{\mathrm{V}\mathrm{H} }^{\gamma \gamma } \,\mu _{\gamma \gamma }$	$\lambda _{\mathrm{V}\mathrm{H} }^\mathrm{{Z}\mathrm{Z}} \,\mu _\mathrm{{Z}\mathrm{Z}}$	$\lambda _{\mathrm{V}\mathrm{H} }^{\mathrm{W}\mathrm{W}} \,\mu _{\mathrm{W}\mathrm{W}}$	$\lambda _{\mathrm{V}\mathrm{H} }^{\tau \tau } \,\mu _{\tau \tau }$	$\lambda _{\mathrm{V}\mathrm{H} }^\mathrm{{b} \mathrm{b} } \,\mu _\mathrm{{b} \mathrm{b} }$
$\mathrm{t}\mathrm{t}\mathrm{H}$	$\lambda _\mathrm{{t}\mathrm{t}\mathrm{H} }^{\gamma \gamma } \,\mu _{\gamma \gamma }$	$\lambda _\mathrm{{t}\mathrm{t}\mathrm{H} }^\mathrm{{Z}\mathrm{Z}} \,\mu _\mathrm{{Z}\mathrm{Z}}$	$\lambda _\mathrm{{t}\mathrm{t}\mathrm{H} }^{\mathrm{W}\mathrm{W}} \,\mu _{\mathrm{W}\mathrm{W}}$	$\lambda _\mathrm{{t}\mathrm{t}\mathrm{H} }^{\tau \tau } \,\mu _{\tau \tau }$	$\lambda _\mathrm{{t}\mathrm{t}\mathrm{H} }^\mathrm{{b} \mathrm{b} } \,\mu _\mathrm{{b} \mathrm{b} }$

### Ratios between decay modes

Some of the largest uncertainties in SM Higgs predictions are related to the production cross sections. In an attempt to evade those uncertainties, it has been proposed [192, 193] to perform measurements of ratios of the signal strengths in different decay modes, $\lambda _{yy,xx} = \beta _{yy}/\beta _{xx}$, where $\beta _{xx}=\mathcal {B}(\mathrm{H} \rightarrow xx)/\mathcal {B}(\mathrm{H} \rightarrow xx)_\text {SM}$ and $\mathcal {B}$ denotes a branching fraction. In such $\beta _{xx}$ ratios, uncertainties related to the production and decay predictions for the Higgs boson, as well as some experimental uncertainties, may cancel out. On the other hand, the uncertainty in a given ratio will reflect the combined statistical uncertainties of both the $yy$ and $xx$ decay modes.

To probe the different $\lambda _{yy,xx}$, the expected signal yields for the different production and decay modes are scaled by the factors shown in Table 8. To reduce the dependency of the results on the expected structure of the SM Higgs production cross section, the $\mu _\mathrm{{g} \mathrm{g} \mathrm{H} ,\mathrm{t}\mathrm{t}\mathrm{H} }$ and $\mu _{\mathrm{VBF},\mathrm{V}\mathrm{H} }$ parameters are introduced and allowed to float independently. Therefore, these measurements only assume the SM ratio of $\mathrm{g} \mathrm{g} \mathrm{H}$ and $\mathrm{t}\mathrm{t}\mathrm{H}$ cross sections and the ratio of $\mathrm{VBF}$ and $\mathrm{V}\mathrm{H}$ cross sections.

Given the five decay modes that are currently accessible, four ratios can be probed at a time. For example, the choice of the $\mathrm{H} \rightarrow \gamma \gamma$ decay as denominator, $xx=\gamma \gamma$, fixes the four ratio parameters to be $\lambda _\mathrm{{Z}\mathrm{Z},\gamma \gamma }$, $\lambda _\mathrm{{b}\mathrm{b},\gamma \gamma }$, $\lambda _{\mathrm{W}\mathrm{W},\gamma \gamma }$, and $\lambda _{\tau \tau ,\gamma \gamma }$. When scanning the likelihood for the data as a function of a given $\lambda _{yy,xx}$ ratio, the production cross section modifiers $\mu _\mathrm{{g} \mathrm{g} \mathrm{H} ,\mathrm{t}\mathrm{t}\mathrm{H} }$ and $\mu _{\mathrm{VBF},\mathrm{V}\mathrm{H} }$, as well as the other three ratios, are profiled. The best-fit results for each choice of denominator are presented as the different rows in Table 9. While correlated uncertainties from theory and correlated experimental uncertainties may cancel out to some extent in these ratios, each ratio includes the statistical uncertainties from the two decay modes involved. For the available data set and analyses, the resulting statistical uncertainty dominates the total uncertainty. It can be seen that the SM expectation, $\lambda _{yy,xx}=1$, is inside the 68 % CL confidence interval for all measurements.

**Table 11:** A general single-state parameterization used to scale the expected yields of the different production and decay modes. For this parameterization the matrix has ${\text {rank}}(\mathcal {M}) =1$ by definition. It can be seen that this parameterization is nested in the general one presented in Table 10, and can be obtained by setting $\lambda _{i}^{j}=\lambda _{i}$, where $i$ runs through the production processes except $\mathrm{g} \mathrm{g} \mathrm{H}$ and $j$ runs through the decay modes. The expectation for the SM Higgs boson is $\lambda _{i}=\mu _{j}=1$. This parameterization is used in the numerator of the test statistic defined in Eq. (6)

All parameters constrained to be positive					
Signal model	$\mathrm{H} \rightarrow \gamma \gamma$	$\mathrm{H} \rightarrow \mathrm{Z}\mathrm{Z}$	$\mathrm{H} \rightarrow \mathrm{W}\mathrm{W}$	$\mathrm{H} \rightarrow \tau \tau$	$\mathrm{H} \rightarrow \mathrm{b} \mathrm{b}$
$\mathrm{g} \mathrm{g} \mathrm{H}$	$\mu _{\gamma \gamma }$	$\mu _\mathrm{{Z}\mathrm{Z}}$	$\mu _{\mathrm{W}\mathrm{W}}$	$\mu _{\tau \tau }$	$\mu _\mathrm{{b} \mathrm{b} }$
$\mathrm{VBF}$	$\lambda _{\mathrm{VBF}} \,\mu _{\gamma \gamma }$	$\lambda _{\mathrm{VBF}} \,\mu _\mathrm{{Z}\mathrm{Z}}$	$\lambda _{\mathrm{VBF}} \,\mu _{\mathrm{W}\mathrm{W}}$	$\lambda _{\mathrm{VBF}} \,\mu _{\tau \tau }$	$\lambda _{\mathrm{VBF}} \,\mu _\mathrm{{b} \mathrm{b} }$
$\mathrm{V}\mathrm{H}$	$\lambda _{\mathrm{V}\mathrm{H} } \,\mu _{\gamma \gamma }$	$\lambda _{\mathrm{V}\mathrm{H} } \,\mu _\mathrm{{Z}\mathrm{Z}}$	$\lambda _{\mathrm{V}\mathrm{H} } \,\mu _{\mathrm{W}\mathrm{W}}$	$\lambda _{\mathrm{V}\mathrm{H} } \,\mu _{\tau \tau }$	$\lambda _{\mathrm{V}\mathrm{H} } \,\mu _\mathrm{{b} \mathrm{b} }$
$\mathrm{t}\mathrm{t}\mathrm{H}$	$\lambda _\mathrm{{t}\mathrm{t}\mathrm{H} } \,\mu _{\gamma \gamma }$	$\lambda _\mathrm{{t}\mathrm{t}\mathrm{H} } \,\mu _\mathrm{{Z}\mathrm{Z}}$	$\lambda _\mathrm{{t}\mathrm{t}\mathrm{H} } \,\mu _{\mathrm{W}\mathrm{W}}$	$\lambda _\mathrm{{t}\mathrm{t}\mathrm{H} } \,\mu _{\tau \tau }$	$\lambda _\mathrm{{t}\mathrm{t}\mathrm{H} } \,\mu _\mathrm{{b} \mathrm{b} }$

### Search for mass-degenerate states with different coupling structures

One assumption that is made in Sect. 7 when studying the couplings of the Higgs boson is that the observations are due to the manifestation of a single particle. Alternatively, a superposition of states with indistinguishable mass values is expected in models or theories beyond the SM [194–197]. In this section we explore the validity of this assumption.

Taking advantage of the very good mass resolution in the $\mathrm{H} \rightarrow \gamma \gamma$ analysis, the presence of near mass-degenerate states has been previously probed down to mass differences between 2.5$\,\text {GeV}$ and 4$\,\text {GeV}$ without evidence for the presence of a second state [18]. Given the finite mass resolution, such searches are not sensitive to a mixture of states with mass values closer than the resolution itself, such that other reported measurements would integrate the contributions from both states.

In the case of two or more states with masses closer to each other than the experimental resolution, it becomes impossible to discern them using the mass observables. However, the distinction between states can still be made, provided that the states have different coupling structures, i.e. different coupling strengths to the SM particles. Using the measurements of the different production and decay tags, as well as the detailed knowledge of their expected composition in terms of production processes and decay modes, it is possible to test the compatibility of the observations with the expectations from a single state. Several authors discussed this possibility, proposing methods to look for deviations assuming that, in the presence of more than one state, the individual states would couple differently to the SM particles [198, 199].

A general parameterization of the $5\times 4$ matrix, $\mathcal {M}$, of signal strengths for the different production processes and decay modes is shown in Table 10. This parameterization has as many degrees of freedom as there are elements in the matrix and is completely general. Depending on whether there is one particle or more particles responsible for the observations in data, the algebraic properties of $\mathcal {M}$, namely its rank, ${\text {rank}}(\mathcal {M})$, will vary.

If there is only one state it follows that ${\text {rank}}(\mathcal {M}) = 1$, i.e. there should be one common multiplier per row and one common multiplier per column. A general matrix with ${\text {rank}}(\mathcal {M}) =1$ can be parameterized as shown in Table 11. This parameterization can also be obtained by taking the most general $5\times 4$ parameterization in Table 10 and assuming $\lambda _{i}^{j}=\lambda _{i}$, where $i$ runs through the production processes except $\mathrm{g} \mathrm{g} \mathrm{H}$ and $j$ runs through the decay modes. Given this relationship, the model for a general matrix with ${\text {rank}}(\mathcal {M}) =1$ presented in Table 11 is nested, in the statistics sense, in the general parameterization of the $5\times 4$ matrix presented in Table 10.

The expectation for the SM Higgs boson is a particular case of a rank 1 matrix, namely that for which $\lambda _{i}=\mu _{j}=1$, where $i$ runs through the production processes except $\mathrm{g} \mathrm{g} \mathrm{H}$ and $j$ runs through the decay modes.

If there is more than one particle contributing to the observations, the structure of $\mathcal {M}$ may be such that ${\text {rank}}(\mathcal {M}) > 1$ as a consequence of the different interaction strengths of the individual, yet mass-degenerate, states.

The procedure to test for the presence of mass-degenerate states proposed in Ref. [200] takes into account both the fact that there may be missing matrix elements and the fact that there are uncertainties in the measurements, including their correlations. A profile likelihood ratio test statistic, $q_\lambda$, is built using two different models for the structure of $\mathcal {M}$, namely those presented in Tables 10 and 11,

6 $$\begin{aligned} q_{\lambda }=-2 \ln \frac{ \mathcal {L}(\text {data}\,|\,\lambda ^{j}_{i}=\hat{\lambda }_{i},\hat{\mu _{j}}) }{\mathcal {L}(\text {data}\,|\,\hat{\lambda }^{j}_{i},\hat{\mu _{j}}^\prime )}. \end{aligned}$$

The test statistic $q_{\lambda }$ is a function of the 20 variables defined in Table 10: $\lambda _i^j$ and $\mu _j$, where the index $i$ runs through the $\mathrm{VBF}$, $\mathrm{V}\mathrm{H}$, and $\mathrm{t}\mathrm{t}\mathrm{H}$ production processes and the index $j$ runs through the decay modes. In this likelihood ratio, the model in Table 10 is taken as the alternative hypothesis and corresponds to the so-called “saturated model” in statistics, as it contains as many degrees of freedom as there are elements in $\mathcal {M}$. The null hypothesis model is the one presented in Table 11, which parameterizes $\mathcal {M}$ as a general rank 1 matrix, where all rows are multiples of each other, as expected for a single particle. If the observations are due to a single particle, the $\lambda _i$ do not depend on the decay mode and the value of the $q_{\lambda }$ is not very large, since both hypotheses fit the data equally well. However, for a matrix with ${\text {rank}}(\mathcal {M}) \ne 1$, the most general $5\times 4$ matrix model will fit the data better than the general rank 1 matrix model and the value of $q_{\lambda }$ is expected to be large.

**Fig. 7:**
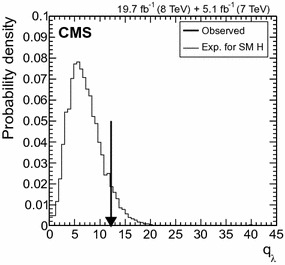
Distribution of the profile likelihood ratio $q_\lambda$ between different assumptions for the structure of the matrix of signal strengths for the production processes and decay modes both for pseudo-data samples generated under the SM hypothesis and the value observed in data. The likelihood in the numerator is that for the data under a model of a general rank 1 matrix, expected if the observations are due to a single particle and of which the SM is a particular case. The likelihood in the denominator is that for the data under a “saturated model” with as many parameters as there are matrix elements. The *arrow* represents the observed value in data, $q_\lambda ^\text {obs}$. Under the SM hypothesis, the probability to find a value of $q_\lambda \ge q_\lambda ^\text {obs}$ is $(7.9\pm 0.3)\,\%$, where the uncertainty reflects only the finite number of pseudo-data samples generated

The compatibility of the value of the test statistic observed in data, $q_\lambda ^\text {obs}$, with the expectation from the SM is evaluated using pseudo-data samples randomly generated under the SM hypothesis. Figure 7 shows the distribution of $q_\lambda$ for the SM pseudo-data samples as well as the value observed in data, $q_\lambda ^\text {obs}=12.2$. Under the SM hypothesis, we find that the probability of observing a value of $q_\lambda \ge q_\lambda ^\text {obs}$ is $(7.9\pm 0.3)\,\%$, where the uncertainty reflects only the finite number of pseudo-data samples generated. Such a $p\text {-value}$ corresponds to a deviation from the SM expectation of about $1.4\sigma$. This small tension, not present in previous tests performed in this section, is due to the observed data in the dijet-tagged channel of the $\mathrm{H} \rightarrow \mathrm{Z}\mathrm{Z}$ analysis; performing a fit to a model where the $\mathrm{VBF}$ and $\mathrm{V}\mathrm{H}$ production modes are floated separately shows that the data prefer a very large $\mathrm{V}\mathrm{H}$ contribution and a very small $\mathrm{VBF}$ contribution. When $\mathrm{H} \rightarrow \mathrm{Z}\mathrm{Z}$ analysis inputs are not considered, the $p\text {-value}$ is found to be about 33 %.

## Compatibility of the observed data with the SM Higgs boson couplings

Whereas in Sect. 6 the production and decay of the boson were explored separately, the studies presented in this section simultaneously investigate the couplings of the boson to SM particles in the production and decay processes. In this way, correlations are handled consistently between the production modes and the decay modes. For example, the coupling of the SM Higgs boson to the $\mathrm{Z}$ boson is involved both in the $\mathrm{Z}\mathrm{H}$ production mode and the $\mathrm{H} \rightarrow \mathrm{Z}\mathrm{Z}$ decay mode, such that more information can be extracted from a simultaneous modelling of the production and decay modes in terms of the couplings involved.

Following the framework laid out in Ref. [171], we assume the signal arises from a single particle with $J^{PC} =0^{++}$ and a width such that the narrow-width approximation holds, permitting its production and decay to be considered independently. These assumptions are supported by the results of Sect. 6.6 on the presence of more particles at the same mass, those of Refs. [40, 41] regarding alternative $J^{P}$ assignments and mixtures, and those of Ref. [27] concerning the width of the particle.

Under the assumptions above, the event yield in a given (production)$\times$(decay) mode is related to the production cross section and the partial and total Higgs boson decay widths via

7 $$\begin{aligned} \left( \sigma \;\mathcal {B}\right) ( x \rightarrow \mathrm{H} \rightarrow yy) = \frac{\sigma _{ x}\;\varGamma _{ yy}}{\varGamma _{\text {tot}}}, \end{aligned}$$

where $\sigma _{ x }$ is the production cross section through process $x$, which includes $\mathrm{g} \mathrm{g} \mathrm{H}$, $\mathrm{VBF}$, $\mathrm{W}\mathrm{H}$, $\mathrm{Z}\mathrm{H}$, and $\mathrm{t}\mathrm{t}\mathrm{H}$; $\varGamma _{ yy }$ is the partial decay width into the final state $yy$, such as $\mathrm{W}\mathrm{W}$, $\mathrm{Z}\mathrm{Z}$, $\mathrm{b} \mathrm{b}$, $\tau \tau$, $\mathrm{g} \mathrm{g}$, or $\gamma \gamma$; and $\varGamma _{\text {tot}}$ is the total width of the boson.

Some quantities, such as $\sigma _\mathrm{{g} \mathrm{g} \mathrm{H} }$, $\varGamma _\mathrm{{g} \mathrm{g} }$, and $\varGamma _{\gamma \gamma }$, are generated by loop diagrams and, therefore, are sensitive to the presence of certain particles beyond the standard model (BSM). The possibility of Higgs boson decays to BSM particles, with a partial width $\varGamma _{\mathrm{BSM}}$, can also be accommodated by considering $\varGamma _{\text {tot}}$ as a dependent parameter so that $\varGamma _{\text {tot}} = \sum \varGamma _{ yy } + \varGamma _{\mathrm{BSM}}$, where $\sum \varGamma _{ yy }$ stands for the sum over partial widths for all decays to SM particles. With the data from the $\mathrm{H} ({\mathrm{inv}})$ searches, $\varGamma _{\mathrm{BSM}}$ can be further broken down as $\varGamma _{\mathrm{BSM}} = \varGamma _{\mathrm{inv}} + \varGamma _{\mathrm{undet}}$, where $\varGamma _{\mathrm{inv}}$ can be constrained by searches for invisible decays of the Higgs boson and $\varGamma _{\mathrm{undet}}$ corresponds to Higgs boson decays not fitting into the previous definitions. The definition of $\varGamma _{\mathrm{undet}}$ is such that two classes of decays can give rise to $\varGamma _{\mathrm{undet}}>0$: (i) BSM decays not studied in the analyses used in this paper, such as hypothetical lepton flavour violating decays, e.g. $\mathrm{H} \rightarrow \mathrm{\mu }\mathrm{\tau }$, and (ii) decays that might not be detectable with the existing experimental setup because of the trigger conditions of the experiment, such as hypothetical decays resulting in a large multiplicity of low-$p_{\mathrm{T}}$ particles.

To test the observed data for possible deviations from the rates expected for the SM Higgs boson in the different channels, we introduce coupling modifiers, denoted by the scale factors $\kappa _{i}$ [171]. The scale factors are defined for production processes by $\kappa _{i}^2=\sigma _{i}/\sigma _{i}^\text {SM}$, for decay processes by $\kappa _{i}^2=\varGamma _{ii}/\varGamma _{ii}^\text {SM}$, and for the total width by $\kappa _\mathrm{{H}}^2=\varGamma _\text {tot}/\varGamma _{\mathrm{SM}}$, where the SM values are tabulated in Ref. [171]. When considering the different $\kappa _{i}$, the index $i$ can represent many ways to test for deviations:

- For SM particles with tree-level couplings to the Higgs boson: $\kappa _{{\mathrm{W}}}$ ($\mathrm{W}$ bosons), $\kappa _{\mathrm{{Z}}}$ ($\mathrm{Z}$ bosons), $\kappa _\mathrm{{b}}$ (bottom quarks), $\kappa _{\tau }$ (tau leptons), $\kappa _\mathrm{{t}}$ (top quarks), and $\kappa _{\mathrm{\mu }}$ (muons). Unless otherwise noted, the scaling factors for other fermions are tied to those that can be constrained by data.
- Particular symmetries of the SM make it interesting to test for deviations in whole classes of particles, leading to $\kappa _{\mathrm{V}}$ (massive vector bosons), $\kappa _{\mathrm{f}}$ (fermions), $\kappa _{\ell }$ (leptons), $\kappa _\mathrm{{q}}$ (quarks), $\kappa _\mathrm{{u}}$ (up-type fermions), and $\kappa _\mathrm{{d}}$ (down-type fermions).
- For SM particles with loop-induced couplings, the scaling factors can be expressed in terms of the tree-level coupling modifiers, assuming the SM loop structure, but can also be taken as effective coupling modifiers: $\kappa _{\mathrm{{g}}}$ (gluons) and $\kappa _{\gamma }$ (photons).
- The scaling factors for couplings to second generation fermions are equal to those for the third generation: $\kappa _\mathrm{{s}} =\kappa _\mathrm{{b}}$, $\kappa _{\mathrm{\mu }} =\kappa _{\tau }$, and $\kappa _\mathrm{{c}} =\kappa _\mathrm{{t}}$, except in Sect. 7.4, where $\kappa _{\mathrm{\mu }}$ is constrained from the analysis of $\mathrm{H} \rightarrow \mu \mu$ decays.

Given their small expected contributions, the couplings to electrons, up quarks, and down quarks, are neglected.

In addition to the $\kappa _{i}$ parameters, the existence of BSM decays, invisible decays, and undetectable decays of the Higgs boson is considered; the corresponding branching fractions are denoted $\mathrm{BR}_\mathrm{BSM}$, $\mathrm{BR}_\mathrm{inv}$, and $\mathrm{BR}_\text {undet}$, as in Ref. [171].

Significant deviations of any $\kappa$ parameter from unity or of any ${\mathrm{BR}}$ parameter from zero would imply new physics beyond the SM Higgs boson hypothesis. The size of the current data set is insufficient to precisely quantify all phenomenological parameters defining the Higgs boson production and decay rates. Therefore, we present a set of combined analyses of different numbers of parameters, where the remaining parameters are either set to the SM expectations or profiled in the likelihood scans together with all other nuisance parameters. The value of $m_\mathrm{{H}}$ is fixed to the measured value of $125.0\,\text {GeV}$, as determined in Sect. 4.1. Since results for the individual channels are based on different assumed values of the mass, differences should be expected when comparing the previously published results from the individual channels with those in this combined analysis.

This section is organized as follows. In Sect. 7.1 we explore whether $\kappa _{{\mathrm{W}}}$ and $\kappa _{\mathrm{{Z}}}$ are compatible with each other and can be meaningfully used together as $\kappa _{\mathrm{V}}$. In Sect. 7.2 we test for deviations that would affect the couplings of massive vector bosons and fermions differently. The scaling factors among different types of fermions, leptons versus quarks and up-type versus down-type, are investigated in Sect. 7.3. In Sect. 7.4, we consider the results of a fit for the tree-level coupling scaling factors and the relation between the observations and the corresponding particle masses. We then turn to the study of models where BSM physics could manifest itself in loops ($\kappa _{\mathrm{{g}}}$, $\kappa _{\gamma }$) or decays ($\mathrm{BR}_\mathrm{BSM}$, $\mathrm{BR}_\mathrm{inv}$, $\mathrm{BR}_\text {undet}$). In Sect. 7.5 the tree-level couplings are constrained to those expected in the SM, and the searches for $\mathrm{H} ({\mathrm{inv}})$ are included. This restriction is lifted in Sect. 7.6, where a coupling scaling factor for the massive vector bosons and individual fermion coupling scaling factors are allowed to float, while in Sect. 7.7 the total width scaling factor is also left free to float. In Sect. 7.8, the results from the searches for invisible decays are included, and from the combination of the visible and invisible decays, limits on $\mathrm{BR}_\text {undet}$ are set. Table 12 summarizes the results of the tests performed.

**Table 12:** Tests of the compatibility of the data with the SM Higgs boson couplings. The best-fit values and 68 % and 95 % CL confidence intervals are given for the evaluated scaling factors $\kappa _{i}$ or ratios $\lambda _{ij}=\kappa _{i}/\kappa _{j}$. The different compatibility tests discussed in the text are separated by horizontal lines. When one of the parameters in a group is evaluated, others are treated as nuisance parameters

Model parameters	Table in Ref. [171]	Parameter	Best-fit result		Comment
			68 % CL	95 % CL	
$\kappa _{\mathrm{{Z}}}$, $\lambda _{{\mathrm{W}\mathrm{Z}}}$ ($\kappa _{\mathrm{f}}$ = 1)	–	$\lambda _{{\mathrm{W}\mathrm{Z}}}$	$0.94 ^{+0.22}_{-0.18}$	$[0.61,1.45]$	$\lambda _{{\mathrm{W}\mathrm{Z}}} =\kappa _{{\mathrm{W}}}/\kappa _{\mathrm{{Z}}}$ from $\mathrm{Z}\mathrm{Z}$ and 0/1-jet $\mathrm{W}\mathrm{W}$ channels
$\kappa _{\mathrm{{Z}}}$, $\lambda _{{\mathrm{W}\mathrm{Z}}}$, $\kappa _{\mathrm{f}}$	44 (top)	$\lambda _{{\mathrm{W}\mathrm{Z}}}$	$0.92 ^{+0.14}_{-0.12}$	$[0.71,1.24]$	$\lambda _{{\mathrm{W}\mathrm{Z}}} =\kappa _{{\mathrm{W}}}/\kappa _{\mathrm{{Z}}}$ from full combination
$\kappa _{\mathrm{V}}$, $\kappa _{\mathrm{f}}$	43 (top)	$\kappa _{\mathrm{V}}$	$1.01 ^{+0.07}_{-0.07}$	$[0.87,1.14]$	$\kappa _{\mathrm{V}}$ scales couplings to $\mathrm{W}$ and $\mathrm{Z}$ bosons
		$\kappa _{\mathrm{f}}$	$0.87 ^{+0.14}_{-0.13}$	$[0.63,1.15]$	$\kappa _{\mathrm{f}}$ scales couplings to all fermions
$\kappa _{\mathrm{V}}$, $\lambda _{\mathrm{{d} \mathrm{u}}}$, $\kappa _\mathrm{{u}}$	46 (top)	$\lambda _{\mathrm{{d} \mathrm{u}}}$	$0.99 ^{+0.19}_{-0.18}$	$[0.65,1.39]$	$\lambda _{\mathrm{{d} \mathrm{u}}} =\kappa _\mathrm{{u}}/\kappa _\mathrm{{d}}$, relates up-type and down-type fermions
$\kappa _{\mathrm{V}}$, $\lambda _{\mathrm{\ell \mathrm{q}}}$, $\kappa _\mathrm{{q}}$	47 (top)	$\lambda _{\mathrm{\ell \mathrm{q}}}$	$1.03 ^{+0.23}_{-0.21}$	$[0.62,1.50]$	$\lambda _{\mathrm{\ell \mathrm{q}}} =\kappa _{\ell }/\kappa _\mathrm{{q}}$, relates leptons and quarks
$\kappa _{{\mathrm{W}}}$, $\kappa _{\mathrm{{Z}}}$, $\kappa _\mathrm{{t}}$, $\kappa _\mathrm{{b}}$, $\kappa _{\tau }$, $\kappa _{\mathrm{\mu }}$	Extends 51	$\kappa _{{\mathrm{W}}}$	$0.95~^{+0.14}_{-0.13}$	$[0.68,1.23]$	
		$\kappa _{\mathrm{{Z}}}$	$1.05~^{+0.16}_{-0.16}$	$[0.72,1.35]$	
		$\kappa _\mathrm{{t}}$	$0.81~^{+0.19}_{-0.15}$	$[0.53,1.20]$	Up-type quarks (via $\mathrm{t}$)
		$\kappa _\mathrm{{b}}$	$0.74~^{+0.33}_{-0.29}$	$[0.09,1.44]$	Down-type quarks (via $\mathrm{b}$)
		$\kappa _{\tau }$	$0.84~^{+0.19}_{-0.18}$	$[0.50,1.24]$	$\kappa _{\tau }$ scales the coupling to tau leptons
		$\kappa _{\mathrm{\mu }}$	$0.49~^{+1.38}_{-0.49}$	$[0.00,2.77]$	$\kappa _{\mathrm{\mu }}$ scales the coupling to muons
$M$, $\epsilon$	Ref. [206]	$M$ ($\,\text {GeV}$)	$245 \pm 15$	$[217,279]$	$\kappa _{\mathrm{f}} = v \frac{m_{\mathrm{f}}^{\epsilon }}{M^{1+\epsilon }}$ and $\kappa _{\mathrm{V}} = v \frac{m_{\mathrm{V}}^{2\epsilon }}{M^{1+2\epsilon }{}_{}}$ (Sect. 7.4)
		$\epsilon$	$0.014 ^{+0.041}_{-0.036}$	$[-0.054,0.100]$	
$\kappa _{\mathrm{{g}}}$, $\kappa _{\gamma }$	48 (top)	$\kappa _{\mathrm{{g}}}$	$0.89 ^{+0.11}_{-0.10}$	$[0.69,1.11]$	Effective couplings to gluons ($\mathrm{g}$) and photons ($\gamma$)
		$\kappa _{\gamma }$	$1.14 ^{+0.12}_{-0.13}$	$[0.89,1.40]$	
$\kappa _{\mathrm{{g}}}$, $\kappa _{\gamma }$, $\mathrm{BR}_\mathrm{BSM}$	48 (middle)	$\mathrm{BR}_\mathrm{BSM}$	$\le 0.14$	$[0.00,0.32]$	Allows for BSM decays
With $\mathrm{H} ({\mathrm{inv}})$ searches	–	$\mathrm{BR}_\mathrm{inv}$	$0.03 ~^{+0.15}_{-0.03}$	$[0.00,0.32]$	$\mathrm{H} ({\mathrm{inv}})$ use implies $\mathrm{BR}_\text {undet}$ =0
With $\mathrm{H} ({\mathrm{inv}})$ and $\kappa _i=1$	–	$\mathrm{BR}_\mathrm{inv}$	$0.06 ~^{+0.11}_{-0.06}$	$[0.00,0.27]$	Assumes $\kappa _i=1$ and uses $\mathrm{H} ({\mathrm{inv}})$
$\kappa _\mathrm{{g} \mathrm{Z}}$, $\lambda _{{\mathrm{W}\mathrm{Z}}}$, $\lambda _\mathrm{{Z}\mathrm{g}}$, $\lambda _\mathrm{{b}\mathrm{Z}}$, $\lambda _{\gamma \mathrm{Z}}$, $\lambda _{\tau \mathrm{Z}}$, $\lambda _\mathrm{{t}\mathrm{g}}$	50 (bottom)	$\kappa _\mathrm{{g} \mathrm{Z}}$	$0.98~^{+0.14}_{-0.13}$	$[0.73,1.27]$	$\kappa _\mathrm{{g} \mathrm{Z}} =\kappa _{\mathrm{{g}}} \kappa _{\mathrm{{Z}}}/\kappa _\mathrm{{H}}$, i.e. floating $\kappa _\mathrm{{H}}$
		$\lambda _{{\mathrm{W}\mathrm{Z}}}$	$0.87~^{+0.15}_{-0.13}$	$[0.63,1.19]$	$\lambda _{{\mathrm{W}\mathrm{Z}}} =\kappa _{{\mathrm{W}}}/\kappa _{\mathrm{{Z}}}$
		$\lambda _\mathrm{{Z}\mathrm{g}}$	$1.39~^{+0.36}_{-0.28}$	$[0.87,2.18]$	$\lambda _\mathrm{{Z}\mathrm{g}} =\kappa _{\mathrm{{Z}}}/\kappa _{\mathrm{{g}}}$
		$\lambda _\mathrm{{b}\mathrm{Z}}$	$0.59~^{+0.22}_{-0.23}$	${\le }1.07$	$\lambda _\mathrm{{b}\mathrm{Z}} =\kappa _\mathrm{{b}}/\kappa _{\mathrm{{Z}}}$
		$\lambda _{\gamma \mathrm{Z}}$	$0.93~^{+0.17}_{-0.14}$	$[0.67,1.31]$	$\lambda _{\gamma \mathrm{Z}} =\kappa _{\gamma }/\kappa _{\mathrm{{Z}}}$
		$\lambda _{\tau \mathrm{Z}}$	$0.79~^{+0.19}_{-0.17}$	$[0.47,1.20]$	$\lambda _{\tau \mathrm{Z}} =\kappa _{\tau }/\kappa _{\mathrm{{Z}}}$
		$\lambda _\mathrm{{t}\mathrm{g}}$	$2.18~^{+0.54}_{-0.46}$	$[1.30,3.35]$	$\lambda _\mathrm{{t}\mathrm{g}} =\kappa _\mathrm{{t}}/\kappa _{\mathrm{{g}}}$
$\kappa _{\mathrm{V}}$, $\kappa _\mathrm{{b}}$, $\kappa _{\tau }$, $\kappa _\mathrm{{t}}$, $\kappa _{\mathrm{{g}}}$, $\kappa _{\gamma }$	Similar to 50 (top)	$\kappa _{\mathrm{V}}$	$0.96 ^{+0.14}_{-0.15}$	$[0.66,1.23]$	
		$\kappa _\mathrm{{b}}$	$0.64 ^{+0.28}_{-0.29}$	$[0.00,1.23]$	Down-type quarks (via $\mathrm{b}$)
		$\kappa _{\tau }$	$0.82 ^{+0.18}_{-0.18}$	$[0.48,1.20]$	Charged leptons (via $\tau$)
		$\kappa _\mathrm{{t}}$	$1.60 ^{+0.34}_{-0.32}$	$[0.97,2.28]$	Up-type quarks (via $\mathrm{t}$)
		$\kappa _{\mathrm{{g}}}$	$0.75 ^{+0.15}_{-0.13}$	$[0.52,1.07]$	
		$\kappa _{\gamma }$	$0.98 ^{+0.17}_{-0.16}$	$[0.67,1.33]$	
With $\kappa _{\mathrm{V}} \le 1$ and $\mathrm{BR}_\mathrm{BSM}$	–	$\mathrm{BR}_\mathrm{BSM}$	${\le }0.34$	$[0.00,0.57]$	Allows for BSM decays
With $\kappa _{\mathrm{V}} \le 1$ and $\mathrm{H} ({\mathrm{inv}})$	–	$\mathrm{BR}_\mathrm{inv}$	$0.17\pm 0.17$	$[0.00,0.49]$	$\mathrm{H} ({\mathrm{inv}})$ use implies $\mathrm{BR}_\text {undet} =0$
With $\kappa _{\mathrm{V}} \le 1$, $\mathrm{H} ({\mathrm{inv}})$, $\mathrm{BR}_\mathrm{inv}$, and $\mathrm{BR}_\text {undet}$	–	$\mathrm{BR}_\mathrm{inv}$	$0.17\pm 0.17$	$[0.00,0.49]$	Separates $\mathrm{BR}_\mathrm{inv}$ from $\mathrm{BR}_\text {undet}$,$\mathrm{BR}_\mathrm{BSM} =\mathrm{BR}_\mathrm{inv} +\mathrm{BR}_\text {undet}$
	–	$\mathrm{BR}_\text {undet}$	${\le }0.23$	$[0.00,0.52]$	

### Relation between the coupling to the $\mathrm{W}$ and $\mathrm{Z}$ bosons

In the SM, the Higgs sector possesses an approximate ${\mathrm{SU(2)_L \times SU(2)_R}}$ global symmetry, which is broken by the Higgs vacuum expectation value to the diagonal subgroup ${\mathrm{SU(2)_{L+R}}}$. As a result, the tree-level ratios of the $\mathrm{W}$ and $\mathrm{Z}$ boson masses, $m_{\mathrm{W}} / m_\mathrm{{Z}}$, and the ratio of their couplings to the Higgs boson, $g_{\mathrm{W}} / g_\mathrm{{Z}}$, are protected against large radiative corrections, a property known as “custodial symmetry” [201, 202]. However, large violations of custodial symmetry are possible in new physics models. We focus on the two scaling factors $\kappa _{{\mathrm{W}}}$ and $\kappa _{\mathrm{{Z}}}$ that modify the couplings of the SM Higgs boson to the $\mathrm{W}$ and $\mathrm{Z}$ bosons and perform two different combined analyses to assess the consistency of the ratio $\lambda _{{\mathrm{W}\mathrm{Z}}} = \kappa _{{\mathrm{W}}}/ \kappa _{\mathrm{{Z}}}$ with unity.

**Fig. 8:**
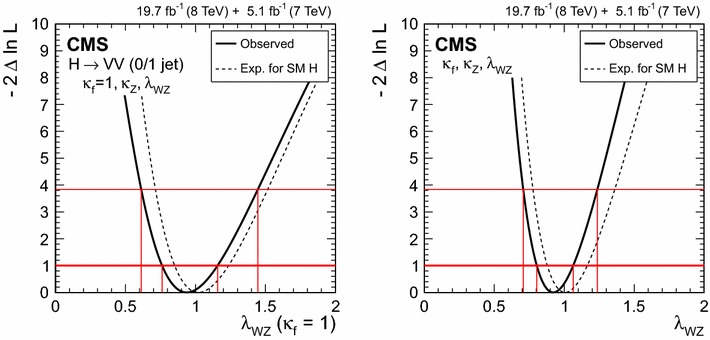
Likelihood scans versus $\lambda _{{\mathrm{W}\mathrm{Z}}}$, the ratio of the coupling scaling factors to $\mathrm{W}$ and $\mathrm{Z}$ bosons: (*left*) from untagged $\text {pp} \rightarrow \mathrm{H} \rightarrow \mathrm{W}\mathrm{W}$ and $\text {pp} \rightarrow \mathrm{H} \rightarrow \mathrm{Z}\mathrm{Z}$ searches, assuming the SM couplings to fermions, $\kappa _{\mathrm{f}} =1$; (*right*) from the combination of all channels, profiling the coupling to fermions. The *solid curve* represents the observation in data. The *dashed curve* indicates the expected median result in the presence of the SM Higgs boson. Crossings with the *horizontal thick* and *thin lines* denote the 68 % CL and 95 % CL confidence intervals, respectively

The dominant production mechanism populating the 0-jet and 1-jet channels of the $\mathrm{H} \rightarrow \mathrm{W}\mathrm{W} \rightarrow \ell \nu \ell \nu$ analysis and the untagged channels of the $\mathrm{H} \rightarrow \mathrm{Z}\mathrm{Z} \rightarrow 4\ell$ analysis is $\mathrm{g} \mathrm{g} \mathrm{H}$. Therefore, the ratio of event yields in these channels provides a nearly model-independent measurement of $\lambda _{{\mathrm{W}\mathrm{Z}}}$. We perform a combined analysis of these two channels with two free parameters, $\kappa _{\mathrm{{Z}}}$ and $\lambda _{{\mathrm{W}\mathrm{Z}}}$. The likelihood scan versus $\lambda _{{\mathrm{W}\mathrm{Z}}}$ is shown in Fig. 8 (left). The scale factor $\kappa _{\mathrm{{Z}}}$ is treated as a nuisance parameter. The result is $\lambda _{{\mathrm{W}\mathrm{Z}}} =0.94 ^{+0.22}_{-0.18}$, i.e. the data are consistent with the SM expectation ($\lambda _{{\mathrm{W}\mathrm{Z}}} =1$).

**Fig. 9:**
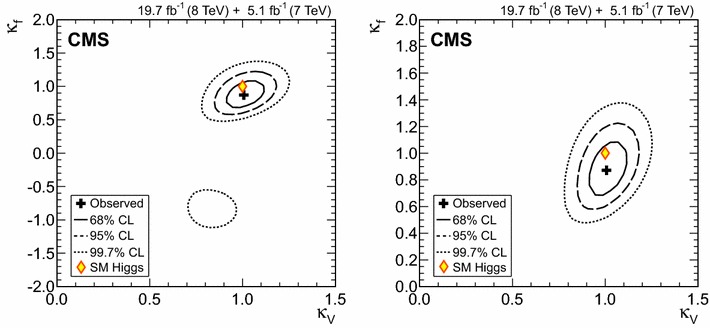
Results of 2D likelihood scans for the $\kappa _{\mathrm{V}}$ and $\kappa _{\mathrm{f}}$ parameters. The *cross* indicates the best-fit values. The *solid*, *dashed*, and *dotted contours* show the 68 %, 95 %, and 99.7 % CL confidence regions, respectively. The *diamond* shows the SM point $(\kappa _{\mathrm{V}}, \kappa _{\mathrm{f}})=(1,1)$. The *left plot* shows the likelihood scan in two quadrants, $(+,+)$ and $(+,-)$. The *right plot* shows the likelihood scan constrained to the $(+,+)$ quadrant

We also extract $\lambda _{{\mathrm{W}\mathrm{Z}}}$ from the combined analysis of all channels. In this approach, we introduce three parameters: $\lambda _{{\mathrm{W}\mathrm{Z}}}$, $\kappa _{\mathrm{{Z}}}$, and $\kappa _{\mathrm{f}}$. The BSM Higgs boson width $\varGamma _{\mathrm{BSM}}$ is set to zero. The partial width $\varGamma _\text {gg}$, induced by top and bottom quark loops, scales as $\kappa _{\mathrm{f}} ^2$. The partial width $\varGamma _{\gamma \gamma }$ is induced via loop diagrams, with the $\mathrm{W}$ boson and top quark being the dominant contributors, and is scaled with $\kappa _{\gamma } ^2(\kappa _\mathrm{{b}},\kappa _{\tau },\kappa _\mathrm{{t}},\kappa _{{\mathrm{W}}})$, a function defined in Eq. (113) of Ref. [171]. In the likelihood scan as a function of $\lambda _{{\mathrm{W}\mathrm{Z}}}$, both $\kappa _{\mathrm{{Z}}}$ and $\kappa _{\mathrm{f}}$ are profiled together with all other nuisance parameters. The introduction of $\kappa _{\mathrm{f}}$ carries with it the assumption that the coupling to all fermions is common, but possibly different from the SM expectation. The likelihood scan is shown in Fig. 8 (right) with a solid curve. The dashed curve indicates the median expected result for the SM Higgs boson, given the current data set. The measured value from the combined analysis of all channels is $\lambda _{{\mathrm{W}\mathrm{Z}}} = 0.92 ^{+0.14}_{-0.12}$ and is consistent with the expectation from the SM.

Given these results, and unless otherwise noted, in all subsequent measurements we assume $\lambda _{{\mathrm{W}\mathrm{Z}}} =1$ and use a common factor $\kappa _{\mathrm{V}}$ to modify the couplings to $\mathrm{W}$ and $\mathrm{Z}$ bosons, while preserving their ratio.

### Test of the couplings to massive vector bosons and fermions

In the SM, the nature of the coupling of the Higgs boson to fermions, through a Yukawa interaction, is different from the nature of the Higgs boson coupling to the massive vector bosons, a result of electroweak symmetry breaking. Some BSM models predict couplings to fermions and massive vector bosons different from those in the SM.

We compare the observations in data with the expectation for the SM Higgs boson by fitting two parameters, $\kappa _{\mathrm{V}}$ and $\kappa _{\mathrm{f}}$, where $\kappa _{\mathrm{V}} =\kappa _{{\mathrm{W}}} =\kappa _{\mathrm{{Z}}}$ is a common scaling factor for massive vector bosons, and $\kappa _{\mathrm{f}} =\kappa _\mathrm{{b}} =\kappa _\mathrm{{t}} =\kappa _{\tau }$ is a common scaling factor for fermions. We assume that $\varGamma _{\mathrm{BSM}}=0$. At leading order, all partial widths scale either as $\kappa _{\mathrm{V}} ^2$ or $\kappa _{\mathrm{f}} ^2$, except for $\varGamma _{\gamma \gamma }$. As discussed in Sect. 7.1, the partial width $\varGamma _{\gamma \gamma }$ is induced via loops with virtual $\mathrm{W}$ bosons or top quarks and scales as a function of both $\kappa _{\mathrm{V}}$ and $\kappa _{\mathrm{f}}$. For that reason, the $\mathrm{H} \rightarrow \gamma \gamma$ channel is the only channel being combined that is sensitive to the relative sign of $\kappa _{\mathrm{V}}$ and $\kappa _{\mathrm{f}}$.

Figure 9 shows the 2D likelihood scan over the $(\kappa _{\mathrm{V}},\kappa _{\mathrm{f}})$ parameter space. While Fig. 9 (left) allows for different signs of $\kappa _{\mathrm{V}}$ and $\kappa _{\mathrm{f}}$, Fig. 9 (right) constrains the scan to the $(+,+)$ quadrant that contains the SM expectation $(1,1)$. The $(-,-)$ and $(-,+)$ quadrants are not shown since they are degenerate with respect to the ones studied, with the implication that with the available analyses we can only probe whether $\kappa _{\mathrm{V}}$ and $\kappa _{\mathrm{f}}$ have the same sign or different signs. Studies of the production of a Higgs boson associated with a single top quark can, in principle, lift that degeneracy.

**Fig. 10:**
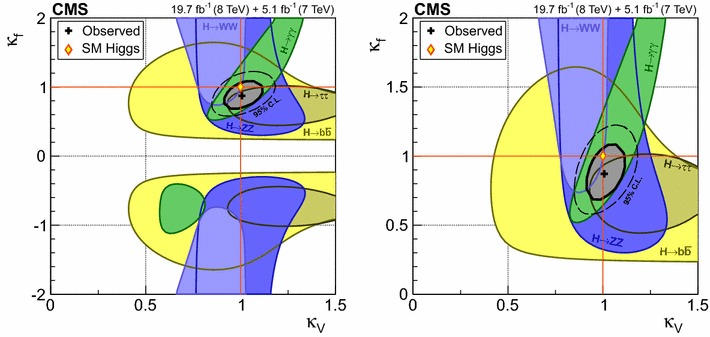
The 68 % CL confidence regions for individual channels (*coloured swaths*) and for the overall combination (*thick curve*) for the $\kappa _{\mathrm{V}}$ and $\kappa _{\mathrm{f}}$ parameters. The *cross* indicates the global best-fit values. The *dashed contour* bounds the 95 % CL confidence region for the combination. The *diamond* represents the SM expectation, $(\kappa _{\mathrm{V}}, \kappa _{\mathrm{f}})=(1,1)$. The *left plot* shows the likelihood scan in two quadrants $(+,+)$ and $(+,-)$, the *right plot* shows the positive quadrant only

**Fig. 11:**
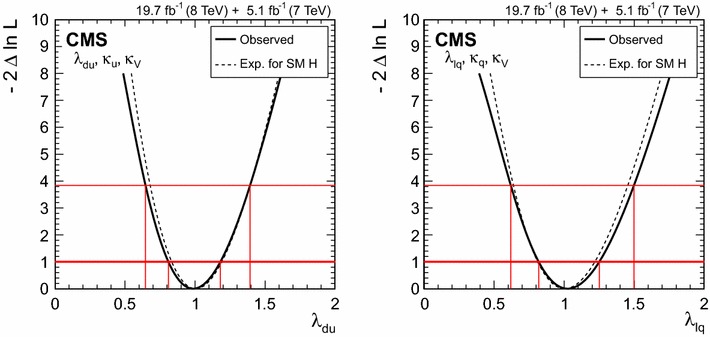
(*Left*) Likelihood scan versus ratio of couplings to down/up fermions, $\lambda _{\mathrm{{d} \mathrm{u}}}$, with the two other free coupling modifiers, $\kappa _{\mathrm{V}}$ and $\kappa _\mathrm{{u}}$, profiled together with all other nuisance parameters. (*Right*) Likelihood scan versus ratio of couplings to leptons and quarks, $\lambda _{\mathrm{\ell \mathrm{q}}}$, with the two other free coupling modifiers, $\kappa _{\mathrm{V}}$ and $\kappa _\mathrm{{q}}$, profiled together with all other nuisance parameters

In Fig. 9 the 68 %, 95 %, and 99.7 % CL confidence regions for $\kappa _{\mathrm{V}}$ and $\kappa _{\mathrm{f}}$ are shown with solid, dashed, and dotted curves, respectively. The data are compatible with the expectation for the standard model Higgs boson: the point $(\kappa _{\mathrm{V}},\kappa _{\mathrm{f}})=(1,1)$ is within the 68 % CL confidence region defined by the data. Because of the way these compatibility tests are constructed, any significant deviations from $(1,1)$ would not have a straightforward interpretation within the SM and would imply BSM physics; the scale and sign of the best-fit values in the case of significant deviations would guide us in identifying the most plausible BSM scenarios.

Figure 10 shows the results of this combined analysis in the different decay mode groups. The role and interplay of different channels is important. For example, Fig. 9 (left) shows a region in the $(+,-)$ quadrant, where $\kappa _{\mathrm{V}}$ and $\kappa _{\mathrm{f}}$ have opposite signs, which is excluded at the 95 % CL but not at the 99.7 % CL; it can be seen in Fig. 10 (left) how the combined exclusion in the $(+,-)$ quadrant is foremost due to the ability of the $\mathrm{H} \rightarrow \gamma \gamma$ decay to discern the relative sign between $\kappa _{\mathrm{V}}$ and $\kappa _{\mathrm{f}}$. This is due to the destructive interference between the amplitudes of the $\mathrm{W}$ loops and top quark loops in the $\mathrm{H} \rightarrow \gamma \gamma$ decay: $\kappa _{\gamma } ^2 \sim 1.59\;\kappa _{\mathrm{V}} ^2 - 0.66\;\kappa _{\mathrm{V}} \kappa _{\mathrm{f}} +0.07\;\kappa _{\mathrm{f}} ^2$; if $\kappa _{\mathrm{V}}$ and $\kappa _{\mathrm{f}}$ have opposite signs, the interference becomes constructive, leading to a larger $\mathrm{H} \rightarrow \gamma \gamma$ branching fraction. The shapes of the confidence regions for other decay channels are also interesting: the analyses of decays to massive vector bosons constrain $\kappa _{\mathrm{V}}$ better than $\kappa _{\mathrm{f}}$, whereas the analyses of decays to fermions constrain $\kappa _{\mathrm{f}}$ better than $\kappa _{\mathrm{V}}$. In the model used for this analysis, the total width scales as $\kappa _\mathrm{{H}} ^2 \sim 0.75\;\kappa _{\mathrm{f}} ^2 + 0.25\;\kappa _{\mathrm{V}} ^2$, reflecting the large expected contributions from the bottom quark and $\mathrm{W}$ boson.

The 95 % CL confidence intervals for $\kappa _{\mathrm{V}}$ and $\kappa _{\mathrm{f}}$, obtained from a scan where the other parameter is floated, are $[0.87,1.14]$ and $[0.63,1.15]$, respectively.

### Test for asymmetries in the couplings to fermions

In models with two Higgs doublets (2HDM) [203], the couplings of the neutral Higgs bosons to fermions can be substantially modified with respect to the couplings predicted for the SM Higgs boson. For example, in the minimal supersymmetric standard model [204], the couplings of neutral Higgs bosons to up-type and down-type fermions are modified, with the modification being the same for all three generations and for quarks and leptons. In more general 2HDMs, leptons can be made to virtually decouple from one Higgs boson that otherwise behaves in a SM-like way with respect to the $\mathrm{W}$ bosons, $\mathrm{Z}$ bosons, and quarks. Inspired by the possibility of such modifications to the fermion couplings, we perform two combinations in which we allow for different ratios of the couplings to down-type fermions and up-type fermions ($\lambda _{\mathrm{{d} \mathrm{u}}} = \kappa _\mathrm{{d}}/ \kappa _\mathrm{{u}}$) or different ratios of the couplings to leptons and quarks ($\lambda _{\mathrm{\ell \mathrm{q}}} = \kappa _{\ell }/ \kappa _\mathrm{{q}}$).

**Fig. 12:**
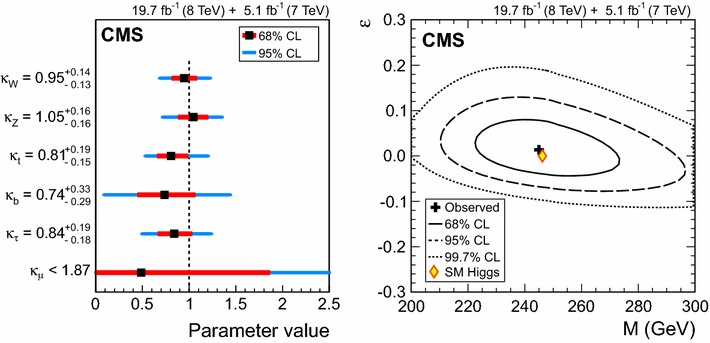
(*Left*) Results of likelihood scans for a model where the gluon and photon loop-induced interactions with the Higgs boson are resolved in terms of the couplings of other SM particles. The *inner bars* represent the 68 % CL confidence intervals while the *outer bars* represent the 95 % CL confidence intervals. When performing the scan for one parameter, the other parameters in the model are profiled. (*Right*) The 2D likelihood scan for the $M$ and $\epsilon$ parameters of the model detailed in the text. The *cross* indicates the best-fit values. The *solid*, *dashed*, and *dotted contours* show the 68 %, 95 %, and 99.7 % CL confidence regions, respectively. The *diamond* represents the SM expectation, $(M, \epsilon )=(v,0)$, where $v$ is the SM Higgs vacuum expectation value, $v=246.22\,\text {GeV}$

Figure 11 (left) shows the likelihood scan versus $\lambda _{\mathrm{{d} \mathrm{u}}}$, with $\kappa _{\mathrm{V}}$ and $\kappa _\mathrm{{u}}$ profiled together with all other nuisance parameters. Figure 11 (right) shows the likelihood scan versus $\lambda _{\mathrm{\ell \mathrm{q}}}$, with $\kappa _{\mathrm{V}}$ and $\kappa _\mathrm{{q}}$ profiled. Assuming that both $\lambda _{\mathrm{{d} \mathrm{u}}}$ and $\lambda _{\mathrm{\ell \mathrm{q}}}$ are positive, the 95 % CL confidence intervals are found to be $[0.65,1.39]$ and $[0.62,1.50]$, respectively. There is no evidence that different classes of fermions have different scaling factors.

### Test of the scaling of couplings with the masses of SM particles

Under the assumption that there are no interactions of the Higgs boson other than to the massive SM particles, the data allow a fit for deviations in $\kappa _{{\mathrm{W}}}$, $\kappa _{\mathrm{{Z}}}$, $\kappa _\mathrm{{b}}$, $\kappa _{\tau }$, $\kappa _\mathrm{{t}}$, and $\kappa _{\mathrm{\mu }}$. In this fit, the loop-induced processes ($\sigma _\mathrm{{g} \mathrm{g} \mathrm{H} }$, $\varGamma _\mathrm{{g} \mathrm{g} }$, and $\varGamma _{\gamma \gamma }$) are expressed in terms of the above tree-level $\kappa$ parameters and are scaled according to their SM loop structure. The result for this fit is displayed in Fig. 12 (left) and shows no significant deviations from the SM expectation. The small uncertainty in the $\kappa _\mathrm{{t}}$ parameter directly reflects the fact that in this model, the $\mathrm{g} \mathrm{g} \mathrm{H}$ production mode is being described in terms of $\kappa _\mathrm{{t}}$ and $\kappa _\mathrm{{b}}$, $\kappa _{\mathrm{{g}}} ^2 \sim 1.11\;\kappa _\mathrm{{t}} ^2 +0.01\;\kappa _\mathrm{{t}} \kappa _\mathrm{{b}}-0.12\;\kappa _\mathrm{{b}} ^2$, such that $\kappa _\mathrm{{b}}$ has a small contribution.

In the SM, the Yukawa coupling between the Higgs boson and the fermions, $$\lambda _{\mathrm{f}}$$, is proportional to the mass of the fermion, $$m_{\mathrm{f}}$$. This is in contrast with the coupling to weak bosons, $$g_{\mathrm{V}}$$, which involves the square of the mass of the weak boson, $$m_{\mathrm{V}}$$. With these differences in mind, it is possible to motivate a phenomenological parameterization relating the masses of the fermions and weak bosons to the corresponding $$\kappa $$ modifiers using two parameters, $$M$$ and $$\epsilon $$ [205, 206]. In such a model one has for each fermion $$\kappa _{\mathrm{f}} = v \; m_{\mathrm{f}}^{\epsilon } / M^{1+\epsilon }$$ and for each weak boson $$\kappa _{\mathrm{V}} = v \; m_{\mathrm{V}}^{2\epsilon } / M^{1+2\epsilon }$$, where $$v$$ is the SM Higgs boson vacuum expectation value, $$v=246.22\,\text {GeV} $$ [207]. The SM expectation, $$\kappa _{i}=1$$, is recovered when $$(M,\epsilon )=(v,0)$$. The parameter $$\epsilon $$ changes the power with which the coupling scales with the particle mass; if the couplings were independent of the masses of the particles, one would expect to find $$\epsilon =-1$$. To perform a fit to data, the particle mass values need to be specified. For leptons and weak bosons we have taken the values from Ref. [207]. For consistency with theoretical calculations used in setting the SM expectations, the top quark mass is taken to be $$172.5\,\text {GeV} $$. The bottom quark is evaluated at the scale of the Higgs boson mass, $$m_{\mathrm{b}}(m_\mathrm{{H}} =125.0\,\text {GeV} )=2.76\,\text {GeV} $$. In the fit, the mass parameters are treated as constants. The likelihood scan for $$(M,\epsilon )$$ is shown in Fig. 12 (right). It can be seen that the data do not significantly deviate from the SM expectation. The 95 % CL confidence intervals for the $$M$$ and $$\epsilon $$ parameters are $$[217,279]$$$$\,\text {GeV}$$ and $$[-0.054,0.100]$$, respectively.

**Fig. 13:**
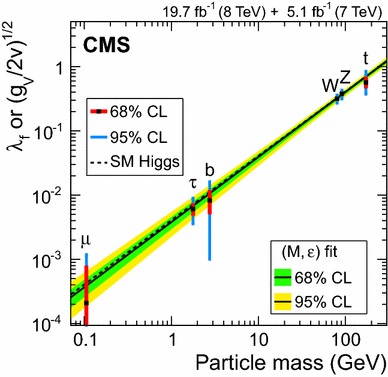
Graphical representation of the results obtained for the models considered in Fig. 12. The *dashed line* corresponds to the SM expectation. The points from the fit in Fig. 12 (*left*) are placed at particle mass values chosen as explained in the text. The ordinates are different for fermions and massive vector bosons to take into account the expected SM scaling of the coupling with mass, depending on the type of particle. The result of the $(M,\epsilon )$ fit from Fig. 12 (*right*) is shown as the *continuous line* while the *inner* and *outer bands* represent the 68 % and 95 % CL confidence regions

The results of the two fits above are plotted versus the particle masses in Fig. 13. While the choice of the mass values for the abscissas is discussed above, to be able to show both Yukawa and weak boson couplings in the same plot requires a transformation of the results of the $\kappa$ fit. Since $g_V \sim \kappa _{\mathrm{V}} 2m_{\mathrm{V}}^2/v$ and $\lambda _{\mathrm{f}} \sim \kappa _{\mathrm{f}} m_{\mathrm{f}}/v$, we have chosen to plot a “reduced” weak boson coupling, $\sqrt{g_{\mathrm{V}}/(2v)}=\kappa _{\mathrm{V}} ^{1/2} m_{\mathrm{V}}/v$. This choice allows fermion and weak boson results to be plotted together, as shown in Fig. 13, but implies that the uncertainties for $\kappa _{{\mathrm{W}}}$ and $\kappa _{\mathrm{{Z}}}$ will seem to be reduced. This simply reflects the square root in the change of variables and not any gain of information with respect to the $\kappa$ fit shown Fig. 12 (left). The result of the $(M,\epsilon )$ fit is shown in Fig. 13 as the band around the dashed line that represents the SM expectation. While the existing measurement of the scaling factor for the coupling of the boson with muons is clearly imprecise, the picture that arises from covering more than three orders of magnitude in particle mass is that the boson couples differently to the different particles and that those couplings are related to the mass of each particle. This is further supported by upper limits set in searches for $\mathrm{H} \rightarrow \mathrm{e}\mathrm{e}$ decays: when assuming the production cross sections predicted in the SM, the branching fraction is limited to be $\mathcal {B}(\mathrm{H} \rightarrow \mathrm{e}\mathrm{e} )<1.9\times 10^{-3}$ at the 95 % CL [30].

### Test for the presence of BSM particles in loops

The manifestation of BSM physics can considerably modify the Higgs boson phenomenology even if the underlying Higgs boson sector in the model remains unaltered. Processes that are loop-induced at leading order, such as the $\mathrm{H} \rightarrow \gamma \gamma$ decay and $\mathrm{g} \mathrm{g} \mathrm{H}$ production, can be particularly sensitive to the presence of new particles. Therefore, we combine and fit the data for the scale factors for these two processes, $\kappa _{\gamma }$ and $\kappa _{\mathrm{{g}}}$. The partial widths associated with the tree-level production processes and decay modes are assumed to be those expected in the SM, and the total width scales as $\kappa _\mathrm{{H}} ^2\sim 0.0857 \;\kappa _{\mathrm{{g}}} ^2 +0.0023\;\kappa _{\gamma } ^2 + 0.912$.

**Fig. 14:**
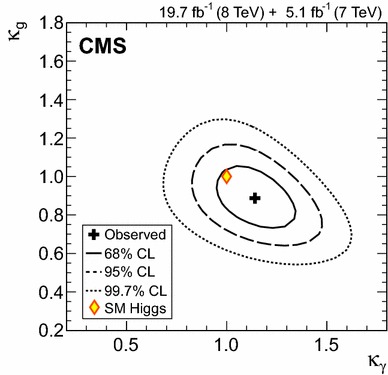
The 2D likelihood scan for the $\kappa _{\mathrm{{g}}}$ and $\kappa _{\gamma }$ parameters, assuming that $\varGamma _{\mathrm{BSM}}=0$. The *cross* indicates the best-fit values. The *solid*, *dashed*, and *dotted contours* show the 68 %, 95 %, and 99.7 % CL confidence regions, respectively. The *diamond* represents the SM expectation, $(\kappa _{\gamma }, \kappa _{\mathrm{{g}}})=(1,1)$. The partial widths associated with the tree-level production processes and decay modes are assumed to be unaltered ($\kappa = 1$)

**Fig. 15:**
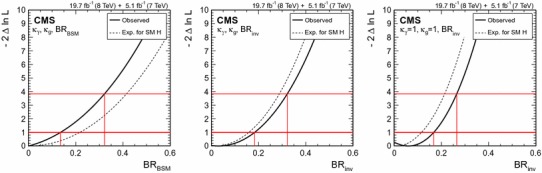
(*Left*) The likelihood scan versus $\mathrm{BR}_\mathrm{BSM} =\varGamma _{\mathrm{BSM}}/\varGamma _{\text {tot}}$. The *solid curve* represents the observation and the *dashed curve* indicates the expected median result in the presence of the SM Higgs boson. The partial widths associated with the tree-level production processes and decay modes are assumed to be as expected in the SM. (*Middle*) Result when also combining with data from the $\mathrm{H} ({\mathrm{inv}})$ searches, thus assuming that $\mathrm{BR}_\mathrm{BSM} =\mathrm{BR}_\mathrm{inv}$, i.e. that there are no undetected decays, $\mathrm{BR}_\text {undet} =0$. (*Right*) Result when further assuming that $\kappa _{\mathrm{{g}}} =\kappa _{\gamma } =1$ and combining with the data from the $\mathrm{H} ({\mathrm{inv}})$ searches

Figure 14 shows the 2D likelihood scan for the $\kappa _{\mathrm{{g}}}$ and $\kappa _{\gamma }$ parameters, assuming that $\varGamma _{\mathrm{BSM}}=0$. The results are compatible with the expectation for the SM Higgs boson, with the point $(\kappa _{\gamma }, \kappa _{\mathrm{{g}}})=(1,1)$ within the 68 % CL confidence region defined by the data. The best-fit point is $(\kappa _{\gamma }, \kappa _{\mathrm{{g}}})=(1.14, 0.89)$. The 95 % CL confidence interval for $\kappa _{\gamma }$, when profiling $\kappa _{\mathrm{{g}}}$ and all nuisance parameters, is $[0.89,1.40]$. For $\kappa _{\mathrm{{g}}}$, the 95 % CL confidence interval is $[0.69,1.11]$, when profiling $\kappa _{\gamma }$ and all other nuisance parameters.

Another way in which BSM physics may manifest itself is through the decay of the boson into BSM particles. To explore this possibility, we consider a further parameter that allows for a partial decay width into BSM particles, $\mathrm{BR}_\mathrm{BSM} =\varGamma _{\mathrm{BSM}}/\varGamma _{\text {tot}}$. In this case, the total width scales as $\kappa _\mathrm{{H}} ^2\sim (0.0857\;\kappa _{\mathrm{{g}}} ^2 + 0.0023\;\kappa _{\gamma } ^2 + 0.912)/(1-\mathrm{BR}_\mathrm{BSM})$.

Figure 15 (left) shows the likelihood scan versus $\mathrm{BR}_\mathrm{BSM}$, with $\kappa _{\mathrm{{g}}}$ and $\kappa _{\gamma }$ constrained to be positive and profiled together with all other nuisance parameters. While under the SM hypothesis the expected 95 % CL confidence interval for $\mathrm{BR}_\mathrm{BSM}$ is $[0.00,0.42]$, the data are such that the 95 % CL confidence interval for $\mathrm{BR}_\mathrm{BSM}$ is $[0.00,0.32]$, narrower than the expectation. The best fit in data also takes into account variations in $\kappa _{\mathrm{{g}}}$ and $\kappa _{\gamma }$, particularly the preference for $\kappa _{\mathrm{{g}}}$ smaller than unity in data, which influences the observed limit on $\mathrm{BR}_\mathrm{BSM}$.

A further step can be taken by also including the data from the searches for $\mathrm{H} ({\mathrm{inv}})$. The $\mathrm{H} ({\mathrm{inv}})$ searches reported an observed (expected) upper limit on $\mathrm{BR}_\mathrm{inv}$ of 0.58 (0.44) at the 95 % CL [28]. When including the $\mathrm{H} ({\mathrm{inv}})$ search results in the combined analysis, one can only obtain bounds assuming that there are no undetected decay modes, $\mathrm{BR}_\text {undet} =0$, i.e. that $\mathrm{BR}_\mathrm{BSM} =\mathrm{BR}_\mathrm{inv}$. The results for the likelihood scan as a function of $\mathrm{BR}_\mathrm{inv} (\mathrm{BR}_\text {undet} =0)$ when including the data from the $\mathrm{H} ({\mathrm{inv}})$ searches is shown in Fig. 15 (middle). The expected 95 % CL confidence interval for $\mathrm{BR}_\mathrm{inv} (\mathrm{BR}_\text {undet} =0)$ under the SM hypothesis is $[0.00,0.29]$, 31 % narrower than in the above case studied without the $\mathrm{H} ({\mathrm{inv}})$ data, a reflection of the added power of the $\mathrm{H} ({\mathrm{inv}})$ analysis. On the other hand, the 95 % CL confidence interval for $\mathrm{BR}_\mathrm{inv} (\mathrm{BR}_\text {undet} =0)$ in data is $[0.00,0.32]$, similar to the result obtained without including the $\mathrm{H} ({\mathrm{inv}})$ data, because the observed upper limit on $\mathrm{BR}_\mathrm{inv}$ was found to be larger than expected in those searches. It should be noted that the shape of the observed curve changes substantially and the inclusion of the $\mathrm{H} ({\mathrm{inv}})$ data leads to a very shallow minimum of the likelihood when $\mathrm{BR}_\mathrm{inv} (\mathrm{BR}_\text {undet} =0)=0.03$.

Finally, one may further set $\kappa _{\mathrm{{g}}} =\kappa _{\gamma } =1$, which effectively implies $\kappa _i=1$, i.e. assumes that the couplings to all SM particles with mass are as expected from the SM. From the combined analysis including the data from the $\mathrm{H} ({\mathrm{inv}})$ searches, we can thus obtain bounds on $\mathrm{BR}_\mathrm{inv} (\mathrm{BR}_\text {undet} =0,\kappa _i=1)$. The likelihood scan results are shown in Fig. 15 (right). The expected 95 % CL confidence interval for $\mathrm{BR}_\mathrm{inv} (\mathrm{BR}_\text {undet} =0,\kappa _i=1)$ under the SM hypothesis is $[0.00,0.21]$, which is 28 % narrower than in the previous paragraph, a reflection of the total width now being fixed to the SM expectation. The 95 % CL confidence interval for $\mathrm{BR}_\mathrm{inv} (\mathrm{BR}_\text {undet} =0,\kappa _i=1)$ in data is $[0.00,0.27]$, showing again a shallow minimum of the likelihood when $\mathrm{BR}_\mathrm{inv} (\mathrm{BR}_\text {undet} =0,\kappa _i=1)=0.06$.

The results obtained from the different combined analyses presented in Fig. 15 show the added value from combining the $\mathrm{H} ({\mathrm{inv}})$ searches with the visible decay measurements, with the expected 95 % CL combined upper limit on $\mathrm{BR}_\mathrm{inv}$ being up to a factor of two smaller than either, depending on the assumptions made.

**Fig. 16:**
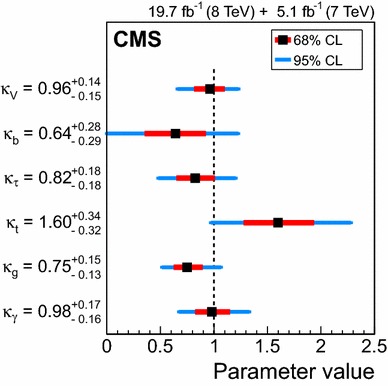
Likelihood scans for parameters in a model with coupling scaling factors for the SM particles, one coupling at a time while profiling the remaining five together with all other nuisance parameters; from *top* to *bottom*: $\kappa _{\mathrm{V}}$ ($\mathrm{W}$ and $\mathrm{Z}$ bosons), $\kappa _\mathrm{{b}}$ (bottom quarks), $\kappa _{\tau }$ (tau leptons), $\kappa _\mathrm{{t}}$ (top quarks), $\kappa _{\mathrm{{g}}}$ (gluons; effective coupling), and $\kappa _{\gamma }$ (photons; effective coupling). The *inner bars* represent the 68 % CL confidence intervals while the *outer bars* represent the 95 % CL confidence intervals

**Fig. 17:**
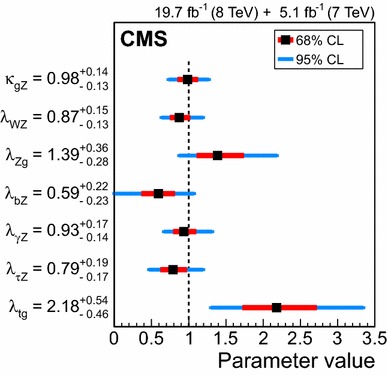
Likelihood scans for parameters in a model without assumptions on the total width and with six coupling modifier ratios, one parameter at a time while profiling the remaining six together with all other nuisance parameters; from *top* to *bottom*: $\kappa _\mathrm{{g} \mathrm{Z}}$ ($=\kappa _{\mathrm{{g}}} \kappa _{\mathrm{{Z}}}/\kappa _\mathrm{{H}}$), $\lambda _{{\mathrm{W}\mathrm{Z}}}$ ($=\kappa _{{\mathrm{W}}}/\kappa _{\mathrm{{Z}}}$), $\lambda _\mathrm{{Z}\mathrm{g}}$ ($=\kappa _{\mathrm{{Z}}}/\kappa _{\mathrm{{g}}}$), $\lambda _\mathrm{{b}\mathrm{Z}}$ ($=\kappa _\mathrm{{b}}/\kappa _{\mathrm{{Z}}}$), $\lambda _{\gamma \mathrm{Z}}$ ($=\kappa _{\gamma }/\kappa _{\mathrm{{Z}}}$), $\lambda _{\tau \mathrm{Z}}$ ($=\kappa _{\tau }/\kappa _{\mathrm{{Z}}}$), and $\lambda _\mathrm{{t}\mathrm{g}}$ ($=\kappa _\mathrm{{t}}/\kappa _{\mathrm{{g}}}$). The *inner bars* represent the 68 % CL confidence intervals while the *outer bars* represent the 95 % CL confidence intervals

### Test of a model with scaling factors for SM particles

After having examined the possibility for BSM physics to manifest itself in loop-induced couplings while fixing all the other scaling factors, we now release the latter assumption. For that, we explore a model with six independent coupling modifiers and make the following assumptions:

- The couplings to $\mathrm{W}$ and $\mathrm{Z}$ bosons scale with a common parameter $\kappa _{\mathrm{V}} =\kappa _{{\mathrm{W}}} =\kappa _{\mathrm{{Z}}}$.
- The couplings to third generation fermions, i.e. the bottom quark, tau lepton, and top quark, scale independently with $\kappa _\mathrm{{b}}$, $\kappa _{\tau }$, and $\kappa _\mathrm{{t}}$, respectively.
- The effective couplings to gluons and photons, induced by loop diagrams, scale with free parameters $\kappa _{\mathrm{{g}}}$ and $\kappa _{\gamma }$, respectively.
- The partial width $\varGamma _{\mathrm{BSM}}$ is zero.

A likelihood scan for each of the six coupling modifiers is performed while profiling the other five, together with all other nuisance parameters; the results are shown in Fig. 16. With this set of parameters, the $\mathrm{g} \mathrm{g} \mathrm{H}$-production measurements will constrain $\kappa _{\mathrm{{g}}}$, leaving the measurements of $\mathrm{t}\mathrm{t}\mathrm{H}$ production to constrain $\kappa _\mathrm{{t}}$, which explains the best-fit value, $\kappa _\mathrm{{t}} =1.60 ^{+0.34}_{-0.32}$. The current data do not show any statistically significant deviation with respect to the SM Higgs boson hypothesis. For every $\kappa _i$ probed, the measured 95 % CL confidence interval contains the SM expectation, $\kappa _i=1$. A goodness-of-fit test between the parameters measured in this model and the SM prediction yields a $\chi ^{2}/\text {dof} = 7.5/6$, which corresponds to an asymptotic $p\text {-value}$ of 0.28.

### Test of a general model without assumptions on the total width

Given the comprehensiveness of the set of analyses being combined, we can explore the most general model proposed in Ref. [171], which makes no assumptions on the scaling of the total width. In this model, the total width is not rescaled according to the different $\kappa _{i}$ values as a dependent parameter, but is rather left as a free parameter, embedded in $\kappa _\mathrm{{g} \mathrm{Z}} =\kappa _{\mathrm{{g}}} \kappa _{\mathrm{{Z}}}/\kappa _\mathrm{{H}}$. All other parameters of interest are expressed as ratios between coupling scaling factors, $\lambda _{ij}=\kappa _{i}/\kappa _{j}$.

**Fig. 18:**
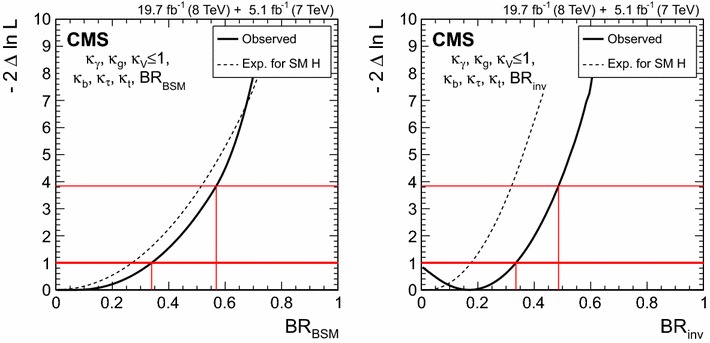
(*Left*) Likelihood scan versus $\mathrm{BR}_\mathrm{BSM} =\varGamma _{\mathrm{BSM}}/\varGamma _{\text {tot}}$. The *solid curve* represents the observation in data and the *dashed curve* indicates the expected median result in the presence of the SM Higgs boson. The modifiers for both the tree-level and loop-induced couplings are profiled, but the couplings to the electroweak bosons are assumed to be bounded by the SM expectation ($\kappa _{\mathrm{V}} \le 1$). (*Right*) Result when also combining with data from the $\mathrm{H} ({\mathrm{inv}})$ searches, thus assuming that $\mathrm{BR}_\mathrm{BSM} =\mathrm{BR}_\mathrm{inv}$, i.e. $\mathrm{BR}_\text {undet} =0$

A likelihood scan for each of the parameters $\kappa _\mathrm{{g} \mathrm{Z}}$, $\lambda _{{\mathrm{W}\mathrm{Z}}}$, $\lambda _\mathrm{{Z}\mathrm{g}}$, $\lambda _\mathrm{{b}\mathrm{Z}}$, $\lambda _{\gamma \mathrm{Z}}$, $\lambda _{\tau \mathrm{Z}}$, and $\lambda _\mathrm{{t}\mathrm{g}}$ is performed while profiling the other six, together with all other nuisance parameters. The results are shown in Fig. 17 and are in line with those found in Sect. 7.6.

**Fig. 19:**
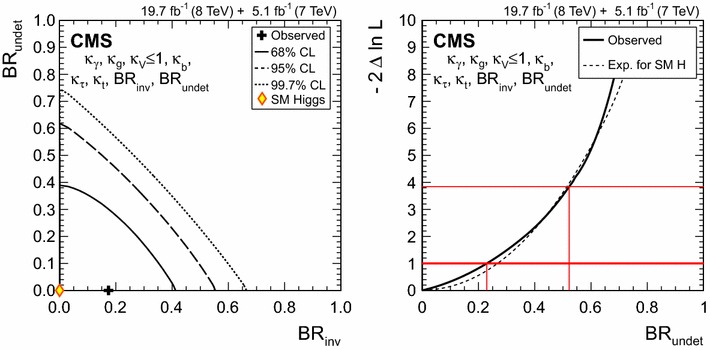
(*Left*) The 2D likelihood scan for the $\mathrm{BR}_\mathrm{inv}$ and $\mathrm{BR}_\text {undet}$ parameters for a combined analysis of the $\mathrm{H} ({\mathrm{inv}})$ search data and visible decay channels. The *cross* indicates the best-fit values. The *solid*, *dashed*, and *dotted contours* show the 68 %, 95 %, and 99.7 % CL confidence regions, respectively. The *diamond* represents the SM expectation, $(\mathrm{BR}_\mathrm{inv}, \mathrm{BR}_\text {undet})=(0,0)$. (*Right*) The likelihood scan versus $\mathrm{BR}_\text {undet}$. The *solid curve* represents the observation in data and the *dashed curve* indicates the expected median result in the presence of the SM Higgs boson. $\mathrm{BR}_\mathrm{inv}$ is constrained by the data from the $\mathrm{H} ({\mathrm{inv}})$ searches and modifiers for both the tree-level and loop-induced couplings are profiled, but the couplings to the electroweak bosons are assumed to be bounded by the SM expectation ($\kappa _{\mathrm{V}} \le 1$)

### Constraints on $\mathrm{BR}_\mathrm{BSM}$ in a scenario with free couplings

An alternative and similarly general scenario can be built by allowing for $\varGamma _{\mathrm{BSM}}>0$. In order to avoid the degeneracy through which the total width and the coupling scaling factors can compensate each other, we constrain $\kappa _{\mathrm{V}} \le 1$, a requirement that holds in a wide class of models, namely in any model with an arbitrary number of Higgs doublets, with and without additional Higgs singlets [171]. The model has the following parameters: $\kappa _{\mathrm{V}}$, $\kappa _\mathrm{{b}}$, $\kappa _{\tau }$, $\kappa _\mathrm{{t}}$, $\kappa _{\mathrm{{g}}}$, $\kappa _{\gamma }$, and $\mathrm{BR}_\mathrm{BSM}$. This is a much more general treatment than that performed in Sect. 7.5, where only the loop-induced couplings to photons and gluons were allowed to deviate from the SM expectation. As in Sect. 7.5, this model also allows for a combined analysis with the data from the $\mathrm{H} ({\mathrm{inv}})$ searches.

Figure 18 (left) shows the likelihood scan versus $\mathrm{BR}_\mathrm{BSM}$ derived in this scenario, while profiling all the other coupling modifiers and nuisance parameters. Within these assumptions, the 95 % CL confidence interval for $\mathrm{BR}_\mathrm{BSM}$ in data is $[0.00,0.57]$, while the expected interval for the SM hypothesis is $[0.00,0.52]$.

Assuming that there are no undetected decay modes, $\mathrm{BR}_\text {undet} =0$, it follows that $\mathrm{BR}_\mathrm{BSM} =\mathrm{BR}_\mathrm{inv}$ and the data from the searches for $\mathrm{H} ({\mathrm{inv}})$ can be combined with the data from the other channels to set bounds on $\mathrm{BR}_\mathrm{inv}$. The likelihood scan for such a model and combination is shown in Fig. 18 (right). The 95 % CL confidence interval for $\mathrm{BR}_\mathrm{inv}$ in data is $[0.00,0.49]$, while the expected interval for the SM hypothesis is $[0.00,0.32]$. The difference between the expected and observed confidence intervals reflects the results of the $\mathrm{H} ({\mathrm{inv}})$ analysis that reported an observed (expected) upper limit on $\mathrm{BR}_\mathrm{inv}$ of 0.58 (0.44) at the 95 % CL [28].

Finally, instead of simply assuming $\mathrm{BR}_\text {undet} =0$, a simultaneous fit for $\mathrm{BR}_\mathrm{inv}$ and $\mathrm{BR}_\text {undet}$ is performed. In this case, the data from the $\mathrm{H} ({\mathrm{inv}})$ searches constrains $\mathrm{BR}_\mathrm{inv}$, while the visible decays constrain $\mathrm{BR}_\mathrm{BSM} =\mathrm{BR}_\mathrm{inv} +\mathrm{BR}_\text {undet}$. The 2D likelihood scan for $(\mathrm{BR}_\mathrm{inv},\mathrm{BR}_\text {undet})$ is shown in Fig. 19 (left), while Fig. 19 (right) shows the likelihood scan for $\mathrm{BR}_\text {undet}$ when profiling all other parameters, $\mathrm{BR}_\mathrm{inv}$ included. The 95 % CL confidence interval for $\mathrm{BR}_\text {undet}$ in data is $[0.00,0.52]$, while the expected interval for the SM hypothesis is $[0.00,0.51]$.

### Summary of tests of the compatibility of the data with the SM Higgs boson couplings

**Fig. 20:**
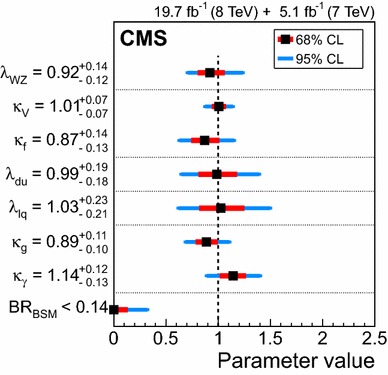
Summary plot of likelihood scan results for the different parameters of interest in benchmark models from Ref. [171] separated by *dotted lines*. The $\mathrm{BR}_\mathrm{BSM}$ value at the *bottom* is obtained for the model with three parameters $(\kappa _{\mathrm{{g}}},\kappa _{\gamma },\mathrm{BR}_\mathrm{BSM})$. The *inner bars* represent the 68 % CL confidence intervals while the *outer bars* represent the 95 % CL confidence intervals

Figure 20 summarizes the results for the benchmark scenarios of Ref. [171] with fewest parameters and shows that, in those benchmarks, all results are compatible with the SM expectations.

A much more comprehensive overview of the searches performed for deviations from the SM Higgs boson expectation is provided in Table 12, where all results obtained in this section are summarized.

No statistically significant deviations are observed with respect to the SM Higgs boson expectation.

## Summary

Properties of the Higgs boson with mass near 125$\,\text {GeV}$ are measured in proton-proton collisions with the CMS experiment at the LHC. Comprehensive sets of production and decay measurements are combined. The decay channels include $\gamma \gamma$, $\mathrm{Z}\mathrm{Z}$, $\mathrm{W}\mathrm{W}$, $\tau \tau$, $\mathrm{b} \mathrm{b}$, and $\mu \mu$ pairs. The data samples were collected in 2011 and 2012 and correspond to integrated luminosities of up to 5.1$\,\text {fb}^\text {-1}$ at 7$\,\text {TeV}$ and up to 19.7$\,\text {fb}^\text {-1}$ at 8$\,\text {TeV}$. From the high-resolution $\gamma \gamma$ and $\mathrm{Z}\mathrm{Z}$ channels, the mass of the Higgs boson is determined to be $125.02\,^{+0.26}_{-0.27} \,\text {(stat)} \,^{+0.14}_{-0.15} \,\text {(syst)} \,\text {GeV}$. For this mass value, the event yields obtained in the different analyses tagging specific decay channels and production mechanisms are consistent with those expected for the standard model Higgs boson. The combined best-fit signal relative to the standard model expectation is $1.00\,\pm 0.09\,\text {(stat)} \,^{+0.08}_{-0.07}\,\text {(theo)} \,\pm 0.07\,\text {(syst)}$ at the measured mass. The couplings of the Higgs boson are probed for deviations in magnitude from the standard model predictions in multiple ways, including searches for invisible and undetected decays. No significant deviations are found.
